# Sea lice (*Lepeophtheirus salmonis*) life stage impacts atlantic salmon transcriptomic responses under different thermal profiles

**DOI:** 10.3389/fgene.2025.1633603

**Published:** 2025-07-29

**Authors:** Reza Ghanei-Motlagh, Wenlong Cai, Jordan D. Poley, Shona K. Whyte, Amber F. Garber, Mark D. Fast

**Affiliations:** ^1^ Hoplite Research Lab, Department of Pathology and Microbiology, Atlantic Veterinary College, University of Prince Edward Island, Charlottetown, PE, Canada; ^2^ Huntsman Marine Science Centre, St. Andrews, NB, Canada; ^3^ Department of Infectious Diseases and Public Health, Jockey Club College of Veterinary Medicine and Life Sciences, City University of Hong Kong, Hong Kong, Hong Kong SAR, China; ^4^ Onda, Souris, PE, Canada

**Keywords:** parasitic infestation, family-specific transcriptomics, genetic background, infestation stage, selective breeding

## Abstract

Sea lice (*Lepeophtheirus salmonis*) infestation continues to pose a persistent and escalating challenge to the global salmon aquaculture industry. Given the complexity of host-parasite interactions, family-based transcriptomic studies provide crucial insights into genetic variation in host responses to sea lice, potentially guiding the development of selective breeding programs to manage parasite resistance in Atlantic salmon. This study investigated global gene expression (transcriptomic) responses of the skin and head kidney of Atlantic salmon (*Salmo salar*) from different families following infestation at two distinct stages of sea lice, chalimus II and adult, under varying temperature conditions (10°C and 20°C). RNA sequencing results revealed consistent expression of lice-responsive genes across different families under varying thermal conditions, which allowed the identification of potential biomarkers associated with adult-stage compared to chalimus-stage infestations. Our findings highlight critical physiological disruptions in salmon infested with advanced (adult) stages of lice, including uncontrolled and persistent inflammation, dampened/dysregulated immune responses, and impaired tissue repair at attachment sites. This study provides a comprehensive analysis of the transcriptomic responses of Atlantic salmon to different developmental stages of sea lice under specific temperature conditions (10°C and 20°C), and identifies several novel molecular markers from RNA-seq analysis that may be instrumental in developing targeted control strategies for this economically important parasite.

## 1 Introduction

Atlantic salmon (*Salmo salar*) aquaculture has witnessed remarkable growth over the past few decades, becoming a critical contributor to global seafood production (FAO, 2024). This growth, however, has been accompanied by persistent challenges that threaten the sustainability and economic viability of the industry. The ectoparasitic *Lepeophtheirus salmonis*, commonly known as the salmon louse, is currently the most significant threat to the health and productivity of Atlantic salmon farming (Whyte et al., 2014; Ugelvik and Dalvin, 2022). The economic impact of sea lice infestations on the global salmon farming industry is estimated to exceed $1 billion annually, encompassing reduced growth performance, expenses associated with treatment and prevention strategies, and direct losses through morbidity (Barker, et al., 2019). Sea lice infestation has also raised significant concerns regarding welfare of infested fish on the farm due to practices associated with delousing and potential ecological impacts on wild salmon populations adjacent to farming operations (Larsen and Vormedal, 2021).

The life cycle of *L. salmonis* comprises free-swimming planktonic stages (nauplius I, nauplius II) followed by the transitionary planktonic and first parasitic stage, the copepodid. Additional parasitic stages exist (chalimus I, chalimus II, pre-adult I, pre-adult II and adult) in which the parasite attaches to the host fish and feeds on the mucus, skin cells, and blood (Hamre et al., 2013). The chalimus stages are distinguished by a frontal filament that enables their secure attachment to the host, whereas the mobile pre-adult and adult stages are capable of moving across the fish’s surface, inflicting more substantial tissue damage through their aggregatory feeding activities (Fast and Braden, 2022). These various developmental stages exhibit stage-specific patterns of host-parasite interaction, with varying impacts on the host’s physiological and immunological responses (Dalvin et al., 2020; Øvergård et al., 2023). The progressive damage caused during sea lice development ultimately results in skin lesions, secondary infections, osmoregulatory disruption, and although rarer, mortality in heavily infested fish (Rodger et al., 2022).

Previous studies have revealed complex gene expression patterns that evolve throughout the course of infestation with sea lice and vary according to the parasite developmental stage (Sutherland et al., 2014; Tadiso et al., 2011). As the primary interface between the host and parasite, the salmon skin exhibits pronounced transcriptomic alterations following sea lice attachment (Krasnov et al., 2012; Sutherland et al., 2014; Holm et al., 2015; Robledo et al., 2018; Umasuthan et al., 2020; Caballero-Solares et al., 2022; Cai et al., 2022; Cai et al., 2024; Ugelvik and Dalvin, 2022; Øvergård et al., 2023). Local responses at mucosal surfaces, particularly the skin, involve upregulation of innate immune genes such as interleukins (e.g., *il1b*, *il8*), acute phase proteins, and antimicrobial peptides, often accompanied by inflammation and epithelial disruption (Skugor et al., 2008; Braden et al., 2015). These responses are typically more pronounced during the early chalimus stage, when the lice are attached to the epithelium, but they may be suppressed or dysregulated as the lice mature. In addition to localized responses at attachment sites, sea lice infestation induces systemic alterations, including modulation of immune markers, stress-related genes (e.g., heat shock proteins), and metabolic pathways, in internal tissues such as the head kidney, which functions as the primary hematopoietic organ in teleost fish (Skugor et al., 2008; Tadiso et al., 2011; Robledo et al., 2019; Øvergård et al., 2023; Valenzuela-Muñozet al., 2023; Zhong et al., 2023). Comparative transcriptomic studies have identified remarkable variation in response patterns between resistant and susceptible salmon species, providing insights into potential genetic determinants of resistance (Robledo et al., 2018; Robledo et al., 2019; Braden et al., 2023; Valenzuela-Muñoz et al., 2023; Cáceres et al., 2024; Salisbury et al., 2024). These findings hold significant implications for selective breeding programs focused on improving natural resistance to sea lice in farmed salmon populations.

In addition to the direct effects of sea lice on salmon, environmental stressors, particularly temperature fluctuations, can compound the impacts of sea lice on salmon health and immune function (Alfonso et al., 2021; Islam et al., 2022; Scharsack and Franke, 2022). Global warming and increased water temperatures are anticipated to reshape the dynamics of salmon aquaculture by simultaneously affecting fish physiology and sea lice biology (Hamre et al., 2019; Beemelmanns et al., 2021). Earlier research has indicated that elevated temperatures may exacerbate the severity of infectious diseases in Atlantic salmon, potentially altering salmon fitness, immune function and metabolic responses (Shi et al., 2019; Medcalf et al., 2021; Franke et al., 2024). In the context of *L. salmonis*, higher temperature has been shown to influence the parasite’s life cycle, development, and settlement success while modifying host-parasite interactions and efficacy of louse reduction, leading to enhanced infestation rates and more detrimental impacts on salmon health (Overton et al., 2019; Godwin et al., 2020; Ugelvik et al., 2022; Nilsson et al., 2023; Oldham et al., 2023). Furthermore, high temperatures can alter the stress signaling pathways and the expression of immune-related genes, potentially compromising the salmon’s immunological defenses against sea lice (Jensen et al., 2015; Nuez-Ortín et al., 2018).

The effects of infestation with different developmental stages of sea lice have been previously investigated on salmon individuals (infested *versus* healthy) using gene expression analyses (Skugor et al., 2008; Tadiso et al., 2011; Sutherland et al., 2014; Ugelvik and Dalvin, 2022; Øvergård et al., 2023). Prior studies suggest that systemic responses are often delayed or subdued, potentially reflecting immune evasion strategies employed by the parasite (Skugor et al., 2008; Tadiso et al., 2011; Ugelvik and Dalvin, 2022; Øvergård et al., 2023). These stage-specific and compartmentalized host responses underscore the complexity of the salmon-lice interaction and highlight the need for integrated analyses to identify meaningful biomarkers associated with the parasite’s developmental stages. However, transcriptomic differences between fish parasitized with chalimus and adult stages of *L. salmonis* have not been directly compared, particularly across multiple salmon families to identify consistently expressed genes associated with sea lice infestation. The present study therefore aims to remove inherent variation in this system by profiling the transcriptomic responses of the skin and head kidney of lice-infested Atlantic salmon at the family level under normal and elevated temperature conditions. The findings of this study are expected to significantly contribute to our understanding of the molecular mechanisms and biological pathways underlying the salmon’s response to sea lice infestation.

## 2 Materials and methods

### 2.1 Fish husbandry

The experimental population consisted of North American Atlantic salmon from 20 families (100 fish per family), produced from 16 sires and 16 dams from three different year classes in a larger breeding program with anticipated variable performance for sea lice resistance. All the smolts were bred and reared at the Huntsman Marine Science Centre (Huntsman Marine) in St. Andrews, New Brunswick, Canada. At approximately 2900–3000 degree days (dd, 16.5 g mean weight), all fish were anesthetized with tricaine methane sulphonate (MS-222, 100 mg/L) before insertion of a passive integrated transponder (PIT) tag to distinguish between the families. Fish were allowed to recover in oxygenated water, transferred to a single tank (7.5 m^3^) with additional PIT tagged salmon and reared in a semi-closed recirculating freshwater system with ambient light and water temperature maintained 1°C–3°C above ambient. Once fish had reached smolt-size (>50 g mean weight, ∼4476 dd), they were anesthetized (MS-222; 150 mg/L) and intraperitoneally vaccinated (100 µL/fish) with Micro Forte V *II* ^™^ (multivalent vaccine containing inactivated ISA) and Renogen^™^ (Merck Animal Health, Canada). These fish were subsequently smolted remaining in a single 7.5 m^3^ tank.

Fish were fed a commercial diet (Skretting, Canada) using automatic feeders with ambient photoperiod. Using the post-smolt body mass and families, they were randomly distributed to 8 tanks (1.3 m^3^) according to their body mass and family groups at a stocking density of 120 fish per tank (6 fish per family, 20 families per tank). Fish were maintained at 6°C then 2°C for ∼6 months (19 March to 15 August 2020) due to COVID-19 shut down (constraints in sourcing sea lice for the challenge). At this point, the salmon were acclimated to ambient temperature from 16 August to 31 October 2020 (ranging from approximately 14°C in mid‐August to 11°C in later October) and then gradually adjusted to 10°C ± 1°C for 10 days (01 November to 10 November 2020). After initial acclimation for 10 days, there was a daily increase of 1°C in the temperature of 4 out of the 8 tanks until it reached 20°C ± 1°C (11 November to 21 November 2020). Two temperature conditions were selected to investigate the transcriptomic responses of Atlantic salmon infested with adult *versus* chalimus stages of sea lice: 10°C, which falls within the species’ optimal physiological range, and 20°C, a temperature known to induce thermal stress (Beemelmanns et al., 2021). *Lepeophtheirus salmonis* has the potential to infest Atlantic salmon across a temperature range of approximately 6°C–22°C (Hamre et al., 2019; Godwin et al., 2020).

### 2.2 Lice challenge and sample collection

Sea lice (i.e., gravid females) were collected from Atlantic salmon reared in a marine cage site in the Bay of Fundy, New Brunswick, Canada. Egg strings were removed from the females and incubated in two incubators at 12.3°C for 8 days to reach the infective copepodid stage. On the 9th day (113.1 dd), the incubation temperature was raised to 15°C in both incubators and then adjusted by 1°C per hour to 10°C or 20°C in either incubator, corresponding to the temperatures in fish tanks. The copepodids were then used to infest the Atlantic salmon at 100 copepodids per fish for 1 h in each tank. The status of lice development was monitored throughout the experimental challenge. When the copepodids developed to the chalimus II larvae (110 dd), fish in half of the tanks (2 tanks per temperature) were subjected to biometric assessment and lice counting. The lice were allowed to develop to the adult stage (360 dd) in the remaining four tanks, after which the same procedure was conducted (2 tanks per temperature). Lice were counted on all body surfaces of fish including gills (removed for counting with a dissecting scope in the chalimus stage). All fish in each tank were included in lice counting (at individual sampling events) except mortalities, which occurred outside of those dates. Immediately following lice counting, two tissue samples (skin and head kidney) were collected from fish parasitized with intermediate/adult forms and preserved in RNAlater solution for further molecular analyses. Tissue samples were collected from the anterior head kidney on the right side and skin located immediately behind the dorsal fin (10 mm × 10 mm) of each Atlantic salmon. Lice abundance (lice per fish) was separately calculated for fish parasitized with chalimus or adult stages.

### 2.3 RNA extraction and library preparation

Portions of the tissues (∼30 mg) collected were each subjected to RNA extraction. Total RNA extraction was carried out using TRIzol-chloroform phase separation followed by DNase treatment and purification of the aqueous phase with the RNA Clean & Concentrator Kit (Zymo Research, CA, United States) according to the manufacturer’s instructions. The concentration and purity of isolated RNA samples were measured using NanoDrop spectrophotometry (ND 2000; Thermo Scientific), after which the RNA integrity was assessed initially by 1.5% agarose gel electrophoresis and subsequently using a Bioanalyzer (BioRad) (Cai et al., 2024). RNA samples with A260/A280 ≥ 1.9 and A260/A230 ≥ 1.7 were used for subsequent analyses. RNA samples from individuals of five families of different lice burden (F175, F265, F292, F361, F419) were selected for RNA sequencing in this study (3–5 samples per family). The RNA samples were standardized to 100 ng/μL and then used for RNA-Seq library construction using the TruSeq Stranded mRNA Library Prep Kit (Illumina, CA, United States) as recommended by manufacturer’s instructions. All libraries were sequenced (2 × 100 bp) by the Illumina NovaSeq 6000 S4 platform according to the standard Illumina protocol at Genome Québec (Montreal, Canada). The raw sequencing reads generated were deposited in Sequence Read Archive (SRA) database of NCBI (BioProject accession number: PRJNA1256531).

### 2.4 RNA sequencing data analysis

Quality control and preprocessing of the raw sequence (fastq) data were performed using FastQC v0.15.3. Residual adaptor sequences and low-quality bases were trimmed using Trimmomatic v0.36 (phred33, illuminaclip:TruSeq3-PE.fa:2:30:10, leading:3, trailing:3, slidingwindow:4:15 minlen:36). STAR v.2.7.9a was applied to map the trimmed reads to the latest Atlantic salmon genome assembly (Ssal_v3.1, GenBank accession no: GCF_905237065.1) and sort the obtained binary alignment map (BAM) files (Salisbury et al., 2024). In order to generate a count matrix from the estimated reads mapped to individual genes, featureCounts was used as part of the Subread package v1.6.5 (Liao et al., 2014). The edgeR package (classic approach) was used to perform pairwise comparisons between representatives of families parasitized with adult *versus* chalimus stages of sea lice (3–5 samples per family). The following workflow was implemented in edgeR: calcNormFactors (method = “TMM”) for normalization, estimateDisp () for dispersion estimation, and exactTest () for differential expression analysis. Genes were considered significantly differentially expressed based on the following thresholds: |log_2_-fold change| > 1.25, adjusted *p*-value <0.05 and |log-transformed counts per million reads| > 1.00. Moreover, interactions between differentially expressed genes (DEGs) obtained from pairwise comparisons were also investigated. Principal component analysis (PCA) was used using the prcomp function in R to identify variations among RNA-seq samples corresponding to each pairwise comparison. The RNA-seq datasets that were identified as outliers were excluded from downstream analyses. The hierarchical clustering of shared DEGs among different pairwise comparisons was performed using “pheatmap” function in R.

### 2.5 Gene ontology and KEGG enrichment analyses

Gene annotation was performed using the R package AnnotationHub (orgDb: *S. salar*, database no: AH114250), and gene ontology (GO) and Kyoto Encyclopedia of Genes and Genomes (KEGG) enrichment analyses of fully annotated up- and downregulated DEGs were conducted using ClueGO plug-in (v2.5.10) in Cytoscape v3.10.2 to visualize the non-redundant biological terms in a functionally grouped network (Shannon et al., 2003; Bindea et al., 2009). The ClueGO enrichment analyses were implemented using the following options/adjustments: pathway *p*-value cut-off <0.05, kappa score: 0.4 and right-sided hypergeometric test with Benjamini–Hochberg *p* value correction. To reduce complexity, the GO term fusion option was applied in most comparisons. However, a non-fusion approach was used to generate a more comprehensive overview of leading and sub-leading GO terms associated with shared DEGs across pairwise comparisons. The leading GO terms coupled with all genes associated with each GO group were also extracted for further analyses. The enriched (over-represented) leading GO terms and KEGG pathways obtained for comparisons related to temperature (for both paired-related and shared DEGs) and infestation stage of lice (for only shared DEGs) were categorized into different functional themes based on their parent GO terms as well as DEGs and sub-leading GO terms and KEGG pathways involved in each GO group: 1) metabolism, protein interaction and gene regulation, 2) cellular and developmental dynamics, 3) signal transduction and stimulus response, 4) enzymatic functions, and 5) molecular localization and transport. Terms/pathways associated with metabolic (either anabolic or catabolic) processes/pathways, transcriptional and replication regulation, protein-containing complex and protein-protein interactions were grouped under the functional theme “metabolism, protein interaction and gene regulation.” The GO terms “organelle envelope” and “intracellular organelle,” which were interconnected with metabolism-related terms and involved genes associated with metabolic processes, were also classified under this functional theme. Terms/pathways related to cellular process, cellular anatomical structure, developmental process and tissue repair were categorized as ‘cellular and developmental dynamics’. The theme ‘signal transduction and stimulus response’ included terms/pathways related to cell signaling pathways and responses to various stimuli and stressors. The functional theme “enzymatic functions” encompassed terms/pathways correlated with catalytic enzyme activities, while terms/pathways involved in intracellular localization and molecular transport mechanisms were classified under “molecular localization and transport.” The gene ontology and KEGG databases used for grouping the leading GO terms included Gene Ontology Browser (https://www.informatics.jax.org), Gene Ontology and GO Annotations (https://www.ebi.ac.uk) and KEGG Pathway Database (https://www.genome.jp/kegg/pathway.html).

### 2.6 Quantitative real-time PCR (qPCR) assay

Samples (head kidney and skin) collected from representatives of 12 families (infested with adult or chalimus stages of lice at 10°C or 20°C) were included in qPCR analyses. Of these, representatives from four families were analyzed under both 10°C and 20°C conditions (i.e., F175, F229, F361, F419) and those of eight families were analyzed at either 10°C (F153, F154, F185, F265) or 20°C (F215, F292, F397, F415). For qPCR analyses, cluster of differentiation 83 (*cd83*), c-type lectin domain family 4 (*clec4*), collagen alpha-1(I) chain (*col1a1*), matrix metalloproteinase 9 (*mmp9*), neutrophil cytosolic factor 2 (*ncf2*), serum amyloid A5 (*saa5*), tissue inhibitor of metalloproteinase 2 (*timp2*) and thioredoxin b (*txnb*) were selected as genes of interest (GOIs) alongside three reference genes including eukaryotic translation initiation factor 3 subunit 6 (*eif3*), elongation factor 1 alpha (*ef1a*) and RNA polymerase I (*rpl1*) (Sutherland et al., 2014; Skugor et al., 2008; Todorčević et al., 2008; Leong et al., 2010; Braden et al., 2015; Braden et al., 2018; Chalmers et al., 2018; Carvalho et al., 2020; Xue et al., 2021). The selection of these genes was based on previous studies that demonstrated their relevance to host responses during sea lice infestation (Braden et al., 2015; Braden et al., 2018; Carvalho et al., 2020). The selected genes along with the primer sequences used in this study are listed in Supplementary Table S1.

A total of 1 µg of extracted RNA samples was used for synthesis of complementary DNA (cDNA, 20 µL reactions) using a commercially available kit (High Capacity cDNA Reverse Transcription Kit, Applied Biosystems, CA, United States) as per manufacturer’s instructions. The qPCR reactions were conducted on CFX96 Touch Real-Time PCR System (BioRad) in 12 µL reaction mixture comprised of 5 µL of Sso Advanced™ Universal SYBR^®^ Green Supermix (Bio-Rad), 0.5 µL of 10 mM forward and reverse primers, 4 µL of nuclease-free water and 2 µL of cDNA template using the cycling conditions: 95°C for 30 s, 95°C for 15 s, 60°C for 15 s, for 39 cycles, followed by a dissociation curve analysis ramping from 65°C to 95°C with continuous fluorescence detection every 0.5 s with a ramp rate of 0.5°C. qPCR reactions were performed in technical triplicates for each sample, alongside a calibrator pool (an equimolar mix of all cDNA samples for each tissue type), and two control reactions: a no-template control (NTC) and a no-reverse transcriptase control (no-RT). Gene expression levels for all genes of interest (GOIs) were normalized to the geometric mean of three housekeeping genes (*eif3*, *ef1a*, *rpl1*), incorporating gene-specific amplification efficiencies. Relative quantification (RQ) values were calculated using the comparative Ct (ΔΔCt) method, with the calibrator pool serving as the reference sample (Bustin et al., 2009). RQ values were then log_2_-transformed and statistically compared between groups infested with adult and chalimus stages of sea lice.

### 2.7 Statistical analysis

The normality of lice abundance and gene expression data was checked using the Shapiro-Wilk test. One-way analysis of variance (ANOVA), followed by Tukey’s *post hoc* test, was performed to assess significant differences in lice abundance among salmon families infested with either chalimus or adult stages of sea lice under each temperature condition. An independent samples *t*-test (or Mann-Whitney U test, where appropriate) was used to assess significant differences in gene expression levels between families infested with chalimus *versus* adult stages of lice. The same tests were also applied to compare lice abundance between temperature conditions for families infested with either chalimus or adult stages. A significance level of *p* < 0.05 was applied for all statistical comparisons. The qPCR and lice abundance data analyses were performed using GraphPad Prism version 10 (GraphPad Software Inc., United States).

## 3 Results

### 3.1 Differences in lice burden

**FIGURE 1:**
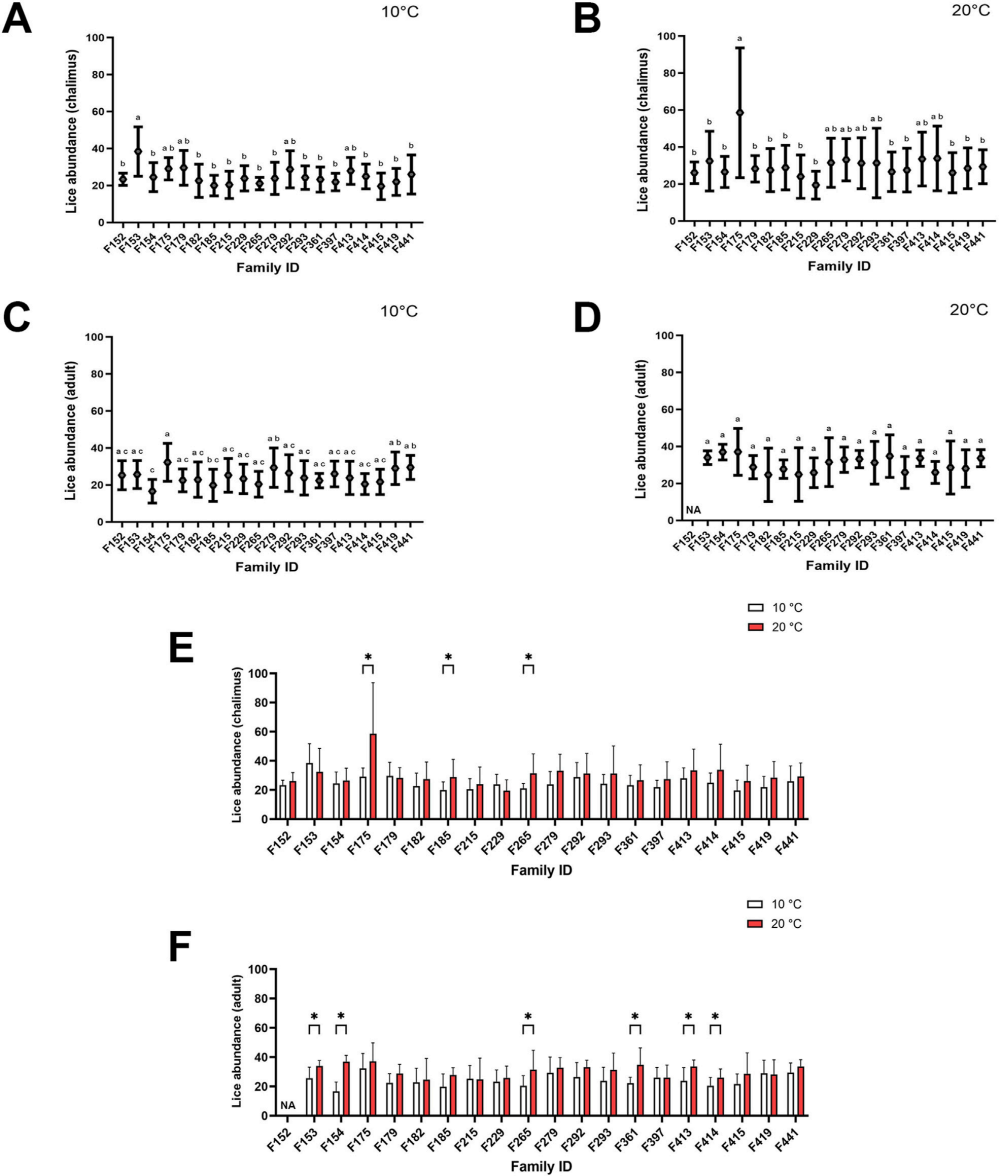
Average lice abundance (mean ± SD) in twenty salmon families infested with chalimus and adult stages of sea lice. Lice abundance was calculated for families infested with chalimus stages at 10°C **(A)** and 20°C **(B)**, and for families infested with adult stages at 10°C **(C)** and 20°C **(D)**. Average lice abundance was compared between temperature conditions for families infested with chalimus **(E)** and adult **(F)** lice stages. Different lowercase letters denote significant differences (*p* < 0.05) in lice abundance among families infested with chalimus or adult stages at each temperature **(A–D)**. Asterisks indicate significant differences (*p* < 0.05) in lice abundance based on within-family comparisons between the two temperature conditions following infestation with either chalimus or adult lice stages **(E,F)**. Representatives of five families (F175, F265, F292, F361 and F419) were included in RNA-seq analyses.

The mean lice abundance calculated for families infested by chalimus or adult stages of sea lice at either temperature (10°C and 20°C) has been summarized in Figures 1A–D. The mean lice abundance (calculated here for both chalimus and adult stages) for families included in RNA-seq analyses (F361, F175, F419, F265, F292) was respectively as follows: F361 (22.83 ± 5.52 at 10°C; 30.94 ± 11.57 at 20°C), F175 (30.70 ± 8.34 at 10°C; 45.38 ± 24.95 at 20°C), F419 (25.68 ± 8.71 at 10°C; 28.30 ± 10.27 at 20°C), F265 (20.74 ± 5.41 at 10°C; 31.53 ± 12.70 at 20°C), F292 (27.64 ± 9.80 at 10°C; 32.00 ± 10.94 at 20°C). The mean lice abundance was higher in most families infested at 20°C compared to 10°C, with significant differences observed in F175, F185 and F265 for chalimus-infested fish (Figure 1E), and in F153, F154, F265, F361, F413 and F414 for adult-infested fish (Figure 1F). Greater variability in lice abundance among families infested with chalimus stages was observed at the higher temperature condition (Figure 1E).

### 3.2 Transcriptomic responses

**FIGURE 2:**
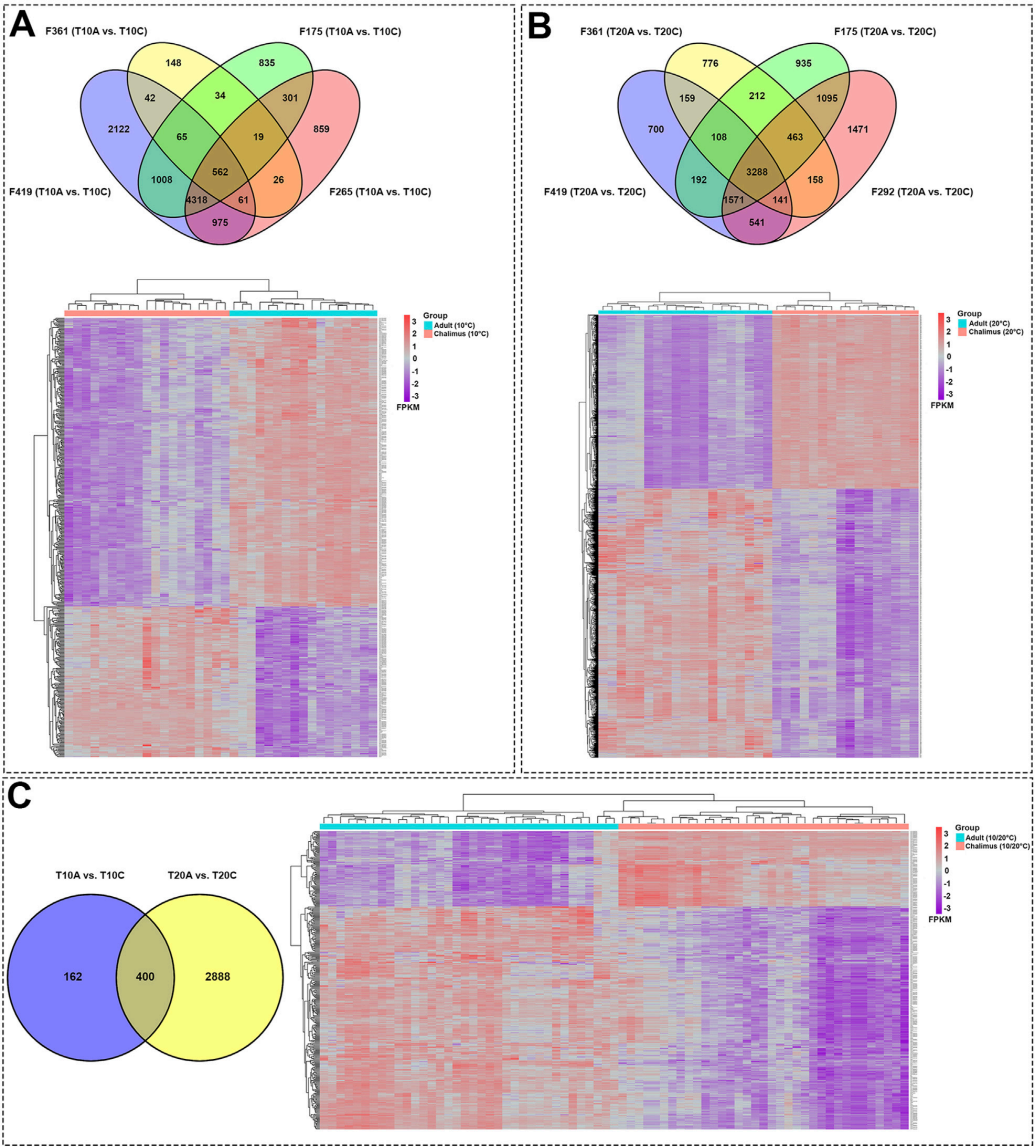
Skin transcriptome responses of Atlantic salmon infested with adult compared to chalimus stages of sea lice at normal and elevated temperature conditions. **(A)** The top Venn diagram shows the distribution of DEGs identified based on specific-family pairwise comparisons (F361, F175, F419, F265) in response to different infestation stages of lice at 10°C (T10A *vs*. T10C). The bottom heatmap illustrates the hierarchical clustering of shared DEGs (n = 562) among all families following infestation with different developmental stages of lice at 10°C. **(B)** The top Venn diagram represents the distribution of DEGs resulting from within-family pairwise comparisons (F361, F175, F419, F292) in response to different lice infestation stages at 20°C (T20A *vs*. T20C). The bottom heatmap displays the hierarchical clustering of shared DEGs (n = 3288) among all families following infestation with different developmental stages of lice at 20°C. **(C)** The left Venn diagram depicts the interaction of shared DEGs among all families infested with adult *versus* chalimus stages of lice at 10°C, in comparison to those infested at 20°C (T10A *vs*. T10C | T20A *vs*. T20C). The bottom heatmap presents the hierarchical clustering of shared DEGs (n = 400) across all families infested with adult *versus* chalimus lice stages at both 10°C and 20°C. Heatmaps were generated using FPKM-normalized gene expression values.

**FIGURE 3:**
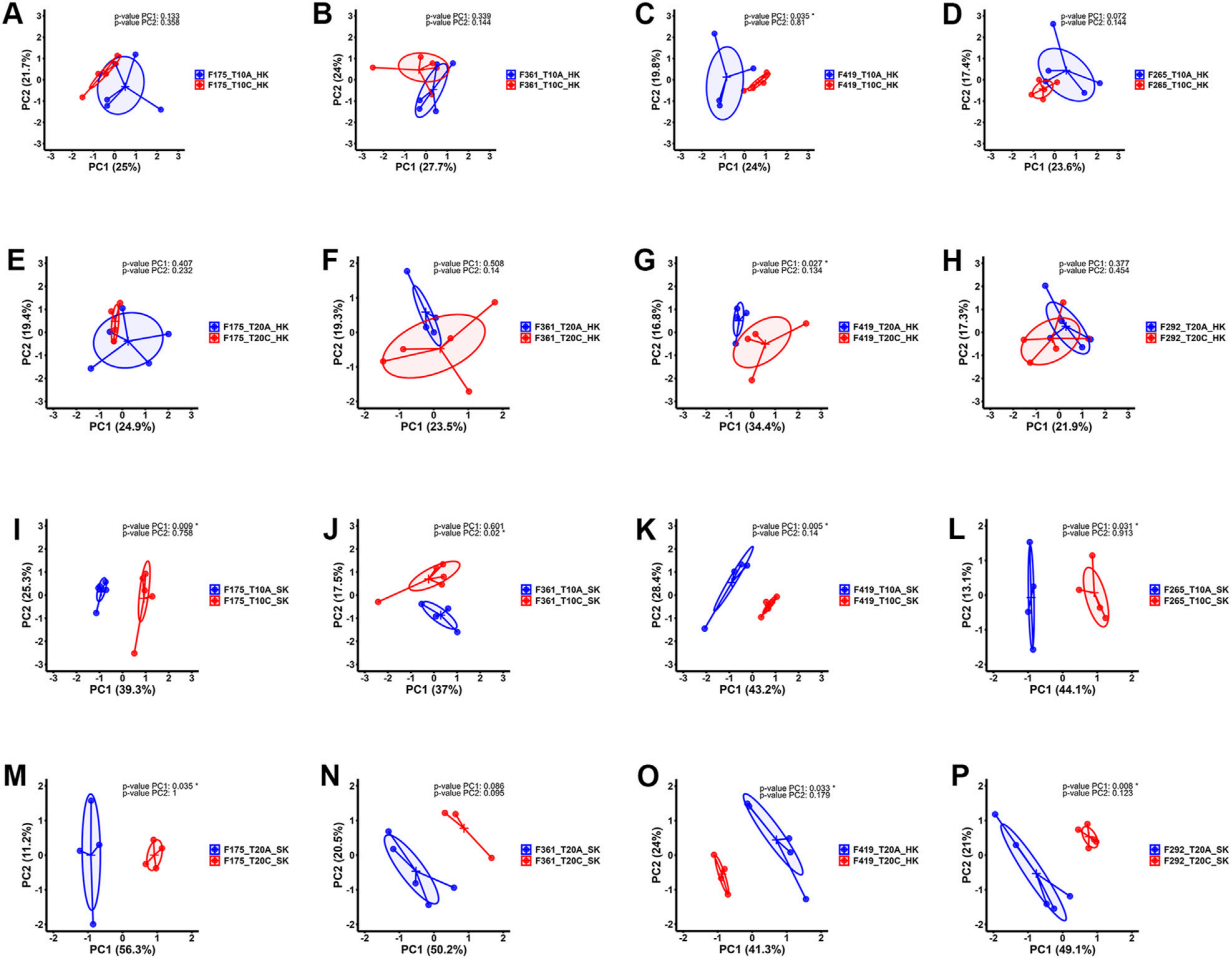
Principal component analysis (PCA) plots illustrating the expression data distribution of RNA-seq samples. **(A–D)** PCA plots based on the expression data comparing head kidney samples from families infested with adult (blue) *versus* chalimus (red) stages of sea lice at 10°C. **(E–H)** PCA plots based on the expression data related to comparisons between head kidney samples of families infested with adult (blue) compared to chalimus (red) stages of sea lice at 20°C. **(I–L)** PCA plots presenting the expression data comparing skin samples from families infested with adult (blue) *versus* chalimus (red) stages of sea lice at 10°C. **(M–P)** PCA plots illustrating the expression data from skin samples of families parasitized with adult (blue) *versus* chalimus (red) stages of sea lice at 20°C.

Pairwise comparisons within specific families (F361, F175, F419, F265, F292) infested with two different developmental stages of lice (adult *vs*. chalimus), which were separately carried out at two different temperatures (T10A *vs*. T10C and T20A *vs*. T20C, see Table 1), resulted in a remarkable number of DEGs in the skin (Supplementary Files S1–3) compared to the head kidney (Supplementary Files S4, 5). Venn diagrams generated using DEGs identified based on pairwise comparisons within families showed 562 and 3288 shared DEGs among all families at 10°C (T10A *vs*. T10C) and 20°C (T20A *vs*. T20C) in the skin, respectively (Figures 2A,B). There were 400 shared DEGs (299 consistently upregulated and 101 uniformly downregulated) among all families at both 10°C and 20°C in the skin (T10A *vs*. T10C | T20A *vs*. T20C). The heatmaps created using shared DEGs identified in the skin of fish infested at 10°C (n = 562) and 20°C (n = 3288), and those common at both temperatures (n = 400) resulted in complete clustering of their associated samples based on parasite infestation stage (Figure 2C). PCA conducted on the expression data associated with comparisons between infested families did not result in distinct segregation of head kidney samples in most comparisons (*p* > 0.05 for PC1 or PC2), suggesting inconsiderable differences between transcriptomic responses of fish infested with adult compared to chalimus stages of lice in this tissue (Figures 3A–H). On the other hand, PCA (Figures 3I–P) revealed distinct grouping (*p* < 0.05 for PC1 or PC2) of skin samples based on infestation stages of lice (T10A *vs*. T10C and T20A *vs*. T20C). The more frequent/significant genes identified based on family-level comparisons between infestation stages (expressed in the skin of most or all families) are listed in Supplementary File S6.

**TABLE 1:** Differentially expressed genes (DEGs) resulted from pairwise comparisons within specific salmon families parasitized with adult compared to chalimus stages of sea lice at a particular temperature for each tissue.

Pairwise comparisons	DEGs no. in skin			DEGs no. in head kidney		
	Up	Down	Total	Up	Down	Total
T10A *vs*. T10C						
F419	4096	5057	9153	324	78	402
F361	595	362	957	9	73	82
F175	2903	4239	7142	32	117	149
F265	3197	3924	7121	20	158	178
Shared DEGs			562			0
T20A *vs*. T20C						
F419	3500	3200	6700	256	58	314
F361	2721	2584	5305	18	12	30
F175	3912	3952	7864	39	39	78
F292	4362	4366	8728	129	142	271
Shared DEGs			3288			0
T20A *vs*. T20C | T20A *vs*. T20C						
Shared DEGs			400			0

Up: upregulation; down: downregulation.

### 3.3 GO/KEGG enrichment analyses of DEGs

**FIGURE 4:**
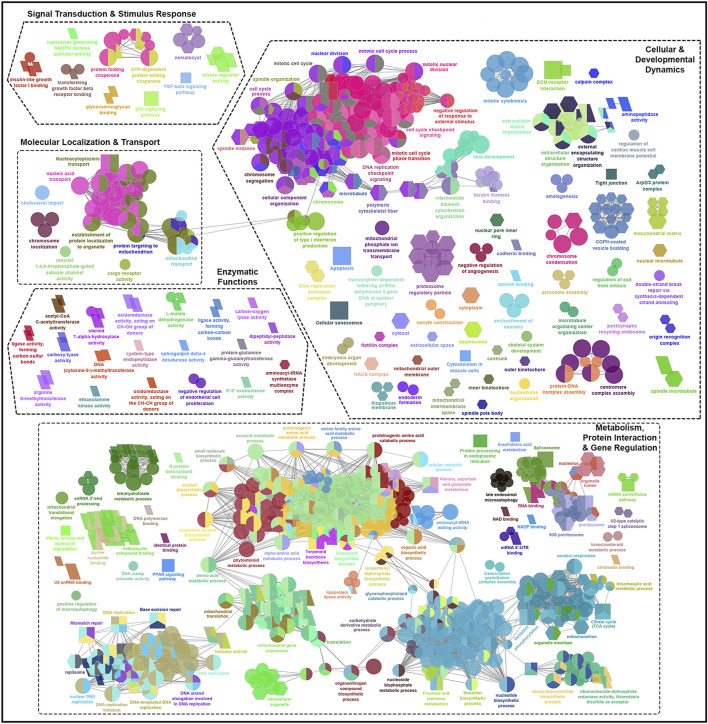
Functionally grouped network visualization of enriched GO terms and KEGG pathways based on infestation stage comparisons under elevated temperature condition. GO terms and KEGG pathways enriched based on shared DEGs identified in the skin of all families in response to infestation with adult *versus* chalimus stages of sea lice at 20°C (T20A *vs*. T20C). The leading GO terms and KEGG pathways were classified into 5 functional themes including metabolism, protein interaction and gene regulation (1), cellular and developmental dynamics (2), signal transduction and stimulus response (3), enzymatic functions (4), and molecular localization and transport (5). The GO domains and KEGG pathways are represented as follows: GO biological process (ellipse), GO cellular component (hexagon), GO molecular function (parallelogram), KEGG pathways (rectangle). Connections between GO terms and KEGG pathways represent shared genes among categories, grouped based on functional similarity using ClueGO’s kappa statistics (kappa score: 0.4). Only terms with Benjamini–Hochberg corrected *p*-values <0.05 were included.

**FIGURE 5:**
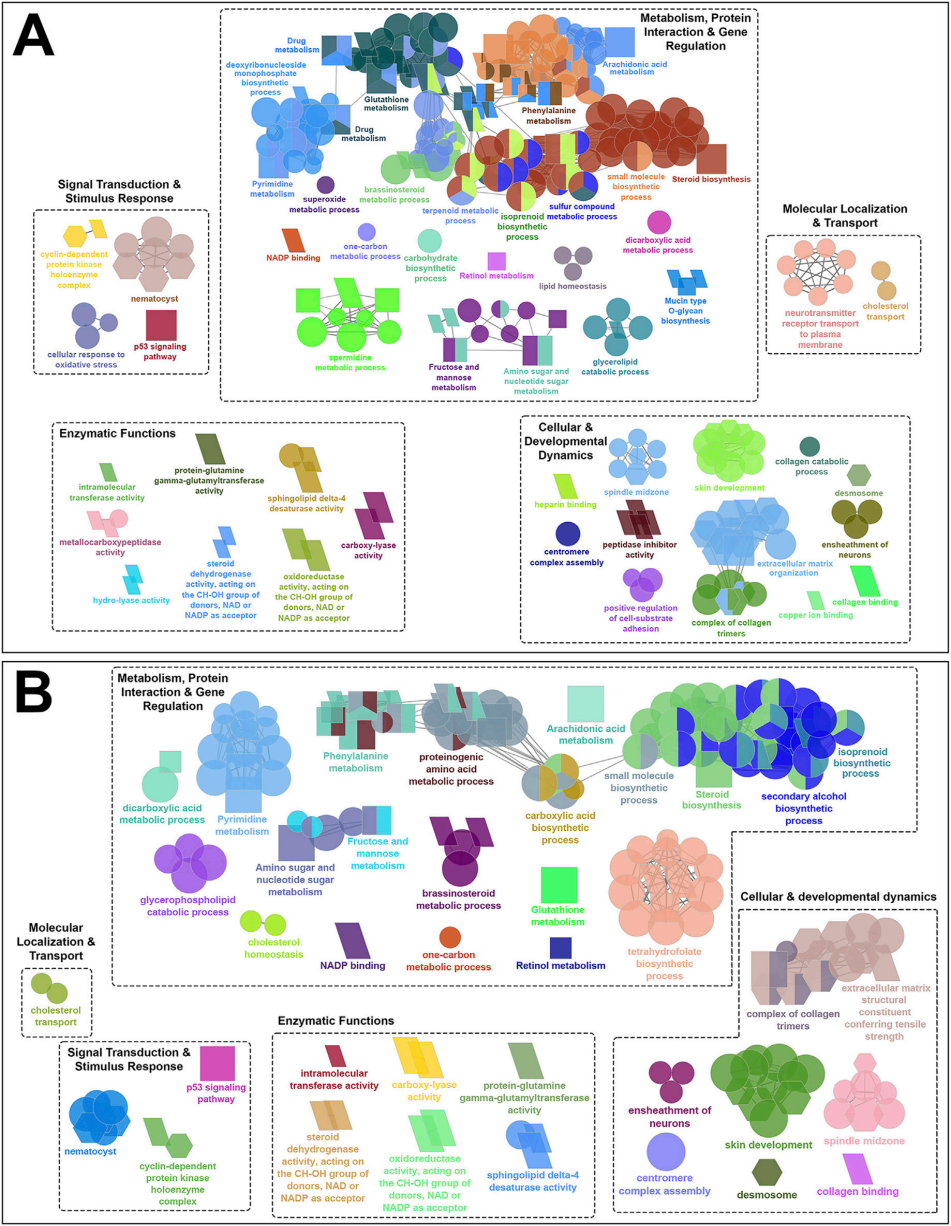
Functionally grouped network visualization of enriched GO terms and KEGG pathways based on infestation stage comparisons under different temperature conditions. **(A)** GO terms and KEGG pathways enriched based on shared DEGs identified in the skin of all families following infestation with adult compared to chalimus stages of sea lice at 10°C (T10A *vs*. T10C). **(B)** GO terms and KEGG pathways enriched based on shared DEGs across all families infested with adult *versus* chalimus lice stages at both 10°C and 20°C (T10A *vs*. T10C | T20A *vs*. T20C). The leading GO terms and KEGG pathways were classified into 5 functional themes including metabolism, protein interaction and gene regulation (1), cellular and developmental dynamics (2), signal transduction and stimulus response (3), enzymatic functions (4), and molecular localization and transport (5). The GO domains and KEGG pathways are represented as follows: GO biological process (ellipse), GO cellular component (hexagon), GO molecular function (parallelogram), KEGG pathways (rectangle). Connections between GO terms and KEGG pathways represent shared genes among categories, grouped based on functional similarity using ClueGO’s kappa statistics (kappa score: 0.4). Only terms with Benjamini–Hochberg corrected *p*-values <0.05 were included.

The GO terms and KEGG pathways associated with shared skin DEGs across all families comparing adult and chalimus stages at each temperature and their interaction (T10A *vs*. T10C, T20A *vs*. T20C and T10A *vs*. T10C | T20A *vs*. T20C) are provided in Supplementary Files S7–9. The leading GO terms and KEGG pathways were classified into five functional themes (Figures 4, 5A,B). Overall, the theme ‘metabolism, protein interaction and gene regulation’ represented higher number of GO terms and KEGG pathways compared to other functional themes. The GO and KEGG enrichment analyses revealed several terms/pathways involved in metabolism of sugars (fructose, mannose), lipids (arachidonic acid, cholesterol, steroid/brassinosteroid, sphingolipid, glycerophospholipids), proteins (proteinogenic amino acids, phenylalanine) and/or other organic compounds (secondary alcohols, small molecules, isoprenoids, pyrimidine, one-carbon molecules, carboxylic acid, dicarboxylic acid, tetrahydrofolic acid, retinol) in the skin of fish infested with adult *versus* chalimus stages. Notably, metabolism-related GO terms and KEGG pathways exhibited greater interconnectedness based on infestation stage comparisons, particularly under elevated temperature (Figure 4).

Our findings suggest enhanced metabolic demands and stress-induced metabolic reprogramming in infested fish maintained at 20°C compared to those held at 10°C (Figure 4). This is supported by the enrichment of several over-represented GO terms and KEGG pathways related to DNA repair and processing (e.g., “chromatin binding” [GO:0003682], “DNA replication” [KEGG:03030]), RNA processing (e.g., “RNA binding” [GO:0016071], “spliceosome” [GO:0000398]), and protein-protein interactions (e.g., “translation” [GO:0090079], “protein processing in endoplasmic reticulum” [KEGG:04141]). Furthermore, broader metabolic processes/pathways involving a greater number of genes were enriched, including “carbohydrate derivative metabolic process” (GO:1901135), “small molecule biosynthetic process” (GO:0032787), “organonitrogen compound biosynthetic process” (GO:0006412) and “PPAR signaling pathway” (KEGG:03320). Examples of GO terms and KEGG pathways enriched based on shared DEGs related to T10A *vs*. T10C (Figure 5A) included “carbohydrate biosynthetic process” (GO:0016051), “terpenoid metabolic process” (GO:0042445) and “mucin type O-glycan biosynthesis” (KEGG:00512), while terms/pathways associated with T10A *vs*. T10C | T20A *vs*. T20C (Figure 5B) were represented by “carboxylic acid biosynthetic process” (GO:0016053), “glycerophospholipid catabolic process” (GO:0046434), “glutathione metabolism” (KEGG:00480) and “arachidonic acid metabolism” (KEGG:00590).

The infestation stage-related leading GO terms and/or KEGG pathways classified under “cellular and developmental dynamics” were more frequent and diverse at elevated temperature condition (Figure 4). For instance, there was a notable enrichment in terms associated with cell cycle control (e.g., “cell cycle process” [GO:0022402]), cellular and structural organization (e.g., “cytoplasm” [GO:0005737], “cellular component organization” [GO:0016043], “extracellular structure organization” [GO:0030020]), and organelle function and assembly (e.g., “mitochondrial outer membrane” [GO:0005741]). Moreover, the enrichment of KEGG pathways such as “apoptosis” (KEGG:04210), “cellular senescence” (KEGG:04218) and “tight junction” (KEGG:04530), with predominantly upregulated associated genes, likely reflects an intensified host effort to counteract parasite-induced damage under higher temperature stress. Comparatively, the narrower enrichment of GO terms and KEGG pathways related to localized tissue remodeling at 10°C (Figure 5A), such as “ensheathment of neurons” (GO:0007272), “skin development” (GO:0008544), “extracellular matrix organization” (KEGG:04512), suggests a more controlled developmental and structural response at this thermal condition.

Compared to the previously described functional themes, GO terms and/or KEGG pathways associated with ‘signal transduction and stimulus response’, ‘enzymatic functions’ and ‘molecular localization and transport’ were less abundant and exhibited lower levels of functional connectivity (Figure 4; Figures 5A,B). However, within these themes, the associated GO terms and/or KEGG pathways also contained a broader and more frequently occurring set of genes under elevated temperature conditions. Among the GO terms classified under ‘signal transduction and stimulus response’, “protein-glutamine gamma-glutamyltransferase activity” (GO:0003810), “carboxy-lyase activity” (GO:0016830), oxidoreductase activity (GO:0016614) and “sphingolipid delta-4 desaturase activity” (GO:0016717) were consistently enriched at both 10°C and 20°C (Figure 5B). This pattern suggests a core enzymatic response of Atlantic salmon to lice infestation (adult *vs*. chalimus) regardless of temperature, likely contributing to tissue remodeling and repair (via transglutaminase activity), lipid metabolism and membrane remodeling (via sphingolipid desaturase), and redox homeostasis (via oxidoreductases).

The leading GO terms and KEGG pathways linked to the functional theme ‘signal transduction and stimulus response’ that were enriched under both temperature conditions (Figure 5B) included those associated with pore formation (“nematocyst” [GO:0044218]) and with stress signaling and cell cycle regulation response (“p53 signaling pathway” [KEGG:04115], “cyclin-dependent protein kinase holoenzyme complex” [GO:0016538]). A notable expansion of GO terms and KEGG pathways related to growth factor signaling (e.g., “insulin-like growth factor I binding” [GO:0005520], “TGF-beta signaling pathway” [KEGG:04350]), kinase regulation (“kinase regulator activity” [GO:0019207]), and chaperone-mediated folding (e.g., “protein folding chaperone” [GO:0140662]) indicates the enhanced signal transduction complexity at elevated temperature (Figure 4). Likewise, a broader range of GO terms and KEGG pathways under the theme ‘molecular localization and transport’ were enriched at 20°C (Figure 4), including those associated with organelle-specific transport (e.g., “mitochondrial transport” [GO:0006839]), macromolecule localization (e.g., “chromosome localization” [GO:0050000], “nucleic acid transport” [KEGG:03013], “nucleocytoplasmic transport” [KEGG:03013]) and trafficking mechanisms (e.g., cargo receptor activity [GO:0038024], establishment of protein localization to organelle [GO:0006839]). These findings suggest that compartmental communication, intracellular trafficking, and molecular localization were impacted under enhanced temperature conditions.

The infestation stage-associated leading GO terms and KEGG pathways enriched based on shared DEGs identified in the skin of all families at both thermal conditions along with their corresponding up- and downregulated DEGs have been summarized in Table 2 (see Supplementary Table S2 for the complete list). *tgm2*, *tgm3*, *emx1*, *camp*, *tubb2a*, *ddit4l*, *il1b*, *iff6*, *mslna*, *mal*, *casp3b*, *lbh*, *krt8*, *krt13* and cell surface glycoprotein 1 (unavailable symbol) were among shared DEGs with high transcript levels that were expressed in all families infested by adult compared to chalimus stages of lice at both temperatures. By comparison, *entpd5a*, *fndc1*, *chad*, *ccdc3b*, *prss23*, *ncana*, *col10a1a*, *col1a2*, *col16a1* and *col1a1* were among shared DEGs with lowest expressions in all families parasitized with adults compared to chalimus stages at both thermal conditions.

**Table 2:** Selected leading GO terms and KEGG pathways enriched based on shared DEGs associated with pairwise comparisons within specific salmon families (F419, F361, F175, F265, F292) infested with adult compared to chalimus stages of lice at 10°C and 20°C in the skin (see Supplementary Table S2 for the complete list of enriched leading GO terms).

GO description	GO/KEGG ID	Gene ID	Gene symbol	Gene description	Fold-change (log_2_FC)							
					T10A *vs*. T10C				T20A *vs*. T20C			
					F419	F361	F175	F265	F419	F361	F175	F292
Protein-glutamine gamma-glutamyltransferase activity	GO:0003810	LOC106597223	*tgm2*	Protein-glutamine gamma-glutamyltransferase 2	8.85	7.48	8.12	8.53	8.47	7.83	8.19	9.69
		*tgm2*	*tgm2*	Transglutaminase 2, C polypeptide	4.43	2.36	5.00	3.10	3.85	2.64	2.13	2.09
		LOC106584295	*tgm1*	Protein-glutamine gamma-glutamyltransferase K	4.10	1.94	1.25	2.49	2.80	1.57	2.77	3.83
		LOC106583691	*tgm2*	Protein-glutamine gamma-glutamyltransferase 2	2.14	1.34	1.51	1.92	2.29	2.16	2.93	2.98
Glutathione metabolism	KEGG:00480	LOC106611136	*odc1*	Ornithine decarboxylase 1	3.79	1.48	2.23	4.00	3.52	3.26	3.11	3.67
		LOC106607664	*rrm2*	Ribonucleoside-diphosphate reductase subunit M2	3.64	1.73	3.57	3.02	3.56	2.77	3.25	3.64
		*pgd*	*pgd*	Phosphogluconate dehydrogenase	2.99	1.43	2.91	2.39	2.72	1.76	2.95	3.08
		LOC106586335	*idh1*	Isocitrate dehydrogenase [NADP] cytoplasmic	2.88	1.46	2.69	2.87	2.82	2.48	2.98	3.28
		*rir2*	*rrm2*	Ribonucleoside-diphosphate reductase subunit M2	2.77	1.56	3.06	2.11	2.77	2.10	2.36	2.63
		*gsr*	*gsr*	Glutathione reductase	2.50	1.77	2.71	2.26	2.72	2.01	2.70	2.92
		*chac1*	*chac1*	ChaC, cation transport regulator homolog 1 (*E. coli*)	2.00	1.36	1.93	2.42	3.10	2.16	2.26	3.34
p53 signaling pathway	KEGG:04115	*casp3b*	*casp3b*	Caspase 3, apoptosis-related cysteine peptidase b	5.20	3.66	4.29	4.49	5.43	3.76	4.18	5.72
		LOC106607664	*rrm2*	Ribonucleoside-diphosphate reductase subunit M2	3.64	1.73	3.57	3.02	3.56	2.77	3.25	3.64
		LOC100195405	*ccne2*	Cyclin E2	3.59	1.48	3.82	3.42	3.66	2.96	3.70	3.68
		LOC106566708	*ccnb1*	G2/mitotic-specific Cyclin-B1	3.54	1.30	3.63	3.06	3.90	3.61	3.41	3.98
		*ccnb1*	*ccnb1*	Cyclin B1	3.44	1.50	3.74	2.93	3.62	3.51	3.23	3.74
		LOC106607826	*cdk1*	Cyclin-dependent kinase 1	2.96	1.30	2.75	2.13	3.30	2.93	2.66	3.18
		*rir2*	*rrm2*	Ribonucleoside-diphosphate reductase subunit M2	2.77	1.56	3.06	2.11	2.77	2.10	2.36	2.63
		LOC106586251	*cycs*	Cytochrome c	2.77	2.13	1.69	2.79	2.86	1.74	2.40	3.13
		LOC100196698	*perp*	PERP, TP53 apoptosis effector	2.47	1.35	1.79	1.97	2.16	1.79	2.32	2.58
		*cdc2*	*cdk1*	Cell division control protein 2 homolog	2.12	1.28	2.42	1.68	2.64	2.65	1.81	2.34
		*casp3*	*casp3*	Caspase-3	1.88	1.66	1.88	1.73	1.48	1.32	1.41	1.61
Amino sugar and nucleotide sugar metabolism	KEGG:00051; KEGG:00520; GO:0051156; GO:0009225; GO:0009226	LOC106589825	*hk1*	Hexokinase-1	3.07	1.53	1.92	2.01	2.55	1.80	2.49	2.94
		*pgd*	*pgd*	Phosphogluconate dehydrogenase	2.99	1.43	2.91	2.39	2.72	1.76	2.95	3.08
		*tigara*	*tigara*	TP53 induced glycolysis regulatory phosphatase a	2.69	1.82	2.86	2.38	2.92	2.57	2.90	2.90
		*gmds*	*gmds*	GDP-mannose 4,6-dehydratase	2.63	1.81	2.66	2.26	2.46	2.26	2.48	2.41
		*nansa*	*nansa*	N-acetylneuraminic acid synthase a	2.36	1.36	1.90	1.71	1.78	1.56	1.82	2.03
		*pgm3*	*pgm3*	Phosphoglucomutase 3	2.21	1.40	1.88	1.72	1.83	1.31	1.52	1.64
		*gmppb*	*gmppb*	GDP-mannose pyrophosphorylase B	2.12	1.27	1.77	1.71	1.52	1.28	1.56	1.83
		*gfpt1*	*gfpt1*	Glutamine-fructose-6-phosphate transaminase 1	2.09	1.55	2.07	1.88	1.66	1.38	1.83	1.88
		LOC106610806	*gale*	UDP-glucose 4-epimerase	2.08	1.49	1.88	1.71	1.58	1.39	1.72	1.85
Skin development	GO:0008544; GO:0043588; GO:0030855; GO:0045104; GO:0045109; GO:0009913; GO:0031424; GO:0030216; GO:0045111; GO:0005882; GO:0045095	LOC106600778	*iff6*	Cell wall protein IFF6	5.52	5.40	6.40	6.56	4.30	7.85	5.74	7.15
		LOC106565226	*krt8*	Keratin, type II cytoskeletal 8	5.26	3.02	4.60	4.27	5.84	5.23	5.96	6.83
		*casp3b*	*casp3b*	Caspase 3, apoptosis-related cysteine peptidase b	5.20	3.66	4.29	4.49	5.43	3.76	4.18	5.72
		LOC106600814	*krt13*	Keratin, type I cytoskeletal 13	4.48	5.50	5.53	5.95	5.14	7.82	7.43	8.67
		LOC106584295	*tgm1*	Protein-glutamine gamma-glutamyltransferase K	4.10	1.94	1.25	2.49	2.80	1.57	2.77	3.83
		LOC106572756	*krt18*	Keratin, type I cytoskeletal 18	3.86	2.05	2.97	1.99	4.59	2.75	3.70	3.66
		LOC106564958	*krt13*	Keratin, type I cytoskeletal 13	3.30	2.70	3.97	2.89	4.47	3.14	3.88	4.18
		LOC106600816	*krt13*	Keratin, type I cytoskeletal 13	2.99	2.79	4.07	3.17	4.72	4.03	4.45	5.17
		*eppk1*	*eppk1*	Epiplakin 1	2.95	1.82	2.22	1.44	3.75	2.32	3.01	3.44
		LOC106590260	*pck2*	Phosphoenolpyruvate carboxykinase 2 (mitochondrial)	2.44	1.34	2.96	2.95	2.86	2.96	2.81	3.38
		*casp3*	*casp3*	Caspase-3	1.88	1.66	1.88	1.73	1.48	1.32	1.41	1.61
		*scel*	*scel*	Sciellin	1.85	1.46	1.37	1.67	1.47	1.29	1.60	2.03
		LOC106583605	*krt8*	Keratin, type II cytoskeletal 8	−3.37	−1.39	−3.06	−2.77	−1.76	−1.98	−2.15	−2.88
		*col1a1a*	*col1a1a*	Collagen, type I, alpha 1a	−5.09	−1.83	−4.19	−3.36	−1.87	−2.31	−2.54	−3.83
Extracellular matrix structural constituent conferring tensile strength	KEGG:04512; GO:0030020; GO:0043062; GO:0045229; GO:0098644; GO:0030198; GO:0098651; GO:0005604; GO:0098643; GO:0098645; GO:0098642; GO:0005583; GO:0005587	LOC106568399	*itga2*	Integrin alpha-2	4.34	2.59	4.06	3.32	3.59	2.57	3.97	4.45
		*mmp9*	*mmp9*	Matrix metalloproteinase 9	4.02	2.40	3.93	2.09	3.86	1.28	3.74	4.97
		LOC106579812	*col27a1b*	Collagen alpha-1 (XXVII) chain B	−1.43	−1.36	−2.04	−1.70	−1.59	−1.40	−1.73	−2.02
		LOC106583145	*col7a1*	Collagen alpha-1(VII) chain	−2.08	−1.27	−1.78	−2.18	−2.95	−1.72	−2.45	−3.87
		LOC106567192	*mmp19*	Matrix metalloproteinase-19	−2.47	−1.56	−3.30	−2.36	−1.51	−2.15	−2.26	−2.42
		LOC106569953	*col11a1*	Collagen alpha-1 (XI) chain	−2.49	−1.87	−2.88	−3.42	−3.53	−1.45	−2.92	−3.72
		LOC106583712	*col2a1*	Collagen alpha-1(II) chain	−2.66	−1.67	−2.66	−1.60	−1.45	−1.54	−1.29	−2.20
		*adamts2a*	*adamts2a*	ADAM metallopeptidase with thrombospondin type 1 motif, 2a	−2.77	−1.29	−2.34	−2.65	−1.87	−2.04	−2.13	−3.45
		LOC106562962	*col5a1*	Collagen alpha-1(V) chain	−3.14	−1.31	−3.30	−2.59	−1.60	−1.83	−1.88	−2.44
		LOC106562591	*adamts17*	A disintegrin and metalloproteinase with thrombospondin motifs 17	−3.77	−1.80	−3.40	−2.88	−3.09	−1.92	−2.10	−4.34
		LOC106586061	*col6a3*	Collagen alpha-3(VI) chain	−3.77	−1.44	−3.16	−2.98	−1.91	−2.05	−2.22	−3.01
		LOC106563762	*adamts2*	A disintegrin and metalloproteinase with thrombospondin motifs 2	−3.82	−1.51	−3.84	−3.29	−1.96	−1.77	−2.49	−3.06
		LOC106586705	*abi3bp*	Target of Nesh-SH3	−4.05	−1.45	−3.45	−3.71	−2.19	−2.02	−2.86	−3.63
		LOC106592099	*col11a1*	Collagen alpha-1 (XI) chain	−4.15	−1.36	−3.79	−3.55	−5.11	−3.04	−3.91	−5.34
		*ccdc80l2*	*ccdc80l2*	Coiled-coil domain containing 80 like 2	−4.18	−1.37	−3.50	−3.22	−1.92	−1.66	−2.76	−3.54
		LOC106612255	*sparc*	SPARC	−4.18	−1.28	−3.58	−3.18	−1.64	−1.93	−2.15	−3.11
		LOC106604192	*sparc*	SPARC	−4.40	−1.48	−3.80	−3.21	−1.80	−2.38	−2.46	−3.08
		LOC106588939	*col1a2*	Collagen alpha-2(I) chain	−4.51	−1.71	−3.81	−3.05	−1.60	−2.12	−2.23	−3.39
		LOC106600852	*col1a1*	Collagen alpha-1(I) chain	−4.73	−1.66	−4.01	−3.19	−1.68	−2.29	−2.40	−3.51
		LOC106610502	*col1a1*	Collagen alpha-1(I) chain	−4.77	−1.84	−4.07	−3.41	−1.96	−2.59	−2.44	−3.90
		*col2a1b*	*col2a1b*	Collagen, type II, alpha 1b	−4.81	−1.67	−5.16	−4.20	−1.96	−1.91	−3.22	−3.43
		*col1a1b*	*col1a1b*	Collagen, type I, alpha 1b	−5.03	−1.76	−4.14	−3.35	−2.26	−2.78	−3.16	−4.77
		*col1a1a*	*col1a1a*	Collagen, type I, alpha 1a	−5.09	−1.83	−4.19	−3.36	−1.87	−2.31	−2.54	−3.83
		LOC106570545	*col16a1*	Collagen alpha-1 (XVI) chain	−5.10	−1.49	−4.37	−3.98	−2.25	−1.77	−2.65	−3.56
		LOC106570460	*col1a2*	Collagen alpha-2(I) chain	−5.10	−1.75	−4.13	−3.41	−1.90	−2.26	−2.45	−3.83
		LOC106589269	*chad*	Chondroadherin	−5.22	−1.44	−3.27	−4.19	−2.87	−2.15	−4.20	−5.22

In the head kidney, contrary to skin, there were no shared DEGs among families infested with different developmental stages of lice. However, certain within-family comparisons were found to be associated with GO terms involved in detoxification (e.g., “peroxidase activity” and “hydrogen peroxide catabolic process”), heat shock response (“ATP-dependent protein folding chaperone” and “response to heat”), transporter activity (“bicarbonate transmembrane transporter activity” and “ammonium transmembrane transporter activity”) and immune response (“defense response to virus” and “lymphocyte differentiation”) (Supplementary Files S10, 11).

### 3.4 qPCR analysis of selected genes

**FIGURE 6:**
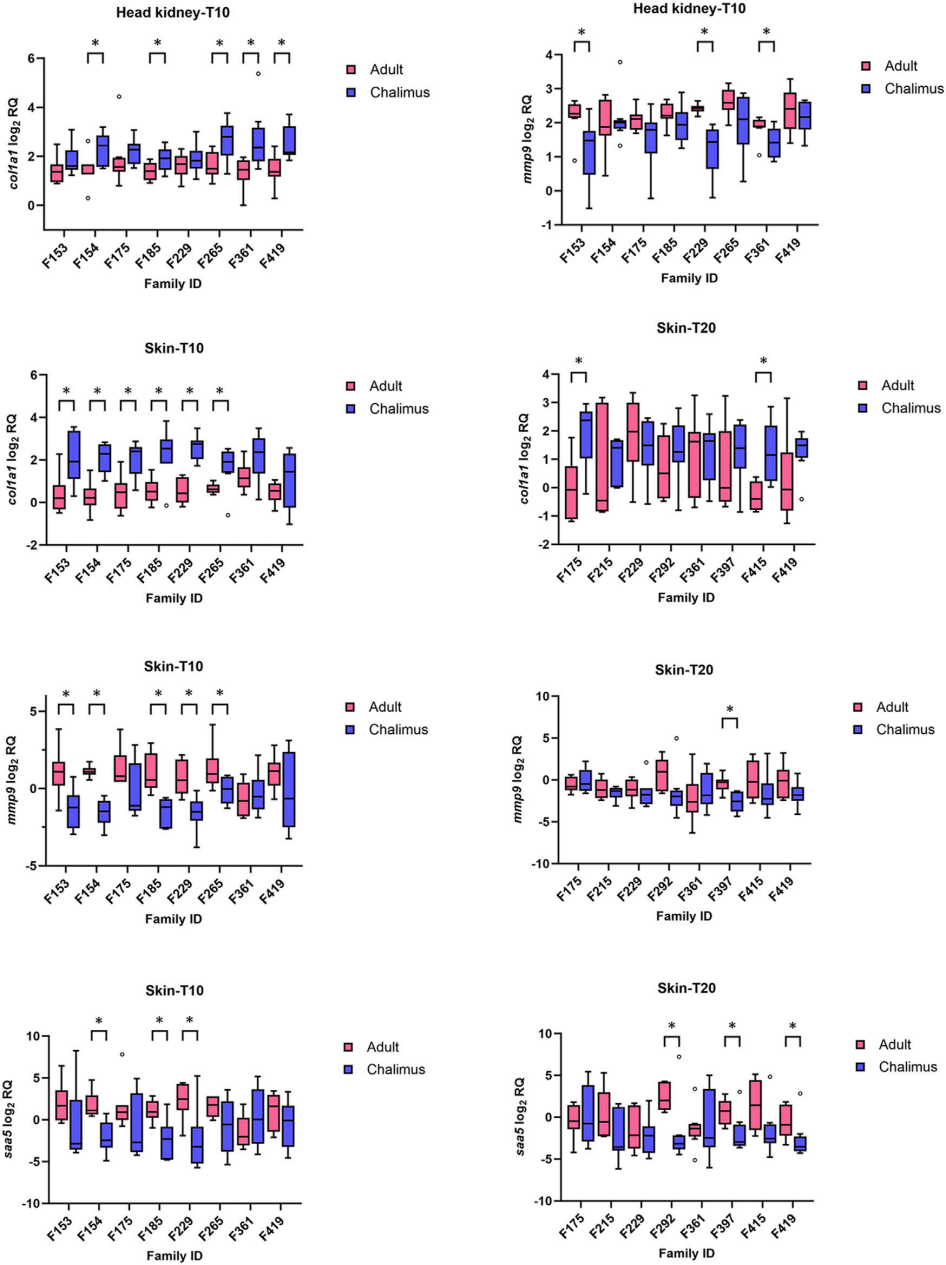
qPCR results for selected genes (*col1a1*, *mmp9* and *saa5*) with significant alterations in the skin and/or head kidney of different families infested by adult compared to chalimus stages of sea lice at 10°C (T10) or 20 (T20) °C. Data are presented as box plots with median and Tukey whiskers (fences). Significant differences (*p* < 0.05) in gene expression responses between infestation stages within each family are indicated by asterisks. qPCR results for all genes of interest (GOIs) tested in this study are presented in Supplementary Figures S1–S4.

Multiple samples from different families (n = 8 at each temperature [10 or 20°C] for head kidney [HK] and skin [SK]; HK-T10, HK-T20; SK-T10, SK-T20) were included in qPCR analyses. Of the eight GOIs (*cd83*, *clec4*, *col1a1*, *mmp9*, *ncf2*, *saa5*, *timp2* and *txnb*) selected for qPCR analyses, *col1a1* and *mmp9* revealed the most significant changes compared to other genes (Figure 6), particularly in fish infested at 10°C (see below). qPCR results for all GOIs analyzed in this study are presented in Supplementary Figures S1–S4. The expression of *col1a1* decreased in the skin and head kidney of all families at 10°C, with significant changes (*p* < 0.05) identified in F153, F154, F175, F185, F229 and F265 in SK-10, and F154, F185, F265, F361 and F419 in HK-10. Based on RNA-seq analyses, the transcript level of this gene (LOC106610502) was reduced in the skin of all families at both temperatures (F419, F175, F361, F265 and/or F292 in SK-10/20), but its lowered expression was detected in the head kidney of few families (F265 in HK-10 and F292 in HK-20). On the other hand, the mRNA level of *mmp9* increased in the skin of almost all families (except F361) at both temperatures, although these changes were significant (*p* < 0.05) in F153, F154, F185, F229 and F265 in SK-10, and F397 in SK-20 (Figure 6). RNA-seq analyses showed enhanced expression of *mmp9* in the skin of all families at both temperatures (F419, F175, F361, F265 and/or F292 in SK-10/20), with decreased upregulation in F361 compared to other families at both temperatures. qPCR results revealed increased expression of *mmp9* in the head kidney of certain families only at 10°C (F153, F229 and F361), but this gene was not differentially expressed at either temperature based on RNA-seq analyses. Supplementary Figures S1–S4.

The expression of *cd83* was mostly reduced in both the skin and head kidney of families infested by adult compared to chalimus at 10°C (Supplementary Figures S1, S3), although these changes were significant (*p* < 0.05) in F175 and F265 in HK-T10, and F185, F361 and F419 in SK-T10 (*p* < 0.05). The transcript level of *cd83* was unaffected in the skin and head kidney of most families at 20°C, and RNA-seq data showed no modulation of this gene in either tissue. The expression level of *clec4* was not impacted in the skin and head kidney of most families at both temperatures (Supplementary Figures S1–S4). According to RNA-seq data, different members of *clec4* family had mostly reduced expression in the skin of adult-compared to chalimus-infested families at both temperatures. RNA-seq analysis showed no modulation of *ncf2* in either tissue, although qPCR data indicated inconsistent modulation of this gene in a few families. Both RNA-seq and qPCR data did not show significant expression of *txnb*, *timp2* and *saa5* in the head kidney at both thermal conditions (Supplementary Figures S1, S2). Based on qPCR results, the transcript level of *txnb* increased in the skin of all families at 10°C, with significant changes (*p* < 0.05) in F154, F229 and F265. RNA-seq analysis revealed enhanced expression of this gene in some/all families at both temperatures. The expression of *timp2* was significantly (*p* < 0.05) higher in the skin of all families parasitized with adult compared to chalimus stages of lice in RNA-seq analysis. Based on qPCR data, increased expression of this gene was observed in the majority of families, although with significant alteration only in F154 and F229 at 10°C (Supplementary Figure S3). RNA-seq analysis revealed increased transcript levels of *saa5* in most families at both thermal profiles (Figure 6). Consistently with these findings, qPCR results indicated the enhanced expression of *saa5* in the skin of all families (except F361), where significant changes (*p* < 0.05) were observed in F154, F185 and F229 in SK-T10, and F292, F397 and F419 in SK-T20.

## 4 Discussion

This is the first comprehensive family-based transcriptome study in Atlantic salmon in response to infestation with different developmental stages of *L. salmonis*. Beyond its relevance to salmon aquaculture, this study provides broader insights into the molecular mechanisms underlying host-parasite interactions in lower vertebrates. The identification of host responses to distinct developmental stages of sea lice enhances our understanding of how parasites modulate host immunity at different phases of infestation. Moreover, these findings may inform the development of targeted strategies for breeding parasite-resistant fish, improving fish welfare, and enhancing the sustainability of aquaculture systems globally. Based on within-family pairwise comparisons (T10A *vs*. T10C, T20A *vs*. T20C), a large number of genes (e.g., *muc2*, *muc5ac*, *litaf*, *krt13*, *ccl20*, *lcn*, *cd22*, *map4k4*, *pbx1*, *trim16*) were represented by paralogs showing either similar or contrasting transcriptional patterns. This observation aligns with previous findings of transcriptional divergence among duplicated genes in Atlantic salmon and may reflect functional specialization following whole-genome duplication events (Macqueen and Johnston, 2014; Lien et al., 2016; Umasuthan et al., 2020; Ignatz et al., 2024). The GO and KEGG enrichment analyses revealed terms/pathways represented by DEGs (nearly all upregulated) encoding a diverse range of enzymes/biomolecules involved in metabolism of various organic substances in the skin of fish infested with adult *versus* chalimus stages. These metabolic shifts facilitate the mobilization of energy substrates/stores to cope with the stress caused during infestation. In addition, the enhanced expression of several genes directly or indirectly associated with organismal stress response (*ercc6*, *xrcc5*, *xrcc6*, *brca1*, *lepb2*, *ddb1*, *gadd45a*, *hspd1*, *hspa14*, *hsp90b1*, *cct8*, *cct6a*, *hif1ab*, *cirbp*, *hspa8* [*hsc70*], *ube2v1*, *ube2n*, *sumo2*) suggested a response to the more severe skin damage induced by adult forms compared to chalimus stages. These changes are consistent with elevated plasma cortisol levels and increased epithelial cell apoptosis and necrosis observed following infestation with advanced stages of sea lice (Johnson and Fast, 2004; Nolan et al., 1999).

The transcriptomic changes associated with infestation with adult *versus* chalimus stages of lice were remarkable in the skin. This variation was not visible in the head kidney samples from the same salmon between the two different stages. In the head kidney, no shared DEGs were identified between all families at either 10 or 20°C, and GO analyses revealed a limited number of GO terms. Based on within-family pairwise comparisons, the most frequent DEGs in the head kidney of fish infested with adult *versus* chalimus at both temperatures included *fcrl5*, *cat*, *hbb*, *hbb1* and *hba4* (downregulated). On the other hand, a total of 384 annotated genes (286 upregulated and 97 downregulated) were identified to be differentially expressed in the skin of Atlantic salmon infested with adult *versus* chalimus stages of sea lice at both 10°C and 20°C. Based on within-family comparisons, more abundant genes (present at least in 5 out of 8 within-family comparisons at either temperature, unless otherwise specified) with significant functions that were expressed in the skin of most or all families at one or both temperatures are discussed in this study, considering their expression trends/levels (see Supplementary File S6). It should be noted that one family (F265 at 10°C and F292 at 20°C) differed between temperature conditions. However, as each comparison was conducted within the same family (adult *vs*. chalimus stages), and the primary aim was to identify genes consistently expressed across families within each temperature, the impact of this substitution on the overall findings is likely minimal. Nonetheless, the potential influence of family-specific responses cannot be fully excluded and should be considered in the interpretation of shared gene expression patterns.

The salmon louse is known to dampen various immune-related responses in susceptible salmonids such as Atlantic salmon (Fast et al., 2006; Wagner et al., 2008; Umasuthan et al., 2020; Øvergård et al., 2023; Øvergård et al., 2025). In this study, immune responses were substantially suppressed/modulated in adult- *versus* chalimus-infested families. For example, the altered transcription of certain suppressor of cytokine signaling (SOCS) proteins (up: *socs1*; down: *socs2*; up-down paralogs: *socs3*) implied that the salmon louse could manipulate cytokine receptor signaling by recruiting SOCS proteins to circumvent the host’s immune defenses (Salisbury et al., 2024). In addition, the transcription levels of multiple genes belonging to the semaphorin family showed significant changes in the skin of families infested by adult *versus* chalimus stages, most of which showed lowered expressions at both temperatures (e.g., *sema3ab*, *sema3c*, *sema3fb*, *sema4d*, *sema6d*). On the other hand, *sema3f* and *sema7a* tended to have different paralogs with reverse expression patterns. The expression levels of plexins (*plxna2*, *plxnb2* [LOC106584474], *plxdc1* and *plxdc2*) and neuropilins (*nrp1a* and *nrp2*), which are known as transmembrane proteins serving as cell surface receptors for semaphorins, were also reduced at both temperatures. The enrichment of GO terms predominantly consisted of semaphorin family members (chemorepellent activity [GO:0045499] and negative chemotaxis [GO:0050919]) that are involved in stimulation of antigen-presenting cells and/or neuroinflammation (Lee et al., 2019; Rajabinejad et al., 2020; Thomas and Yang, 2023) highlighted the immunosuppression and immunoinflammatory disturbance induced by adult forms. Further immunosuppression/modulation resulting from infestation with adult compared to chalimus stages of *L. salmonis* at the attachment sites was supported by differential expression of several immune-related components including representatives of genes related to Toll/NOD-like receptors (up: *sigirr*, *nlrc3l*; down: *tlr21*, *tlr5*, *nlrp1*, *nlrp12*, *card8*, *nlrc3*, *bcl2*), nuclear factors (down: *nfatc2*, *ikbip*, *nfatc1* [in 3 families]), CD molecules (up: *cd276*, *cd44b*, *cd9*; down: *cd2*, *cd6*, *cd3e*, *cd8a*, *cd28*, *cd40*, *cd44*, *cd59*, *cd63*, *cd81*, *cd82*, *cd93*; up-down paralogs: *cd22*, *cd55*), scavenger receptors (down: *scara3*, *scara5*, *cd163*) and protein kinases/phosphatases (up: tab3, *mapk13*, *dusp4*, *dusp5*, *ptpn2b*; down: *map3k11*, *map3k20*, *map3k3*; up-down paralogs: *map3k5*).

The proteinaceous (i.e., trypsin and cathepsin L) and non-proteinaceous (i.e., prostaglandin E2, PGE2) products secreted by the salmon louse are known to interfere with the host’s immune responses and facilitate the flow of blood to the attachment sites (Johnson et al., 2002; Fast et al., 2004; Fast et al., 2007; Firth et al., 2000; McCarthy et al., 2012). Based on within-family comparisons, three subtypes of prostaglandin E2 (PGE2) receptors (EP1, EP2 and EP4 encoded by the genes *ptger1* [down], *ptger2* [up-down paralogs] and *ptger4* [down]) along with prostaglandin D2 receptor 2 (*ptgdr2*, up) and prostaglandin F2 receptor negative regulator (*ptgfrn*, up) were impacted in families infested with adult compared to chalimus stages of lice. Upregulation of the subtype EP2 of PGE2 receptor has been previously reported in the skin of Atlantic salmon infested with copepodids of lice compared to non-infested fish (Øvergård et al., 2023). The reduced expression of EP receptors in fish infested by adult lice may be a consequence of chronic exposure to secretary products (i.e., PGE2) of the salmon louse, resulting in their desensitization or internalization (Malty et al., 2016). The enhanced expression of *pla2g4c*, *ptgis*, *ptgs2b*, *lta4h*, and *ltb4r*, which are involved in lipid mediator synthesis, vasodilation, and/or leukotriene signaling, was observed in nearly all families, indicating a heightened inflammatory response at sites infested by adult lice compared to chalimus stages (Murakami et al., 2003; Ricciotti and FitzGerald, 2011). Dampened expression of *apc*, encoding angiotensin-converting enzyme that promotes vasoconstriction, in all families seemed to be a feedback mechanism to control excessive inflammation. This, however, could allow the parasite to cause chronic infestation by impairing the host’s immune ability. In addition, several other genes correlated with inflammation and inflammasome signaling were also differentially expressed in the skin of most families (up: *gpr84*; down: *nlrp1*, *nlrp12*, *card8*, *nlrc3*, up-down: *nlrp3*, *usp7* and *usp47*). The increased expression of *gpr84* that promotes the release of inflammatory cytokines and reduced expression of *nlrp12*, *card8* and *nlrc3* that negatively regulates inflammation was indicative of excessive inflammatory responses (Zheng et al., 2020; Zhang et al., 2022). On the other hand, a decreased level of *nlrp1*, which is involved in normal release of inflammatory cytokines, accompanied with fluctuated expression of *nlrp3* and its regulators (*usp7* and *usp47*) could lead to a dysregulated status contributing to chronic inflammation or suppressed immune responses (Palazón-Riquelme et al., 2018).

The expression of several mediators associated with acute inflammation were found to be reduced in the skin of fish parasitized with adult compared to chalimus stages of lice (*crp*, *hp*, *a2m*, *ltc4s*). Conversely, *saa5* showed higher expression in the skin of most families at both temperatures. Earlier findings highlighted the upregulation of *saa5* in Atlantic salmon infested by various stages of lice (Sutherland et al., 2014; Braden et al., 2015; Ugelvik and Dalvin, 2022), suggesting that persistent sea lice infestation could result in chronic inflammation, characterized by prolonged expression of pro-inflammatory cytokines such as *saa5*. In addition, higher transcript levels of *il1b* (LOC100136449 and LOC106570815), *il4i1*, *il1r2* and *sigirr* (*il-1r8*) were noticed in most/all families at both temperatures, while *il4/13a* and *il17d* showed reduced transcript expression. Increased mRNA levels of *il1b* and *il8* have been previously shown using qPCR analysis in the skin of both Atlantic salmon and rainbow trout following infestation with juvenile to advanced stages of sea lice including those with no blood-feeding activity (copepodid and/or chalimus) (Dalvin et al., 2020; Ugelvik and Dalvin, 2022). The upregulation of *il1b* (pre-adult-infested *vs*. non-infested) and reduced expression of *il4/13a* (adult *vs*. chalimus) was also reported in the skin of Atlantic salmon in a previous study (Øvergård et al., 2023). This unregulated expression of *il1b* could lead to excessive or prolonged inflammation, as augmented transcriptional levels of *il1r2* (a decoy receptor that antagonizes *il1b*) and *sigirr* (a negative regulator of TLR/IL-1R signalling) were detected in almost all families, possibly to dampen *il-1β*-mediated signaling and protect skin tissue from potential damage (Qin et al., 2005; Schlüter et al., 2018). Interleukin-4 induced 1 (*il4i1*) is an L-amino acid oxidase that produces immunosuppressive metabolites by catabolizing essential amino acids required for T-cell activation/proliferation, and thus, its higher transcript level could be regarded as immunosuppression caused by advanced stages of sea lice (Hirose et al., 2024). The lowered expression of *il4/13a* and *il17d*, which are involved in protection against parasites, was indicative of compromised immunity, tissue healing and barrier function (Sequeida et al., 2020; Huangfu et al., 2023). The expression profile of *il8* was found to be different among families (down in 3 families at 10 and up in 1 family at 20). This could be due to the varied expression level of *il8* in fish infested by adult or chalimus stages, since this gene was shown to be expressed (upregulated) in both fish infested by chalimus and adult stages of lice (compared to un-infested fish) in a previous study (Ugelvik and Dalvin, 2022).

The transcription of several chemokines and their ligands/receptors (up: *ackr4b*, *ccr3*, *cxcr1* and *cxcr2*; down: *ackr3*, *ackr3b*, *ackr4*, *ccr7*, *ccl8*, *ccl13*, *ccl17*, *ccl21*, *ccl25* [only at 20], *ccl25a*, *ccl27a* and *cxcl12a*; up-down paralogs: *ccl4* and *ccl20*) significantly differed between families infested by adult compared to chalimus stages. Similar chemokine-related transcriptomic changes have been previously documented for fish infested by advanced (pre-adult female and adult male) forms of sea lice (Cai et al., 2024). Contrary to inconsistent expression of *il8*, the transcription of *il8* receptors (*cxcr1* and *cxcr2*) that are primary stimulants of neutrophils was enhanced (3-6-fold changes) in most families, potentially contributing to uncontrolled neutrophil responsiveness. Two gene copies related to atypical chemokine receptor 4 represented opposite transcription patterns (up: *ackr4b*; down: *ackr4* [LOC106568919]). Atypical chemokine receptor 4 (*ackr4*), which is expressed primarily in non-immune cells such as keratinocytes, is known to bind and internalize the chemokines *ccl19*, *ccl20*, *ccl21* and *ccl25*, thus regulating the migration and positioning of immune cells (dendritic and T cells) expressing the chemokine receptors *ccr6* (ligand: *ccl20*), *ccr7* (ligands: *ccl19* and *ccl21*) and *ccr9* (ligand: *ccl25*) (Meyrath et al., 2021). Likewise, *ackr3* internalizes and serves as a scavenger receptor for *cxcl11* (receptor: *cxcr3*) and *cxcl12* (receptors: *cxcr4* and *cxcr7*), modulating their availability (Cuesta-Margolles et al., 2024). In addition, the *ccr3* gene encodes a protein primarily found on eosinophils and a subset of Th_2_ lymphocytes, serving as a receptor for chemokines such as eotaxins (*ccl11* and *ccl26*), monocyte chemoattractant protein-3/4 (*ccl7*, *ccl13*) and RANTES (*ccl5*) (Manousou et al., 2010). The dysregulated/contrasting expression of several members of the chemokine family, known for their involvement in directing immune cell chemotaxis, in the skin of fish infested by adult *versus* chalimus stages of lice could favor the recruitment of specific immune cells that aid sea lice persistence. The immunomodulatory effects of salivary products of blood-feeding ticks on chemokine activity of immune cells have been previously reported in terrestrial-associated models *in vivo* and *in vitro* (Vančová et al., 2007; Wang et al., 2007; Oliveira et al., 2008; Langhansova et al., 2015). The association of *ackr4* and *ccr9* in resistance against the ectoparasite *Ichthyophthirius multifiliis* has been also suggested in *Colossoma macropomum* using a genome-wide association study (Lira et al., 2023). These results highlight the importance of identifying louse-derived secretary products that influence the chemokine signalling system in Atlantic salmon to develop possible parasite-protective vaccines.

Some members of the tumor necrosis factor (TNF) cytokine family represented modulated expressions in almost all families (up: *tnfrsf6b* and *tnfrsf9* [LOC106572390]; down: *cd40*, *fas*, *tnfsf11* [LOC106582173], *tnfsf12*, *tnfrsf14* and *traf3ip3*). Paralogs with opposite transcription patterns were identified in all families for *litaf*, a DNA-binding protein that contributes to the progression of inflammation by altering cytokine levels and plays a role in the p53-induced apoptotic pathway (Zou et al., 2015). The transcription of several DEGs associated with apoptosis (up: *aifm1*, *bax*, *casp3b*, *casp3*, *perp*, *malt1*, *tp53*, *api5*, *tigara*; down: *bcl2*, *bcl11ba*, *bcl6b*, *prf1*, *gzma*, *phlda1*, *camk4*), coupled with enrichment of the GO term “p53 signaling pathway”, indicated the induced/dysregulated apoptotic response in damaged/infested cells in families infested (adult *vs*. chalimus) (Wang H. et al., 2023). The transcript levels of genes associated with interferons showed varied expressions in adult- *vs*. chalimus-infested families (up: *ifnar1a*, *ifngr1a*, *ifngr1b*, *irf6*, *irf7*, *irf8*, *ifi44*, *ifit1*, *ifrd2*, *sting1*; down: *ifi30*; up-down: *ifit5*). Genes related to DEAD-box helicases (up: *ddx3x*, *ddx5*, *ddx19a*, *ddx55*, *ddx56*), which act as sensors for dsRNA, cytosolic DNA and viral RNAs (Taschuk and Cherry, 2020), exhibited higher expression in families infested by adult forms. In addition, some TRIM proteins involved in host antiviral defense and/or regulation of interferon signaling (up: *ftr82*, *ftr83*, *trim14*, *trim39*, *isg15*, *uba7*; down: *trim9*, *trim21*, *trim25*, *trim35*) showed altered expressions (van Gent et al., 2018; Wang and Ning, 2021). Modulation of viral-responsive genes has been previously reported in fish infested by various developmental stages of lice (Umasuthan et al., 2020; Cai et al., 2022; Caballero-Solares et al., 2022; Øvergård et al., 2023). Motile-stage sea lice are known to carry divergent RNA viruses, which might account for modulation of interferon-related markers in the skin of Atlantic salmon (Økland et al., 2014; Chang et al., 2023). In addition, two paralogs related to cyclic GMP-AMP synthase gene (*cgas* and *cgasa*) showed higher expression in almost all families. *cgas* is a cytosolic DNA sensor that detects aberrant/foreign DNA from invading pathogens, resulting in type-I interferon production via the cGAS-STING pathway (Ou et al., 2021).

Two paralogs (LOC100136453 and LOC100136439) of the cathelicidin antimicrobial peptide (*camp* or *ll37*) gene were identified to have higher expression in the skin of fish infested with adult compared to chalimus stages, one of which was shared among all families at both temperatures (LOC100136453). In contrast, defensin, beta-like 1 (*defb1*) gene showed lower expression in most families. Similar changes in expression have been also observed in Atlantic salmon following sea lice infestation (Krasnov et al., 2015). Increased expression of *camp* (*as*CATH2) in lice-infested fish was suggested to be a host signal that facilitates the recognition of host by the parasite (Núñez-Acuña et al., 2018). The association of elevated level of *camp* with modulated expression of B-cell lymphoma 3 (*bcl3*), nuclear factor kappa B (NF-κB) and vitamin D receptor (VDR) has been already demonstrated in human keratinocytes (Büchau et al., 2009; Park et al., 2011). A variety of secreted gel-forming and transmembrane mucins (up: *muc17*; down: *muc19*; up-down: *muc2*, *muc3a* and *muc5ac*) were differentially expressed in most/all families. In addition, inconsistent transcript levels of *fcgbp* encoding a mucin-like protein that interacts with *muc2* to preserve the integrity of the mucosal layer were detected among families (Liu et al., 2022). The modulation of *muc2*, *muc4* and *muc5* has been previously reported in lice-infested Atlantic salmon (Caballero-Solares et al., 2022; Gao et al., 2024; Salisbury et al., 2024). The mucus layer contains protective and signal-delivering mucin glycoproteins that are typically secreted upon exposure to various stressors and pathogens (Kufe, 2009). The glycan-rich molecules present on the surface of lice or in their secretions may interact with host mucins by modifying and/or mimicking their structures to persist on the host. Representatives of lectin-associated genes differentially expressed in the skin of families infested with adult *versus* chalimus stages of lice included: *asal* (up); *mrc2*, *pla2r1*, *colec12*, *clec10a* [*mgl*], *clec3ba*, *clec4e* [*mincle*], *clec4m* [*cd209l*], *cd209a*, *cd209c*, *cd209e*, *zg16*, *lgals4*, *lgalsl* and *lgalsla* (down); *cd22*, *mrc1* and *siglec1* (up-down). Despite enhanced expression of the *asal* gene encoding a poorly-characterized mannose-specific lectin, the majority of C-type (CLECs), S-type (galectins) and I-type (siglecs) lectins that are involved in recognition/presentation of pathogen-specific biomolecules such as carbohydrate and chitin represented reduced expression in most families, suggesting persistent immune evasion by the parasite during infestation (Varki and Angata, 2006; Cote et al., 2017; Sousa et al., 2024). In lice-resistant salmonid species (i.e., Coho salmon), multiple classes of C-type lectin receptors (*clec6a*, *clec4e*, and *cd209*) are strongly overexpressed in early stages of infestation, which facilitates the rejection of the attached parasite (Braden et al., 2023). The expression of *nccrp1*, serving as a receptor-like protein expressed in non-specific cytotoxic cells (NCCs), was enhanced in almost all families. *nccrp1* is believed to play a role in the recognition of cells infected by pathogens and activation of cytotoxic responses by activating intracellular signaling pathways, notably the JAK-STAT pathway (Sukeda et al., 2023). This gene has been reported to bind to the receptors of some protozoan parasites and bacterial pathogens (Sukeda et al., 2023; Teng et al., 2023). Several genes related to the complement system were modulated in adult-compared to chalimus-infested families (up: *c1qbp*, *cfh* and *c5ar1*; down: *c1r*, *cd93*, *c4*, *c4b* and *cfd*; up-down: *c3* and *cd55*). *c1qbp* encodes a multifunctional protein involved in mitochondrial fitness, inflammation, apoptosis regulation and ribosome biogenesis (Wang et al., 2022). The elevated expression of *c1qbp* may represent a regulatory mechanism for cGAS activation, as its higher expression was observed in all families (Song et al., 2021). Complement factor H (*cfh*) is an essential inhibitory regulator of the alternative pathway activation, whereas *c5ar1* stimulates chemotaxis in response to C5a (Coulthard et al., 2018; Saxena et al., 2024). All complement-relevant genes with lowered expression were found to be involved in activation of the complement system and innate immune responses. Taken together, these changes implicated the possible suppression/dysfunction of components correlated with complement pathways.

Genes encoding major histocompatibility (MHC) antigens/molecules (down: *bl3-7*, *h2-q10*, *mr1*, *rt1-b*; up-down: *h2aa*) showed differential expression in adult- *versus* chalimus-infested families. MHC class I and II proteins play a pivotal role in the cell-mediated adaptive immune responses, and their reduced/modulated expression suggest diminished immune function (Wieczorek et al., 2017). The expression of immunoglobulin-associated genes was modulated in families infested with adult *versus* chalimus stages (up: *vsig10l* and *vsig8b*; down: *kbas*, *mopc-321*, *lrig1* and *ighd*; up-down: *pigr*). Many of the members of the V-Set and immunoglobulin (Ig) domain-containing (VSIG) protein superfamily represent immunosuppressive properties. For example, both *vsig8b* and *vsig10* can suppress T-cell proliferation and cytokine production (Zhou et al., 2022). Depressed transcript levels of *lrig1* and *ighd* may induce chronic inflammation and reduce antigen binding activity, respectively (Nakamura et al., 2013; Qiu et al., 2021). The gene *pigr* encodes a receptor protein that transports polymeric immunoglobulins (IgM) to the mucosal surface of epithelial cells, and its regulation is controlled by interferons and cytokines (Kong et al., 2018). Some genes belonging to the butyrophilin family showed lower expression in most/all families (*btn1a1*, *btn2a2*, *btn3a1*, *btn3a2*, *btnl2*). Butyrophilins belong to the immunoglobulin superfamily and are associated with the MHC complex, primarily serving as co-signaling molecules involved in T lymphocyte regulation (Malinowska et al., 2017). Most aforementioned butyrophilins (except *btn3a1*) represent inhibitory functions on T-cell proliferation and/or cytokine/interferon production, and their reduced expression may impair tissue repair and contribute to chronic inflammation (Lebrero-Fernández et al., 2016). The expression of T-cell (up: *vtcn1*, *mal* and *pbk*; down: *gzma*, *cd2*, *cd6*, *cd28*, *nfatc2*, *rftn1*, *rftn2*, *trac*, *trbc2*, *trb* and *cabin1*) and B-cell (up: *bcl3* and *bcl10*; down: *bcl6b*, *pbx1* and *pbxip1*; up-down: *bcl11b* and *cd22*) relevant transcripts was modulated in the skin of salmon infested by adult *versus* chalimus. As an immune checkpoint molecule, *vtcn1* negatively regulates the immune reactions by inhibiting T-cell activation/proliferation and cytokine production (Vaishnav et al., 2022). Reduced expression of receptors/antigens essential for T-cell activation, proliferation and/or differentiation (*gzma, cd2, cd6*, *cd28*, *trac*, *trbc2*, *trb*) and *nfatc2* that is a transcription factor implicated in induced expression of cytokine genes in T-cells indicated the repressed adaptive immune responses (Peng et al., 2001; Antonacci et al., 2020; Tian et al., 2022). *bcl10* is a central component of the *card11*-*bcl10*-*malt1* (CBM) signaling complex, which links antigen receptor signaling to NF-κB activation. Likewise, *bcl3* is a context-dependent regulator of T cell responses that controls NF-κB activity (Herrington and Nibbs, 2016). *bcl3* was reported to alter cytokine secretion profile by dendritic cells in response to carbohydrate-expressing pathogens like *Schistosoma mansoni* (through interaction of the parasite sugar ligands with *cd209* [*dc-sign*]), leading to inhibited expression of pro-inflammatory cytokines but enhanced expression of *il10* and chemokines capable of attracting Th_2_ cells (Gringhuis et al., 2014). Similar anti-parasitic responses were also reported in Coho salmon, where Th_1_-type response was associated with lice rejection (Braden et al., 2023). The lack of such responses in Atlantic salmon makes this species prone to infestation with sea lice (Sveen et al., 2025).

Representatives of markers associated with other immune cells (neutrophils, monocytes/macrophages, mast cells and dendritic cells) that were differentially expressed in families infested by different developmental stages of lice included: *lect2*, *ncf2*, *arg1*, *arg2* and *lifr* (up); *lsp1a*, *aif1*, *kit*, *kita*, *csf1a* and *mrc2* (down); *serpinb1*, *mrc1* and *mst1r* (up-down). Of the two *lect2* genes existing in teleost fish, *lect2-b* is highly expressed in mucosal organs, such as the skin and intestine (Hu et al., 2022). Enhanced expression of *lect2*, a multifunctional protein that acts as a chemotactic factor (Lao et al., 2024), was also reported in Atlantic salmon infested with early to advanced stages of sea lice (Skugor et al., 2008; Gallardi et al., 2019; Øvergård et al., 2023). Co-stimulation of *arg1* and *arg2* may contribute to T-cell hyporesponsiveness as a result of depletion of L-arginine required for T-cell proliferation and function (Martí i Líndez and Reith, 2021). *mst1r* (*ron*), mainly expressed in the tissue-resident macrophages, inhibits inflammation and promotes wound healing (Dai et al., 2016). The reduced expression of *mst1r* (loss of *ron* signaling) in most families could result in prolonged inflammation, impaired tissue repair and increased susceptibility to infestation (Hunt et al., 2023). Unlike *mcr1* which is more specialized for pathogen recognition, *mrc2* is involved in collagen turnover process (Melander et al., 2015). The reduced expression of *mrc2* concurrent with modulation of its receptor, i.e., *plaur* (up-down at 10°C and up at 20°C in all families) suggested the potential changes in extracellular matrix (ECM) remodeling and cellular migration (Zhao et al., 2022). *serpinb1* is a protease inhibitor that regulates neutrophil serine proteases, protecting tissues from excessive damage during inflammation (Choi et al., 2019). The transcript levels of several genes corresponding to melanogenesis, melanocyte regulation, melanocyte survival, melanosome transportation and/or skin pigmentation (e.g., *mc5r*, *mlph*, *mreg*, *mitf*, *sox4a*, *sox10*, *pax3*, *pax7*, *pax7a*, *adamts20*, *rab27b*, *rab38*, *kitlg*, *kitlga*, *kit* and *kita*) were found to be reduced in adult-compared to chalimus-infested fish (Kawakami and Fisher, 2017; Xu et al., 2020; Myung et al., 2021; Sevilla and Grichnik, 2024). The *kit* receptor tyrosine kinase and *kit* ligand have been duplicated in teleost, where *kita* and *kitlga* are the paralogs associated with melanogenesis (Braasch et al., 2007; Hultman et al., 2007). Melanin-based pigmentation was suggested to be correlated with immunocompetence and resistance to ectoparasites in Atlantic salmon (Kittilsen et al., 2012). *kit*/*kita* receptors are mainly expressed on melanocytes and mast cells, and their decreased expression might be associated with enhanced levels of *il4* and adrenocorticotropic hormone (ACTH) that suppress melanin production (Pillaiyar et al., 2017).

The wound healing process in fish involves four overlapping phases: re-epithelialization, inflammation, granulation (repair) tissue formation and tissue remodelling (Sveen et al., 2020). Prolonged parasitic infestations can lead to a sustained inflammatory response, which hinders the process of wound healing and promotes the development of chronic wounds (Skugor et al., 2008; Gumede et al., 2024). Chronic wounds are characterized by persistent inflammation (elevated levels of neutrophils and pro-inflammatory macrophages), a reduced proliferative phase and increased proteolytic activity (Wilkinson and Hardman, 2020). The transcription level of a large number of genes involved in ECM architecture and wound healing process/regulation was modulated in adult-compared to chalimus-infested families (up: *tmprss13b*, *anxa1*, *panx1*, *cd44b*, *plat*, *ctnnb1*, *errfi1*, *pim1*, *met*, *myl6* and *tpm3*; down: *eln*, *ogn*, *krt18a*, *krt20*, *krt8.2.l*, *hpse2*, *prss16*, *cd44*, *ctsw*, *ctsl*, *serpine1*, *dact1*, *raf1*, *adra2b*, *ret*, *mras*, *mdkb*, *ccdc80l2*, *ppara* and *pparb2b*, *myh10*; up-down: *col1a1*, *col6a2*, *krt13*, *krt18*, *krt8*, *mylk* and *myo1c*). The majority of structural and non-structural components of ECM revealed reduced expression. For instance, there were 13 DEGs belonging to the collagen family (*col1a1a*, *col1a1b*, *col1a2*, *col2a1*, *col2a1b*, *col5a1*, *col6a3*, *col7a1*, *col10a1*, *col10a1a*, *col11a1*, *col16a1* and *col27a1b*) that consistently showed reduced expression in all families at both temperatures. In addition, matricellular proteins of different families (CCN, SPARC, ENPP, fasciclin, fibulin, olfactomedin, R-spondin, tenascin, thrombospondin) mainly had decreased transcription levels (down: *ccn1*, *ccn2*, *ccn4*, *thbs2a*, *thbs2*, *tnn*, *tnxb*, *postna*, *postn*, *sparc*, *smoc2*, *sparcl1*, *rspo3*, *spon1*, *spon2*, *fbln7*, *abi3bp*, *enpp2* and *adgrl2a*; up-down: *thbs1* and *smoc1*). Unlike structural ECM proteins, matricellular proteins primarily function as modulators of angiogenesis, inflammation and ECM assembly, with their expression significantly increasing at the site of injury to facilitate the repair process (Cárdenas-León et al., 2022). The reduced expression of both structural and non-structural ECM components in the skin can lead to compromised skin integrity, promoted chronic inflammation and delayed wound healing (Cárdenas-León et al., 2022). Transglutaminases (TGs), also known as protein-glutamine γ-glutamyltransferases, catalyze the formation of amide bonds between proteins to form insoluble crosslinked protein structures resistant to proteases degradation (Martins and Choupina, 2018). The expression of some genes related to the transglutaminase family was modulated in most/all families at both temperatures (up: *tgm2*, *tgm3*, *tgm1l1* and *tgm5l*; up-down: *tgm1*). *tgm1*, *tgm3* and *tgm5* are expressed in epithelial tissue and involved in cell-envelope formation during keratinocyte differentiation, whereas *tgm2* is produced in different tissues and plays a role in multiple processes such as wound healing and apoptosis. The induced expression of TGs is associated with extremely stressful conditions and mucosal inflammation/epithelial lesions (Lorand and Graham, 2003; Mehta, 2005). The enrichment of the GO term “keratinization” consisted of representatives of TGs and keratins (*krt8.2.l* and *krt8*) revealed the possible protective role of epidermal keratinization against infestation caused by adult lice. In addition, some genes encoding proteins with important roles in skin barrier function (up: *degs2*, *cldn7b*, *aloxe3*; down: *cldn3c*, *cldn4*, *cldn5*, *cldn5a*, *pnpla1* [only at 10°C]) were modulated in most/all families. *degs2* encodes a bifunctional enzyme that produces ceramides, and *aloxe3* encodes the eLOX3 enzyme that is involved in the production of oxylipins (Krieg et al., 2013; Song et al., 2023). The enhanced expression of *degs2* and *aloxe3* could maintain lipid balance and contribute to the permeable barrier function of the skin against infestation with adult stages. The altered expression of claudins that play a crucial role in maintaining the integrity of tight junctions could result in dysregulated epithelial permeability (Angelow et al., 2008).

A group of matrix metalloproteinase enzymes (up: *mmp9* and *mmp13*; down: *mmp15*, *mmp16* and *mmp23bb*; up-down: *mmp14* and *mmp19*) demonstrated significant transcriptional changes in the skin infested by adult *versus* chalimus stages. *mmp9* and *mmp13* respectively degrade type IV and type II collagens as their preferred substrates (Yabluchanskiy et al., 2013; Hu and Ecker, 2021). *mmp9* (gelatinase B) is secreted by a variety of cell types, including neutrophils, macrophages, and skin keratinocytes/fibroblasts (He et al., 2023), and it can increase mRNA levels of pro-inflammatory cytokines and modulates chemokine signaling, resulting in impaired vascular remodelling and delayed resolution of skin inflammation (Yabluchanskiy et al., 2013). *mmp13* (collagenase 3) which is produced by keratinocytes, fibroblasts and macrophages is abundantly expressed in chronic wounds but not in acute wounds and the normal skin epidermis, suggesting its role in delayed wound healing process (Vaalamo et al., 1997; Saarialho-Kere, 1998). The systematic increase of *mmp9* and *mmp13* has been suggested as a hallmark of transit from acute to chronic inflammation following sea lice infestation (Skugor et al., 2008; Tadiso et al., 2011). Wound-mediated induction of proteinases can not only break down several components of the ECM but also modulate the activity of growth factors (Stamenkovic, 2003). In this regard, multiple genes encoding growth factor-related peptides (TGF-β, IGFs or other growth factors) that have important roles in various phases of wound healing exhibited variable expression levels in adult- *versus* chalimus-infested families (e.g., *tgfb1* [in 4 families], *fgfbp1*, *hbegf*, *vegfaa* [up]; *tgfb2*, *tgfb3*, *tgfbr1*, *ltbp4*, *igf1*, *igfbp5*, *pdgfc*, *pdgfa* [down]). In addition, some genes associated with BMPs, SMAD and WFIKKN proteins (e.g., *bmp2*, *bmp4*, *bmp7*, *smad6*, *smad7*, *wfikkn2*) represented lower expression. *bmp2* and *bmp4* promote angiogenesis and tissue remodeling, whereas *smad6* and *smad7* respectively function as inhibitory regulators of BMP and TGF-β signaling pathways to maintain tissue homeostasis (Goto et al., 2007; Dyer et al., 2014). *wfikkn2* encodes a companion protein that regulates the balance and signaling activity of growth factors such as *tgfb1*, *bmp2* and *bmp4* (Szláma et al., 2010; Monestier and Blanquet, 2016). A variety of genes encoding proteins belonging to ADAMs (up: *adam8*, *adam9* and *adam28*; up-down: *adam10*), ADAMTSs (down: *adamts2*, *adamts2a*, *adamts5*, *adamts10*, *adamts17*, *adamts20*), TIMPs (up: *timp2*; down: *timp3*) and TRIM (e.g., *trim9* [down]; *trim16* [up-down]) were differentially expressed in the skin of adult- *versus* chalimus-infested families. The increased expression of *adam9* may impair keratinocytes migration/proliferation and wound re-epithelialization (Chou et al., 2020), delaying wound closure. *adam10* sheds cell surface proteins such as Notch receptors and mediates E-cadherin shedding in fibroblasts and keratinocytes (Maretzky et al., 2005; Sezin et al., 2022). The elevated levels of ADAMs may contribute to delayed healing by excessive tissue breakdown, promoting sustained inflammation and dysregulated signaling (Wang Z. et al., 2023). Reduced levels of the abovementioned ADAMTSs proteins that are involved in collagen maturation (*adamts2*), regulation of ECM structure (*adamts5*) and formation of microfibrils (*adamts10* and *adamts17*) can lead to chronic wounds, ultimately reducing tissue integrity and delaying the healing process (Kelwick et al., 2015; Karoulias et al., 2020). *trim9* regulates cytoskeletal dynamics and cell migration (Menon et al., 2021), which are crucial for the reorganization and closure of wounds. *trim16* has been shown to be an important regulator of keratinocyte differentiation and proinflammatory cytokine secretion (Ren et al., 2020). The misregulation of TRIM proteins can contribute to improper wound healing and chronic wound pathology.

The expression of genes related to signaling pathways involved in wound healing such as TGF-β, Notch, Wnt/β-catenin, ErbB, Ephrin/Eph, PI3K/Akt, HIF-1, JAK/STAT, MAPK and focal adhesion has been reported to be dysregulated in chronic wounds (Shi et al., 2015; Leyane et al., 2021; Basu and Martins-Green, 2022; Bonnici et al., 2023). In this study, representatives of genes directly or indirectly associated with JNK/Wnt (up: *hif1ab*; down: *wnt3*, *wnt5b*, *wnt6*, *wnt7a*, *mapk8* [*jnk1*], *mycb*, up-down: *lbh*), HIF-1 (up: *hif1ab*, *vegfaa*, *tgfb1* and *nos2b* [*inosb*, in 4 families]; down: *usp20*), Ephrin/Eph (up: *ephb4a*, *epha2* and *efna5*; down: *epha3*, *epha4*, *epha7*, *ephb2*, *efnb3*, *efna2* and *efna5b*; up-down: *efnb1*), ErbB (down: *erbb3* [in 4 families]), Notch (down: *dtx1*), JAK/STAT (down: *stat1*, *stat5a*, *stat5b*), MAPK (up-down: *mapkapk2* and *map4k4*) and focal adhesion (up: *lama3*, *lamb3*, *lamc2*, *itga2*, *itgb3*; down: *lama4*, *itga5*, *itga1*, *itgal*, *itgb1* [*cd29*]) signaling pathways were identified in adult-compared to chalimus-infested families. In addition, several members of semaphorins, ephrins, netrins (*ntn1*, *ntn1a*, *ntn2* and *ntn3*) and slit proteins (*slit1*, *slitrk6*, *srgap*), represented mainly by reduced transcript levels, were enriched in GO/KEGG pathways associated with axon guidance. Axon guidance molecules have been shown to regulate cell migration, angiogenesis and tissue remodeling, and their dysregulation contributes to delayed/impaired wound healing processes (Chilton, 2006; Nakanishi et al., 2022). The increased expression of *hif1ab* that is stabilized during hypoxia concurrent with deceased expression of hemoglobin subunits (*hbb*, *hba4*, *hba1*, *hbb1*) that are involved in oxygen transport and binding suggested the possible hypoxic environment in infested sites, which may contribute to wound persistence and delayed healing. Several genes encoding lysyl oxidase (*lox*) and *lox*-like (*loxl*) proteins showed decreased expression in families infested by adult compared to chalimus stages (*loxa*, *lox*, *loxl2a*, *loxl2b*, *loxl3b*, *loxl4*). Lysyl oxidase (*lox*) proteins facilitate the cross-linking of elastin and collagen fibers, and their altered/dysregulated expression is linked to skin pathologies (Szauter et al., 2005; Laczko and Csiszar, 2020).

As indicated by enrichment analyses, GO terms and KEGG pathways obtained based on comparisons under elevated temperature conditions encompassed a more diverse and frequent set of genes. While this study provides important insights, further research is required to determine whether these gene modulations are driven by elevated temperature, increased lice pathogenicity, or both, as this could not be fully addressed within the scope of the present work. Based on RNA-seq analysis, the transcriptomic responses of salmon families with different lice burden to infestation with adult compared to chalimus stages of lice were not highly dependent to temperature (10/20°C), as the majority of the identified genes were significantly expressed at both temperatures. In addition, the expression patterns resulted from comparisons between families infested by adult *versus* chalimus stages of sea lice were mostly identical at both thermal conditions. On the other hand, the expression level of genes identified based on these comparisons showed important differences. For example, among the genes expressed across all families, several (e.g., *gsr*, *cox2*, *ptgis*, *il1b*, *pla2g4c*, *c1qbp*, *mmp9*, *mmp13*, *casp3b*, *perp*, *tigara*, *ftr83*, *camp*, *itga2*, *tubb2a*, *tubd1* and *gdpd3*) exhibited reduced upregulation, while others (e.g., *tgfb3*, *adamts17*, *adamts20*, *camk1a*, *trim9*, *col16a1*, *col2a1b*, *col10a1a*, *fndc1*, *ncana* and *ltbp4*) represented less pronounced downregulation in F361 at both temperatures. *serpinb1* and *hspa8* were among DEGs with paralogs exhibiting opposite expression patterns, which showed reduced up- and downregulation in F361 compared to other families. Moreover, several genes related to tissue repair (e.g., *mrc2*, *lama3*, *lamc2*, *efna5b*, *serpine1*, *wnt5b*, *itga1*, *ret*, *ptk2ba*, *ptk6*, *ptprj*), growth factor signalling (*stat5a*, *stat5b*, *smad6*, *smad7*, *s100a13*), inflammatory response (e.g., *nlrp1*, *bmp7*, *usp7*), melanogenesis (*kita*), stress/DNA damage response (e.g., *mao*, *maoa*, *api5*, *dnaja*, *dnajb6b*, *hsp10*, *trim39*, *scara3*, *mgst3*) and immune system (e.g., *cd209e*, *sema3a*, *sema4d*, *sema6a*, *sema7a*, *plxdc1*, *btn2a2*, *btn3a1*, *nfatc2*, *c1r*, *cd40*, *tnfsf10*, *tnfsf11*, *tnfsf12*, *tnfrsf14*) were not expressed in F361, while they had modulated (up or down) expression in other families. The qPCR analysis also revealed important differences in the expression level and pattern of *col1a1*, *mmp9* and *saa5* at the attachment site (skin) between F361 and other families at both temperatures. These results collectively suggest that genes associated with immune-inflammatory response, tissue repair, apoptosis, cell adhesion and heat shock response are associated with potential differences among salmon families to sea lice infestation.

## 5 Conclusion

This study presents a novel transcriptomic investigation of Atlantic salmon responses to sea lice infestation, utilizing RNA-seq analysis across multiple salmon families to unravel the complex molecular mechanisms underlying host-parasite interactions. By analyzing the global gene expression profiles of salmon families in response to different sea lice developmental stages (adult *vs*. chalimus) under both normal and elevated temperatures, we have unveiled a substantially more intricate molecular response than previously recognized. The complexity of transcriptomic responses to sea lice infestation in Atlantic salmon is further reflected by the contrasting transcriptional patterns observed among multiple gene paralogs, suggesting possible functional diversification among closely related genes. This genomic complexity further complicates the molecular response mechanisms employed by Atlantic salmon when confronting pathogens such as sea lice. Our findings reveal significant dysregulation of multiple genes and biological pathways, most notably those associated with inflammatory responses, immune system functioning, and wound healing processes. These disruptions fundamentally compromise the host’s ability to mount an effective defense, ultimately contributing to the persistence and survival of sea lice on susceptible hosts. The induced transcriptomic alterations suggest a multifaceted strategy adopted by sea lice that undermines the host’s defensive capabilities.

Interestingly, our analysis indicates that the transcriptomic response to sea lice infestation was not highly temperature-dependent, although elevated temperature potentiated metabolic and stress responses and may potentially exacerbate the impact of infestation over prolonged exposure. This study demonstrates that family-based transcriptomic analysis is a powerful tool for identifying specific molecular markers associated with sea lice infestation and host resistance, which could be used for breeding more resilient salmon populations with improved defense against sea lice infestation. Overall, this research has enhanced our understanding of the molecular intricacies underlying host-parasite interactions in Atlantic salmon, providing a foundation for future research and potential practical applications in sea lice control. The RNA-seq data generated in this study can support genomic selection for sea lice resistance in salmon breeding programs through integration with genome-wide association studies (GWAS) and the application of weighted correlation network analysis (WGCNA) to identify gene modules associated with infestation.

## Data Availability

The RNA‐seq data have been deposited in the NCBI Sequence Read Archive (SRA) under BioProject accession number PRJNA1256531 (https://www.ncbi.nlm.nih.gov/bioproject/1256531).
