# Supplementary material for: Sea lice (Lepeophtheirus salmonis) life stage impacts atlantic salmon transcriptomic responses under different thermal profiles
Source: Front Genet. 2025 Jul 29;16:1633603. doi: 10.3389/fgene.2025.1633603 (PMC12339338; doi:10.3389/fgene.2025.1633603)
Supplement: Supplementary file 7 [file Table2.docx]

**Supplementary Table 2**. Summary of enriched leading GO terms and KEGG pathways based on shared DEGs related to pairwise comparisons within specific families (F419, F361, F175, F265, F292) infected by adult versus chalimus stages of lice at 10 °C or 20 °C (T10A vs. T10C | T20A vs. T20C) in the skin.

| GO Description | GO/KEGG ID | Gene ID | Gene description | Fold-change (log_2_FC) | | | | | | | |
| --- | --- | --- | --- | --- | --- | --- | --- | --- | --- | --- | --- |
|  |  |  |  | T10A vs. T10C | | | | T20A vs. T20C | | | |
|  |  |  |  | F419 | F361 | F175 | F265 | F419 | F361 | F175 | F292 |
| Protein-glutamine gamma-glutamyltransferase activity | GO:0003810 | LOC106597223 | protein-glutamine gamma-glutamyltransferase 2-like | 8.85 | 7.48 | 8.12 | 8.53 | 8.47 | 7.83 | 8.19 | 9.69 |
|  |  | *tgm2* | transglutaminase 2, C polypeptide | 4.43 | 2.36 | 5.00 | 3.10 | 3.85 | 2.64 | 2.13 | 2.09 |
|  |  | LOC106584295 | protein-glutamine gamma-glutamyltransferase K | 4.10 | 1.94 | 1.25 | 2.49 | 2.80 | 1.57 | 2.77 | 3.83 |
|  |  | LOC106583691 | protein-glutamine gamma-glutamyltransferase 2 | 2.14 | 1.34 | 1.51 | 1.92 | 2.29 | 2.16 | 2.93 | 2.98 |
| Glutathione metabolism | KEGG:00480 | LOC106611136 | ornithine decarboxylase 1 | 3.79 | 1.48 | 2.23 | 4.00 | 3.52 | 3.26 | 3.11 | 3.67 |
|  |  | LOC106607664 | ribonucleoside-diphosphate reductase subunit M2 | 3.64 | 1.73 | 3.57 | 3.02 | 3.56 | 2.77 | 3.25 | 3.64 |
|  |  | *pgd* | phosphogluconate dehydrogenase | 2.99 | 1.43 | 2.91 | 2.39 | 2.72 | 1.76 | 2.95 | 3.08 |
|  |  | LOC106586335 | isocitrate dehydrogenase [NADP] cytoplasmic | 2.88 | 1.46 | 2.69 | 2.87 | 2.82 | 2.48 | 2.98 | 3.28 |
|  |  | *rir2* | Ribonucleoside-diphosphate reductase subunit M2 | 2.77 | 1.56 | 3.06 | 2.11 | 2.77 | 2.10 | 2.36 | 2.63 |
|  |  | *gsr* | glutathione reductase | 2.50 | 1.77 | 2.71 | 2.26 | 2.72 | 2.01 | 2.70 | 2.92 |
|  |  | *chac1* | ChaC, cation transport regulator homolog 1 (E. coli) | 2.00 | 1.36 | 1.93 | 2.42 | 3.10 | 2.16 | 2.26 | 3.34 |
| Arachidonic acid metabolism | KEGG:00590 | LOC106577847 | cytosolic phospholipase A2 gamma | 5.20 | 3.21 | 5.23 | 5.85 | 5.01 | 4.00 | 5.02 | 6.17 |
|  |  | LOC106577820 | cytosolic phospholipase A2 gamma-like | 5.10 | 1.75 | 3.33 | 4.75 | 3.86 | 2.15 | 4.32 | 2.63 |
|  |  | *ptgs2b* | prostaglandin-endoperoxide synthase 2b | 3.48 | 1.44 | 1.87 | 1.46 | 3.55 | 2.27 | 3.74 | 4.67 |
|  |  | LOC106583179 | prostacyclin synthase-like | 3.39 | 2.35 | 2.01 | 2.61 | 4.35 | 4.60 | 5.11 | 4.98 |
|  |  | *cbr1* | carbonyl reductase 1 | 2.57 | 1.61 | 2.14 | 2.24 | 2.53 | 2.38 | 2.89 | 2.99 |
|  |  | LOC106590529 | lipocalin-like | -3.34 | -2.18 | -2.83 | -2.91 | -1.97 | -2.02 | -2.16 | -3.13 |
|  |  | LOC106590538 | lipocalin | -4.05 | -2.63 | -2.82 | -4.20 | -1.91 | -2.11 | -2.71 | -3.10 |
| Retinol metabolism | KEGG:00830 | LOC106568881 | epidermal retinol dehydrogenase 2-like | 3.28 | 1.83 | 2.00 | 3.10 | 2.54 | 1.92 | 2.33 | 3.09 |
|  |  | *rdh12* | retinol dehydrogenase 12 (all-trans and 9-cis) | 2.73 | 1.40 | 1.92 | 2.48 | 1.99 | 1.57 | 2.04 | 2.53 |
|  |  | LOC106598856 | retinol dehydrogenase 12 | 1.60 | 1.52 | 1.89 | 2.29 | 1.79 | 1.52 | 1.39 | 1.79 |
|  |  | LOC106567841 | UDP-glucuronosyltransferase-like | -3.17 | -1.83 | -2.93 | -3.08 | -2.24 | -1.75 | -3.15 | -3.77 |
| p53 signaling pathway | KEGG:04115 | *casp3b* | caspase 3, apoptosis-related cysteine peptidase b | 5.20 | 3.66 | 4.29 | 4.49 | 5.43 | 3.76 | 4.18 | 5.72 |
|  |  | LOC106607664 | ribonucleoside-diphosphate reductase subunit M2 | 3.64 | 1.73 | 3.57 | 3.02 | 3.56 | 2.77 | 3.25 | 3.64 |
|  |  | LOC100195405 | cyclin E2 | 3.59 | 1.48 | 3.82 | 3.42 | 3.66 | 2.96 | 3.70 | 3.68 |
|  |  | LOC106566708 | G2/mitotic-specific cyclin-B1 | 3.54 | 1.30 | 3.63 | 3.06 | 3.90 | 3.61 | 3.41 | 3.98 |
|  |  | *ccnb1* | cyclin B1 | 3.44 | 1.50 | 3.74 | 2.93 | 3.62 | 3.51 | 3.23 | 3.74 |
|  |  | LOC106607826 | cyclin-dependent kinase 1-like | 2.96 | 1.30 | 2.75 | 2.13 | 3.30 | 2.93 | 2.66 | 3.18 |
|  |  | *rir2* | Ribonucleoside-diphosphate reductase subunit M2 | 2.77 | 1.56 | 3.06 | 2.11 | 2.77 | 2.10 | 2.36 | 2.63 |
|  |  | LOC106586251 | cytochrome c | 2.77 | 2.13 | 1.69 | 2.79 | 2.86 | 1.74 | 2.40 | 3.13 |
|  |  | LOC100196698 | PERP, TP53 apoptosis effector | 2.47 | 1.35 | 1.79 | 1.97 | 2.16 | 1.79 | 2.32 | 2.58 |
|  |  | *cdc2* | Cell division control protein 2 homolog | 2.12 | 1.28 | 2.42 | 1.68 | 2.64 | 2.65 | 1.81 | 2.34 |
|  |  | *casp3* | Caspase-3 | 1.88 | 1.66 | 1.88 | 1.73 | 1.48 | 1.32 | 1.41 | 1.61 |
| One-carbon metabolic process | GO:0006730 | *mthfd2* | methylenetetrahydrofolate dehydrogenase (NADP+ dependent) 2, methenyltetrahydrofolate cyclohydrolase | 2.73 | 1.36 | 2.11 | 3.24 | 3.38 | 3.00 | 2.86 | 3.67 |
|  |  | LOC100194597 | adenosylhomocysteinase | 2.53 | 1.34 | 2.37 | 1.85 | 1.82 | 1.42 | 1.48 | 1.67 |
|  |  | LOC106580346 | S-adenosylmethionine synthase | 2.48 | 1.57 | 2.56 | 2.67 | 3.69 | 3.38 | 3.52 | 4.75 |
| Collagen binding | GO:0005518 | *lum* | lumican | -3.84 | -1.31 | -3.32 | -2.72 | -1.44 | -1.94 | -2.06 | -2.71 |
|  |  | LOC106612255 | SPARC | -4.18 | -1.28 | -3.58 | -3.18 | -1.64 | -1.93 | -2.15 | -3.11 |
|  |  | LOC106604192 | SPARC | -4.40 | -1.48 | -3.80 | -3.21 | -1.80 | -2.38 | -2.46 | -3.08 |
|  |  | LOC106576454 | lumican | -5.07 | -1.46 | -4.53 | -3.99 | -2.24 | -2.52 | -3.22 | -4.18 |
| Intramolecular transferase activity | GO:0016866 | *lss* | lanosterol synthase (2,3-oxidosqualene-lanosterol cyclase) | 4.30 | 2.75 | 4.29 | 3.84 | 3.80 | 3.42 | 3.46 | 3.67 |
|  |  | LOC106564313 | phosphoglycerate mutase 1 | 3.01 | 1.61 | 2.34 | 2.50 | 2.48 | 1.94 | 2.52 | 2.64 |
|  |  | *pgm3* | phosphoglucomutase 3 | 2.21 | 1.40 | 1.88 | 1.72 | 1.83 | 1.31 | 1.52 | 1.64 |
| Desmosome | GO:0030057 | LOC106599711 | desmoglein-2-like | 4.18 | 2.75 | 3.49 | 3.07 | 4.71 | 3.60 | 4.76 | 4.88 |
|  |  | LOC106569169 | cadherin-4-like | 3.58 | 2.15 | 3.03 | 3.17 | 3.56 | 2.61 | 3.23 | 2.90 |
|  |  | LOC106599708 | desmocollin-2 | 2.60 | 2.15 | 1.36 | 1.99 | 3.15 | 2.85 | 3.14 | 3.90 |
| Centromere complex assembly | GO:0034508 | LOC106562296 | centromere protein N-B | 2.75 | 1.63 | 3.07 | 2.06 | 2.86 | 2.62 | 2.47 | 2.92 |
|  |  | *cenpn* | centromere protein N | 2.63 | 1.50 | 2.67 | 1.49 | 2.57 | 2.36 | 1.80 | 2.55 |
|  |  | *cenph* | centromere protein H | 2.57 | 1.36 | 2.16 | 1.47 | 2.39 | 2.22 | 1.63 | 2.15 |
|  |  | LOC106586549 | centromere protein K-like | 2.04 | 1.37 | 1.68 | 1.41 | 2.84 | 1.67 | 2.35 | 3.28 |
| NADP binding | GO:0050661 | LOC106570829 | 3-hydroxy-3-methylglutaryl-coenzyme A reductase | 3.85 | 3.04 | 3.45 | 4.55 | 3.94 | 3.67 | 3.72 | 4.43 |
|  |  | *dhfr* | dihydrofolate reductase | 3.04 | 1.73 | 2.81 | 2.53 | 2.62 | 2.00 | 2.19 | 2.28 |
|  |  | *pgd* | phosphogluconate dehydrogenase | 2.99 | 1.43 | 2.91 | 2.39 | 2.72 | 1.76 | 2.95 | 3.08 |
|  |  | *gmds* | GDP-mannose 4,6-dehydratase | 2.63 | 1.81 | 2.66 | 2.26 | 2.46 | 2.26 | 2.48 | 2.41 |
|  |  | *gsr* | glutathione reductase | 2.50 | 1.77 | 2.71 | 2.26 | 2.72 | 2.01 | 2.70 | 2.92 |
| Cholesterol homeostasis | GO:0055092; GO:0042632 | LOC106588568 | lanosterol 14-alpha demethylase | 3.61 | 2.58 | 3.83 | 3.34 | 3.44 | 3.09 | 3.18 | 3.19 |
|  |  | LOC106570209 | lanosterol 14-alpha demethylase | 3.52 | 2.55 | 3.67 | 3.52 | 4.00 | 2.96 | 3.45 | 3.49 |
|  |  | LOC106590115 | low-density lipoprotein receptor | 1.78 | 1.70 | 1.48 | 1.61 | 2.13 | 1.71 | 2.16 | 2.18 |
| Dicarboxylic acid metabolic process | KEGG:00020; GO:0043648 | *dhfr* | dihydrofolate reductase | 3.04 | 1.73 | 2.81 | 2.53 | 2.62 | 2.00 | 2.19 | 2.28 |
|  |  | LOC106586335 | isocitrate dehydrogenase [NADP] cytoplasmic | 2.88 | 1.46 | 2.69 | 2.87 | 2.82 | 2.48 | 2.98 | 3.28 |
|  |  | LOC106564857 | malate dehydrogenase, cytoplasmic | 2.83 | 1.25 | 2.67 | 2.59 | 2.73 | 2.81 | 2.97 | 3.18 |
|  |  | LOC106562243 | aspartate aminotransferase, mitochondrial | 2.65 | 1.27 | 2.94 | 2.71 | 2.93 | 2.54 | 2.84 | 2.84 |
|  |  | LOC106590260 | phosphoenolpyruvate carboxykinase 2 (mitochondrial) | 2.44 | 1.34 | 2.96 | 2.95 | 2.86 | 2.96 | 2.81 | 3.38 |
|  |  | *aldh4a1* | aldehyde dehydrogenase 4 family, member A1 | 2.40 | 1.41 | 2.55 | 2.41 | 1.65 | 1.99 | 2.10 | 2.24 |
|  |  | *uroc1* | urocanate hydratase 1 | -1.68 | -1.31 | -1.76 | -1.71 | -1.84 | -1.76 | -1.27 | -1.93 |
| Cyclin-dependent protein kinase holoenzyme complex | GO:0016538; GO:0000307 | LOC100195405 | cyclin E2 | 3.59 | 1.48 | 3.82 | 3.42 | 3.66 | 2.96 | 3.70 | 3.68 |
|  |  | LOC106566708 | G2/mitotic-specific cyclin-B1 | 3.54 | 1.30 | 3.63 | 3.06 | 3.90 | 3.61 | 3.41 | 3.98 |
|  |  | *ccnf* | cyclin F | 3.38 | 1.52 | 3.65 | 3.18 | 3.90 | 3.73 | 3.47 | 4.20 |
|  |  | LOC106611545 | cyclin-A2-like | 2.67 | 1.35 | 2.71 | 1.97 | 2.80 | 2.58 | 2.09 | 2.46 |
|  |  | *ccna2* | cyclin A2 | 2.55 | 1.34 | 3.03 | 2.15 | 3.10 | 2.70 | 2.63 | 2.92 |
| Oxidoreductase activity, acting on the CH-OH group of donors, NAD or NADP as acceptor | GO:0016614; GO:0016616 | LOC106609239 | aldo-keto reductase family 1 member B1 | 4.53 | 2.64 | 3.41 | 3.68 | 3.57 | 1.76 | 3.29 | 4.07 |
|  |  | LOC106604248 | sterol-4-alpha-carboxylate 3-dehydrogenase, decarboxylating-like | 4.08 | 3.01 | 3.90 | 4.19 | 3.16 | 2.69 | 2.73 | 2.97 |
|  |  | LOC106570829 | 3-hydroxy-3-methylglutaryl-coenzyme A reductase | 3.85 | 3.04 | 3.45 | 4.55 | 3.94 | 3.67 | 3.72 | 4.43 |
|  |  | LOC106568881 | epidermal retinol dehydrogenase 2-like | 3.28 | 1.83 | 2.00 | 3.10 | 2.54 | 1.92 | 2.33 | 3.09 |
|  |  | LOC106586211 | D-3-phosphoglycerate dehydrogenase | 3.19 | 1.97 | 4.12 | 4.40 | 5.03 | 4.62 | 4.43 | 5.19 |
|  |  | *pgd* | phosphogluconate dehydrogenase | 2.99 | 1.43 | 2.91 | 2.39 | 2.72 | 1.76 | 2.95 | 3.08 |
|  |  | LOC106612199 | sterol-4-alpha-carboxylate 3-dehydrogenase, decarboxylating | 2.89 | 1.59 | 2.85 | 2.70 | 2.07 | 1.53 | 1.70 | 2.07 |
|  |  | LOC106586335 | isocitrate dehydrogenase [NADP] cytoplasmic | 2.88 | 1.46 | 2.69 | 2.87 | 2.82 | 2.48 | 2.98 | 3.28 |
|  |  | LOC106564857 | malate dehydrogenase, cytoplasmic | 2.83 | 1.25 | 2.67 | 2.59 | 2.73 | 2.81 | 2.97 | 3.18 |
|  |  | LOC106586205 | retinol dehydrogenase 7 | 2.64 | 1.90 | 2.27 | 2.11 | 2.24 | 2.01 | 2.29 | 2.58 |
|  |  | *cbr1* | carbonyl reductase 1 | 2.57 | 1.61 | 2.14 | 2.24 | 2.53 | 2.38 | 2.89 | 2.99 |
|  |  | *hsd17b7* | hydroxysteroid (17-beta) dehydrogenase 7 | 2.14 | 1.75 | 2.47 | 2.65 | 1.83 | 2.03 | 1.84 | 1.45 |
| Carboxy-lyase activity | GO:0016830; GO:0016831 | LOC106611136 | ornithine decarboxylase 1 | 3.79 | 1.48 | 2.23 | 4.00 | 3.52 | 3.26 | 3.11 | 3.67 |
|  |  | *gadl1* | glutamate decarboxylase like 1 | 3.70 | 2.88 | 1.79 | 3.69 | 3.72 | 1.74 | 4.70 | 5.42 |
|  |  | *erg19* | Diphosphomevalonate decarboxylase | 3.20 | 2.07 | 3.07 | 2.82 | 3.35 | 2.39 | 2.96 | 2.80 |
|  |  | *mvda* | mevalonate (diphospho) decarboxylase a | 2.81 | 1.54 | 2.66 | 2.59 | 2.14 | 1.71 | 2.27 | 2.00 |
|  |  | LOC106594092 | acidic amino acid decarboxylase GADL1 | 2.66 | 2.54 | 1.71 | 4.37 | 2.61 | 1.83 | 4.94 | 6.34 |
|  |  | *dcam* | S-adenosylmethionine decarboxylase proenzyme | 2.61 | 1.43 | 2.49 | 2.40 | 1.99 | 1.82 | 2.36 | 2.43 |
|  |  | LOC106590260 | phosphoenolpyruvate carboxykinase 2 (mitochondrial) | 2.44 | 1.34 | 2.96 | 2.95 | 2.86 | 2.96 | 2.81 | 3.38 |
| Cholesterol transport | GO:0015918; GO:0030301 | *stard4* | StAR related lipid transfer domain containing 4 | 3.61 | 1.87 | 3.63 | 3.63 | 3.37 | 3.13 | 3.31 | 3.46 |
|  |  | LOC106562705 | stAR-related lipid transfer protein 5 | 2.17 | 1.83 | 2.31 | 1.83 | 2.88 | 2.55 | 3.42 | 3.20 |
|  |  | LOC106590115 | low-density lipoprotein receptor | 1.78 | 1.70 | 1.48 | 1.61 | 2.13 | 1.71 | 2.16 | 2.18 |
| Steroid dehydrogenase activity, acting on the CH-OH group of donors, NAD or NADP as acceptor | GO:0016229; GO:0033764 | LOC106604248 | sterol-4-alpha-carboxylate 3-dehydrogenase, decarboxylating-like | 4.08 | 3.01 | 3.90 | 4.19 | 3.16 | 2.69 | 2.73 | 2.97 |
|  |  | LOC106612199 | sterol-4-alpha-carboxylate 3-dehydrogenase, decarboxylating | 2.89 | 1.59 | 2.85 | 2.70 | 2.07 | 1.53 | 1.70 | 2.07 |
|  |  | *hsd17b7* | hydroxysteroid (17-beta) dehydrogenase 7 | 2.14 | 1.75 | 2.47 | 2.65 | 1.83 | 2.03 | 1.84 | 1.45 |
| Sphingolipid delta-4 desaturase activity | GO:0016717; GO:0042284; GO:0030148 | LOC106590825 | methylsterol monooxygenase 1 | 3.27 | 2.64 | 3.55 | 3.23 | 3.36 | 2.86 | 3.40 | 3.39 |
|  |  | LOC106571348 | elongation of very long chain fatty acids protein 7-like | 3.06 | 2.25 | 1.75 | 2.94 | 3.88 | 2.36 | 3.97 | 5.72 |
|  |  | LOC106588251 | sphingosine kinase 1-like | 2.87 | 2.32 | 1.68 | 2.53 | 3.48 | 3.70 | 3.59 | 4.52 |
|  |  | LOC106603795 | sphingolipid delta(4)-desaturase/C4-monooxygenase DES2 | 2.78 | 1.40 | 1.68 | 2.37 | 2.49 | 2.69 | 3.19 | 3.39 |
|  |  | *sc5d* | sterol-C5-desaturase | 2.72 | 1.96 | 2.85 | 2.47 | 2.50 | 1.91 | 1.98 | 2.16 |
|  |  | *acer1* | alkaline ceramidase 1 | 2.13 | 1.67 | 2.20 | 2.86 | 2.01 | 2.48 | 1.90 | 2.40 |
| Ensheathment of neurons | GO:0007272; GO:0008366; GO:0042552 | LOC106577171 | myelin and lymphocyte protein-like | 5.27 | 3.96 | 4.33 | 2.93 | 7.18 | 4.64 | 6.98 | 7.21 |
|  |  | *mal* | mal, T-cell differentiation protein | 2.72 | 2.73 | 2.49 | 2.35 | 2.90 | 2.33 | 2.73 | 3.46 |
|  |  | LOC106600490 | myelin protein P0 | -3.55 | -1.56 | -3.37 | -3.74 | -1.28 | -1.73 | -2.91 | -2.18 |
| Fructose and mannose metabolism | KEGG:00051; GO:0051156; GO:0006739 | LOC106589825 | hexokinase-1-like | 3.07 | 1.53 | 1.92 | 2.01 | 2.55 | 1.80 | 2.49 | 2.94 |
|  |  | *pgd* | phosphogluconate dehydrogenase | 2.99 | 1.43 | 2.91 | 2.39 | 2.72 | 1.76 | 2.95 | 3.08 |
|  |  | LOC106586335 | isocitrate dehydrogenase [NADP] cytoplasmic | 2.88 | 1.46 | 2.69 | 2.87 | 2.82 | 2.48 | 2.98 | 3.28 |
|  |  | *tigara* | TP53 induced glycolysis regulatory phosphatase a | 2.69 | 1.82 | 2.86 | 2.38 | 2.92 | 2.57 | 2.90 | 2.90 |
|  |  | *gmds* | GDP-mannose 4,6-dehydratase | 2.63 | 1.81 | 2.66 | 2.26 | 2.46 | 2.26 | 2.48 | 2.41 |
|  |  | *gmppb* | GDP-mannose pyrophosphorylase B | 2.12 | 1.27 | 1.77 | 1.71 | 1.52 | 1.28 | 1.56 | 1.83 |
| Carboxylic acid biosynthetic process | GO:0016053; GO:0046394; GO:0072330; GO:0006636 | LOC106570297 | asparagine synthetase [glutamine-hydrolyzing] | 4.20 | 1.66 | 4.27 | 4.55 | 3.81 | 3.68 | 3.78 | 4.17 |
|  |  | LOC106588568 | lanosterol 14-alpha demethylase | 3.61 | 2.58 | 3.83 | 3.34 | 3.44 | 3.09 | 3.18 | 3.19 |
|  |  | LOC106570209 | lanosterol 14-alpha demethylase | 3.52 | 2.55 | 3.67 | 3.52 | 4.00 | 2.96 | 3.45 | 3.49 |
|  |  | *ptgs2b* | prostaglandin-endoperoxide synthase 2b | 3.48 | 1.44 | 1.87 | 1.46 | 3.55 | 2.27 | 3.74 | 4.67 |
|  |  | LOC106583179 | prostacyclin synthase-like | 3.39 | 2.35 | 2.01 | 2.61 | 4.35 | 4.60 | 5.11 | 4.98 |
|  |  | LOC106586211 | D-3-phosphoglycerate dehydrogenase | 3.19 | 1.97 | 4.12 | 4.40 | 5.03 | 4.62 | 4.43 | 5.19 |
|  |  | LOC106612829 | phosphoserine phosphatase-like | 3.13 | 2.29 | 2.72 | 2.86 | 3.73 | 3.12 | 3.45 | 2.05 |
|  |  | LOC106571348 | elongation of very long chain fatty acids protein 7-like | 3.06 | 2.25 | 1.75 | 2.94 | 3.88 | 2.36 | 3.97 | 5.72 |
|  |  | LOC106585939 | endothelial lipase-like | 2.69 | 1.41 | 1.60 | 1.96 | 2.62 | 2.37 | 2.52 | 3.37 |
|  |  | LOC106562243 | aspartate aminotransferase, mitochondrial | 2.65 | 1.27 | 2.94 | 2.71 | 2.93 | 2.54 | 2.84 | 2.84 |
|  |  | *acot7* | acyl-CoA thioesterase 7 | 2.49 | 1.54 | 2.17 | 2.26 | 2.73 | 2.18 | 1.88 | 2.85 |
|  |  | *psat1* | phosphoserine aminotransferase 1 | 2.31 | 1.54 | 2.46 | 2.75 | 2.62 | 2.59 | 2.15 | 2.87 |
|  |  | LOC106560925 | fatty acid 2-hydroxylase | 2.20 | 1.50 | 1.60 | 1.65 | 1.45 | 1.37 | 2.00 | 2.10 |
|  |  | LOC106573862 | cystathionine gamma-lyase | 2.19 | 1.38 | 2.12 | 1.98 | 1.67 | 1.54 | 2.49 | 2.71 |
| Brassinosteroid metabolic process | GO:0016627; GO:0016628; GO:0016131; GO:0016132 | LOC106587139 | 7-dehydrocholesterol reductase | 4.35 | 3.29 | 4.74 | 3.75 | 4.13 | 3.41 | 3.88 | 3.81 |
|  |  | LOC106603135 | delta(14)-sterol reductase TM7SF2 | 4.34 | 2.25 | 3.88 | 3.65 | 3.98 | 3.13 | 3.78 | 3.88 |
|  |  | *dhcr24* | 24-dehydrocholesterol reductase | 3.89 | 1.77 | 4.23 | 4.16 | 3.51 | 3.29 | 3.89 | 3.76 |
|  |  | LOC106562054 | 7-dehydrocholesterol reductase | 3.76 | 2.88 | 3.90 | 3.43 | 3.23 | 3.37 | 2.99 | 2.98 |
|  |  | LOC106590888 | 3-oxo-5-alpha-steroid 4-dehydrogenase 2-like | -4.83 | -1.56 | -4.25 | -3.31 | -1.83 | -1.99 | -3.14 | -2.37 |
| Glycerophospholipid catabolic process | GO:0046434; GO:0009395; GO:0046503; GO:0046475 | LOC106577847 | cytosolic phospholipase A2 gamma | 5.20 | 3.21 | 5.23 | 5.85 | 5.01 | 4.00 | 5.02 | 6.17 |
|  |  | LOC106577820 | cytosolic phospholipase A2 gamma-like | 5.10 | 1.75 | 3.33 | 4.75 | 3.86 | 2.15 | 4.32 | 2.63 |
|  |  | LOC106600720 | lysophospholipase D GDPD3-like | 3.27 | 1.75 | 3.41 | 3.01 | 2.69 | 2.51 | 3.77 | 2.95 |
|  |  | LOC106589825 | hexokinase-1-like | 3.07 | 1.53 | 1.92 | 2.01 | 2.55 | 1.80 | 2.49 | 2.94 |
|  |  | LOC106564313 | phosphoglycerate mutase 1 | 3.01 | 1.61 | 2.34 | 2.50 | 2.48 | 1.94 | 2.52 | 2.64 |
|  |  | LOC106585939 | endothelial lipase-like | 2.69 | 1.41 | 1.60 | 1.96 | 2.62 | 2.37 | 2.52 | 3.37 |
|  |  | *tigara* | TP53 induced glycolysis regulatory phosphatase a | 2.69 | 1.82 | 2.86 | 2.38 | 2.92 | 2.57 | 2.90 | 2.90 |
|  |  | LOC106562484 | deoxyuridine 5'-triphosphate nucleotidohydrolase, mitochondrial | 2.58 | 1.43 | 2.62 | 2.26 | 2.77 | 2.53 | 2.39 | 2.82 |
|  |  | LOC106562547 | deoxyuridine 5'-triphosphate nucleotidohydrolase-like | 2.26 | 1.63 | 2.45 | 1.67 | 2.76 | 2.38 | 2.28 | 2.50 |
|  |  | LOC106597885 | ectonucleotide pyrophosphatase/phosphodiesterase family member 2-like | -3.67 | -1.76 | -2.72 | -3.07 | -1.75 | -1.49 | -1.35 | -3.18 |
| Complex of collagen trimers | KEGG:04512; GO:0098644; GO:0030199; GO:0098643; GO:0005583 | LOC106568399 | integrin alpha-2-like | 4.34 | 2.59 | 4.06 | 3.32 | 3.59 | 2.57 | 3.97 | 4.45 |
|  |  | LOC106569953 | collagen alpha-1(XI) chain-like | -2.49 | -1.87 | -2.88 | -3.42 | -3.53 | -1.45 | -2.92 | -3.72 |
|  |  | LOC106583712 | collagen alpha-1(II) chain | -2.66 | -1.67 | -2.66 | -1.60 | -1.45 | -1.54 | -1.29 | -2.20 |
|  |  | LOC106586061 | collagen alpha-3(VI) chain | -3.77 | -1.44 | -3.16 | -2.98 | -1.91 | -2.05 | -2.22 | -3.01 |
|  |  | LOC106588939 | collagen alpha-2(I) chain | -4.51 | -1.71 | -3.81 | -3.05 | -1.60 | -2.12 | -2.23 | -3.39 |
|  |  | LOC106600852 | collagen alpha-1(I) chain | -4.73 | -1.66 | -4.01 | -3.19 | -1.68 | -2.29 | -2.40 | -3.51 |
|  |  | LOC106610502 | collagen alpha-1(I) chain | -4.77 | -1.84 | -4.07 | -3.41 | -1.96 | -2.59 | -2.44 | -3.90 |
|  |  | *col2a1b* | collagen, type II, alpha 1b | -4.81 | -1.67 | -5.16 | -4.20 | -1.96 | -1.91 | -3.22 | -3.43 |
|  |  | *col1a1b* | collagen, type I, alpha 1b | -5.03 | -1.76 | -4.14 | -3.35 | -2.26 | -2.78 | -3.16 | -4.77 |
|  |  | *col1a1a* | collagen, type I, alpha 1a | -5.09 | -1.83 | -4.19 | -3.36 | -1.87 | -2.31 | -2.54 | -3.83 |
|  |  | LOC106570460 | collagen alpha-2(I) chain | -5.10 | -1.75 | -4.13 | -3.41 | -1.90 | -2.26 | -2.45 | -3.83 |
|  |  | LOC106589269 | chondroadherin-like | -5.22 | -1.44 | -3.27 | -4.19 | -2.87 | -2.15 | -4.20 | -5.22 |
| Amino sugar and nucleotide sugar metabolism | KEGG:00051; KEGG:00520; GO:0051156; GO:0009225; GO:0009226 | LOC106589825 | hexokinase-1-like | 3.07 | 1.53 | 1.92 | 2.01 | 2.55 | 1.80 | 2.49 | 2.94 |
|  |  | *pgd* | phosphogluconate dehydrogenase | 2.99 | 1.43 | 2.91 | 2.39 | 2.72 | 1.76 | 2.95 | 3.08 |
|  |  | *tigara* | TP53 induced glycolysis regulatory phosphatase a | 2.69 | 1.82 | 2.86 | 2.38 | 2.92 | 2.57 | 2.90 | 2.90 |
|  |  | *gmds* | GDP-mannose 4,6-dehydratase | 2.63 | 1.81 | 2.66 | 2.26 | 2.46 | 2.26 | 2.48 | 2.41 |
|  |  | *nansa* | N-acetylneuraminic acid synthase a | 2.36 | 1.36 | 1.90 | 1.71 | 1.78 | 1.56 | 1.82 | 2.03 |
|  |  | *pgm3* | phosphoglucomutase 3 | 2.21 | 1.40 | 1.88 | 1.72 | 1.83 | 1.31 | 1.52 | 1.64 |
|  |  | *gmppb* | GDP-mannose pyrophosphorylase B | 2.12 | 1.27 | 1.77 | 1.71 | 1.52 | 1.28 | 1.56 | 1.83 |
|  |  | *gfpt1* | glutamine-fructose-6-phosphate transaminase 1 | 2.09 | 1.55 | 2.07 | 1.88 | 1.66 | 1.38 | 1.83 | 1.88 |
|  |  | LOC106610806 | UDP-glucose 4-epimerase-like | 2.08 | 1.49 | 1.88 | 1.71 | 1.58 | 1.39 | 1.72 | 1.85 |
| Nematocyst | GO:0044218; GO:0141061; GO:0031640; GO:0046930; GO:0051715; GO:0042151; GO:0046931 | LOC106601042 | DELTA-stichotoxin-Hcr4a-like | 3.17 | 2.01 | 2.73 | 2.45 | 3.07 | 1.75 | 2.65 | 3.05 |
|  |  | LOC106601040 | bryoporin | 2.99 | 1.70 | 2.34 | 2.15 | 2.64 | 1.83 | 2.39 | 2.86 |
|  |  | LOC106607309 | bryoporin-like | 2.57 | 1.94 | 2.19 | 1.93 | 2.29 | 1.60 | 2.02 | 2.26 |
|  |  | LOC106607311 | DELTA-stichotoxin-Hcr4a | 2.48 | 1.49 | 2.10 | 1.96 | 2.66 | 1.94 | 2.59 | 2.93 |
|  |  | LOC106577200 | voltage-dependent anion-selective channel protein 2 | 2.12 | 1.33 | 1.96 | 1.81 | 2.23 | 1.68 | 2.01 | 2.45 |
| Spindle midzone | GO:0051302; GO:0051233; GO:1902850; GO:0007052; GO:0005876; GO:0090307 | *aurka* | aurora kinase A | 4.08 | 1.35 | 4.06 | 3.50 | 4.37 | 4.14 | 3.98 | 4.62 |
|  |  | LOC106573105 | aurora kinase B-like | 3.86 | 1.68 | 4.08 | 3.13 | 3.95 | 3.76 | 3.75 | 4.41 |
|  |  | LOC106584748 | protein regulator of cytokinesis 1 | 3.26 | 1.29 | 3.43 | 3.04 | 3.53 | 3.24 | 3.06 | 3.68 |
|  |  | LOC106589940 | kinesin-like protein KIF20B | 3.19 | 1.31 | 3.01 | 2.52 | 2.93 | 3.33 | 2.61 | 3.06 |
|  |  | LOC106565211 | rac GTPase-activating protein 1-like | 3.13 | 1.46 | 3.69 | 2.76 | 3.19 | 3.03 | 2.97 | 3.36 |
|  |  | *cenph* | centromere protein H | 2.57 | 1.36 | 2.16 | 1.47 | 2.39 | 2.22 | 1.63 | 2.15 |
|  |  | LOC106611047 | transforming growth factor beta-3 proprotein | -2.72 | -1.42 | -2.68 | -2.85 | -3.09 | -1.96 | -2.51 | -3.20 |
|  |  | LOC106561064 | protein inscuteable homolog | -2.73 | -1.30 | -2.98 | -2.66 | -1.92 | -1.53 | -1.86 | -2.25 |
| Proteinogenic amino acid metabolic process | KEGG:00250; KEGG:00350; KEGG:00360; GO:0016769; GO:0008483; GO:0170039; GO:0009064 | LOC106570297 | asparagine synthetase [glutamine-hydrolyzing] | 4.20 | 1.66 | 4.27 | 4.55 | 3.81 | 3.68 | 3.78 | 4.17 |
|  |  | LOC106586211 | D-3-phosphoglycerate dehydrogenase | 3.19 | 1.97 | 4.12 | 4.40 | 5.03 | 4.62 | 4.43 | 5.19 |
|  |  | LOC106612829 | phosphoserine phosphatase-like | 3.13 | 2.29 | 2.72 | 2.86 | 3.73 | 3.12 | 3.45 | 2.05 |
|  |  | *il4i1* | interleukin 4 induced 1 | 2.90 | 1.56 | 2.27 | 2.10 | 2.10 | 2.04 | 1.93 | 1.34 |
|  |  | LOC106562243 | aspartate aminotransferase, mitochondrial | 2.65 | 1.27 | 2.94 | 2.71 | 2.93 | 2.54 | 2.84 | 2.84 |
|  |  | LOC106601716 | aldehyde dehydrogenase family 3 member B1-like | 2.54 | 1.86 | 2.82 | 2.96 | 2.62 | 2.62 | 3.04 | 2.91 |
|  |  | LOC106596374 | aldehyde dehydrogenase family 3 member B1-like | 2.48 | 1.68 | 2.43 | 2.94 | 2.76 | 2.51 | 2.79 | 2.84 |
|  |  | *aldh4a1* | aldehyde dehydrogenase 4 family, member A1 | 2.40 | 1.41 | 2.55 | 2.41 | 1.65 | 1.99 | 2.10 | 2.24 |
|  |  | *psat1* | phosphoserine aminotransferase 1 | 2.31 | 1.54 | 2.46 | 2.75 | 2.62 | 2.59 | 2.15 | 2.87 |
|  |  | LOC106573862 | cystathionine gamma-lyase | 2.19 | 1.38 | 2.12 | 1.98 | 1.67 | 1.54 | 2.49 | 2.71 |
|  |  | *gfpt1* | glutamine-fructose-6-phosphate transaminase 1 | 2.09 | 1.55 | 2.07 | 1.88 | 1.66 | 1.38 | 1.83 | 1.88 |
|  |  | *uroc1* | urocanate hydratase 1 | -1.68 | -1.31 | -1.76 | -1.71 | -1.84 | -1.76 | -1.27 | -1.93 |
| Isoprenoid biosynthetic process | GO:0016765; GO:0004659; GO:0006720; GO:0008299; GO:0006721; GO:0016114; GO:0045338; GO:0045337 | *lss* | lanosterol synthase (2,3-oxidosqualene-lanosterol cyclase) | 4.30 | 2.75 | 4.29 | 3.84 | 3.80 | 3.42 | 3.46 | 3.67 |
|  |  | *hmgcs1* | 3-hydroxy-3-methylglutaryl-CoA synthase 1 (soluble) | 4.10 | 3.08 | 4.21 | 3.70 | 3.38 | 3.12 | 3.27 | 3.46 |
|  |  | LOC106570829 | 3-hydroxy-3-methylglutaryl-coenzyme A reductase | 3.85 | 3.04 | 3.45 | 4.55 | 3.94 | 3.67 | 3.72 | 4.43 |
|  |  | LOC106605924 | farnesyl pyrophosphate synthase | 3.31 | 2.23 | 3.03 | 3.20 | 2.93 | 2.17 | 2.55 | 2.71 |
|  |  | *fpps* | Farnesyl pyrophosphate synthetase | 3.29 | 2.15 | 3.05 | 3.35 | 2.65 | 2.29 | 2.65 | 2.78 |
|  |  | *erg19* | Diphosphomevalonate decarboxylase | 3.20 | 2.07 | 3.07 | 2.82 | 3.35 | 2.39 | 2.96 | 2.80 |
|  |  | *fdft1* | farnesyl-diphosphate farnesyltransferase 1 | 2.83 | 1.81 | 3.23 | 2.66 | 2.65 | 2.24 | 2.47 | 2.65 |
|  |  | *mvda* | mevalonate (diphospho) decarboxylase a | 2.81 | 1.54 | 2.66 | 2.59 | 2.14 | 1.71 | 2.27 | 2.00 |
|  |  | *acat2* | acetyl-CoA acetyltransferase 2 | 2.64 | 1.75 | 3.08 | 2.66 | 2.31 | 2.16 | 2.15 | 2.06 |
|  |  | LOC106586205 | retinol dehydrogenase 7 | 2.64 | 1.90 | 2.27 | 2.11 | 2.24 | 2.01 | 2.29 | 2.58 |
|  |  | *idi1* | isopentenyl-diphosphate delta isomerase 1 | 2.58 | 2.28 | 2.32 | 1.95 | 2.26 | 1.55 | 1.56 | 1.79 |
|  |  | LOC106580346 | S-adenosylmethionine synthase | 2.48 | 1.57 | 2.56 | 2.67 | 3.69 | 3.38 | 3.52 | 4.75 |
|  |  | *nansa* | N-acetylneuraminic acid synthase a | 2.36 | 1.36 | 1.90 | 1.71 | 1.78 | 1.56 | 1.82 | 2.03 |
|  |  | LOC106585666 | mevalonate kinase | 2.23 | 1.72 | 2.57 | 1.91 | 1.79 | 1.71 | 1.66 | 1.29 |
| Phenylalanine metabolism | KEGG:00250; KEGG:00340; KEGG:00350; KEGG:00360; KEGG:00410; KEGG:00980; KEGG:00982; GO:0016903; GO:0070279; GO:0016620; GO:0030170 | LOC106570297 | asparagine synthetase [glutamine-hydrolyzing] | 4.20 | 1.66 | 4.27 | 4.55 | 3.81 | 3.68 | 3.78 | 4.17 |
|  |  | *gadl1* | glutamate decarboxylase like 1 | 3.70 | 2.88 | 1.79 | 3.69 | 3.72 | 1.74 | 4.70 | 5.42 |
|  |  | *il4i1* | interleukin 4 induced 1 | 2.90 | 1.56 | 2.27 | 2.10 | 2.10 | 2.04 | 1.93 | 1.34 |
|  |  | LOC106594092 | acidic amino acid decarboxylase GADL1 | 2.66 | 2.54 | 1.71 | 4.37 | 2.61 | 1.83 | 4.94 | 6.34 |
|  |  | LOC106562243 | aspartate aminotransferase, mitochondrial | 2.65 | 1.27 | 2.94 | 2.71 | 2.93 | 2.54 | 2.84 | 2.84 |
|  |  | *cbr1* | carbonyl reductase 1 | 2.57 | 1.61 | 2.14 | 2.24 | 2.53 | 2.38 | 2.89 | 2.99 |
|  |  | LOC106601716 | aldehyde dehydrogenase family 3 member B1-like | 2.54 | 1.86 | 2.82 | 2.96 | 2.62 | 2.62 | 3.04 | 2.91 |
|  |  | LOC106596374 | aldehyde dehydrogenase family 3 member B1-like | 2.48 | 1.68 | 2.43 | 2.94 | 2.76 | 2.51 | 2.79 | 2.84 |
|  |  | *aldh4a1* | aldehyde dehydrogenase 4 family, member A1 | 2.40 | 1.41 | 2.55 | 2.41 | 1.65 | 1.99 | 2.10 | 2.24 |
|  |  | *psat1* | phosphoserine aminotransferase 1 | 2.31 | 1.54 | 2.46 | 2.75 | 2.62 | 2.59 | 2.15 | 2.87 |
|  |  | LOC106573862 | cystathionine gamma-lyase | 2.19 | 1.38 | 2.12 | 1.98 | 1.67 | 1.54 | 2.49 | 2.71 |
|  |  | *gfpt1* | glutamine-fructose-6-phosphate transaminase 1 | 2.09 | 1.55 | 2.07 | 1.88 | 1.66 | 1.38 | 1.83 | 1.88 |
|  |  | *uroc1* | urocanate hydratase 1 | -1.68 | -1.31 | -1.76 | -1.71 | -1.84 | -1.76 | -1.27 | -1.93 |
|  |  | LOC106567841 | UDP-glucuronosyltransferase-like | -3.17 | -1.83 | -2.93 | -3.08 | -2.24 | -1.75 | -3.15 | -3.77 |
| Skin development | GO:0008544; GO:0043588; GO:0030855; GO:0045104; GO:0045109; GO:0009913; GO:0031424; GO:0030216; GO:0045111; GO:0005882; GO:0045095 | LOC106600778 | cell wall protein IFF6 | 5.52 | 5.40 | 6.40 | 6.56 | 4.30 | 7.85 | 5.74 | 7.15 |
|  |  | LOC106565226 | keratin, type II cytoskeletal 8 | 5.26 | 3.02 | 4.60 | 4.27 | 5.84 | 5.23 | 5.96 | 6.83 |
|  |  | *casp3b* | caspase 3, apoptosis-related cysteine peptidase b | 5.20 | 3.66 | 4.29 | 4.49 | 5.43 | 3.76 | 4.18 | 5.72 |
|  |  | LOC106600814 | keratin, type I cytoskeletal 13-like | 4.48 | 5.50 | 5.53 | 5.95 | 5.14 | 7.82 | 7.43 | 8.67 |
|  |  | LOC106584295 | protein-glutamine gamma-glutamyltransferase K | 4.10 | 1.94 | 1.25 | 2.49 | 2.80 | 1.57 | 2.77 | 3.83 |
|  |  | LOC106572756 | keratin, type I cytoskeletal 18 | 3.86 | 2.05 | 2.97 | 1.99 | 4.59 | 2.75 | 3.70 | 3.66 |
|  |  | LOC106564958 | keratin, type I cytoskeletal 13 | 3.30 | 2.70 | 3.97 | 2.89 | 4.47 | 3.14 | 3.88 | 4.18 |
|  |  | LOC106600816 | keratin, type I cytoskeletal 13 | 2.99 | 2.79 | 4.07 | 3.17 | 4.72 | 4.03 | 4.45 | 5.17 |
|  |  | *eppk1* | epiplakin 1 | 2.95 | 1.82 | 2.22 | 1.44 | 3.75 | 2.32 | 3.01 | 3.44 |
|  |  | LOC106590260 | phosphoenolpyruvate carboxykinase 2 (mitochondrial) | 2.44 | 1.34 | 2.96 | 2.95 | 2.86 | 2.96 | 2.81 | 3.38 |
|  |  | *casp3* | Caspase-3 | 1.88 | 1.66 | 1.88 | 1.73 | 1.48 | 1.32 | 1.41 | 1.61 |
|  |  | *scel* | sciellin | 1.85 | 1.46 | 1.37 | 1.67 | 1.47 | 1.29 | 1.60 | 2.03 |
|  |  | LOC106583605 | keratin, type II cytoskeletal 8 | -3.37 | -1.39 | -3.06 | -2.77 | -1.76 | -1.98 | -2.15 | -2.88 |
|  |  | *col1a1a* | collagen, type I, alpha 1a | -5.09 | -1.83 | -4.19 | -3.36 | -1.87 | -2.31 | -2.54 | -3.83 |
| Extracellular matrix structural constituent conferring tensile strength | KEGG:04512; GO:0030020; GO:0043062; GO:0045229; GO:0098644; GO:0030198; GO:0098651; GO:0005604; GO:0098643; GO:0098645; GO:0098642; GO:0005583; GO:0005587 | LOC106568399 | integrin alpha-2-like | 4.34 | 2.59 | 4.06 | 3.32 | 3.59 | 2.57 | 3.97 | 4.45 |
|  |  | *mmp9* | matrix metalloproteinase 9 | 4.02 | 2.40 | 3.93 | 2.09 | 3.86 | 1.28 | 3.74 | 4.97 |
|  |  | LOC106579812 | collagen alpha-1(XXVII) chain B-like | -1.43 | -1.36 | -2.04 | -1.70 | -1.59 | -1.40 | -1.73 | -2.02 |
|  |  | LOC106583145 | collagen alpha-1(VII) chain | -2.08 | -1.27 | -1.78 | -2.18 | -2.95 | -1.72 | -2.45 | -3.87 |
|  |  | LOC106567192 | matrix metalloproteinase-19-like | -2.47 | -1.56 | -3.30 | -2.36 | -1.51 | -2.15 | -2.26 | -2.42 |
|  |  | LOC106569953 | collagen alpha-1(XI) chain-like | -2.49 | -1.87 | -2.88 | -3.42 | -3.53 | -1.45 | -2.92 | -3.72 |
|  |  | LOC106583712 | collagen alpha-1(II) chain | -2.66 | -1.67 | -2.66 | -1.60 | -1.45 | -1.54 | -1.29 | -2.20 |
|  |  | *adamts2a* | ADAM metallopeptidase with thrombospondin type 1 motif, 2a | -2.77 | -1.29 | -2.34 | -2.65 | -1.87 | -2.04 | -2.13 | -3.45 |
|  |  | LOC106562962 | collagen alpha-1(V) chain | -3.14 | -1.31 | -3.30 | -2.59 | -1.60 | -1.83 | -1.88 | -2.44 |
|  |  | LOC106562591 | A disintegrin and metalloproteinase with thrombospondin motifs 17-like | -3.77 | -1.80 | -3.40 | -2.88 | -3.09 | -1.92 | -2.10 | -4.34 |
|  |  | LOC106586061 | collagen alpha-3(VI) chain | -3.77 | -1.44 | -3.16 | -2.98 | -1.91 | -2.05 | -2.22 | -3.01 |
|  |  | LOC106563762 | A disintegrin and metalloproteinase with thrombospondin motifs 2-like | -3.82 | -1.51 | -3.84 | -3.29 | -1.96 | -1.77 | -2.49 | -3.06 |
|  |  | LOC106586705 | target of Nesh-SH3 | -4.05 | -1.45 | -3.45 | -3.71 | -2.19 | -2.02 | -2.86 | -3.63 |
|  |  | LOC106592099 | collagen alpha-1(XI) chain-like | -4.15 | -1.36 | -3.79 | -3.55 | -5.11 | -3.04 | -3.91 | -5.34 |
|  |  | *ccdc80l2* | coiled-coil domain containing 80 like 2 | -4.18 | -1.37 | -3.50 | -3.22 | -1.92 | -1.66 | -2.76 | -3.54 |
|  |  | LOC106612255 | SPARC | -4.18 | -1.28 | -3.58 | -3.18 | -1.64 | -1.93 | -2.15 | -3.11 |
|  |  | LOC106604192 | SPARC | -4.40 | -1.48 | -3.80 | -3.21 | -1.80 | -2.38 | -2.46 | -3.08 |
|  |  | LOC106588939 | collagen alpha-2(I) chain | -4.51 | -1.71 | -3.81 | -3.05 | -1.60 | -2.12 | -2.23 | -3.39 |
|  |  | LOC106600852 | collagen alpha-1(I) chain | -4.73 | -1.66 | -4.01 | -3.19 | -1.68 | -2.29 | -2.40 | -3.51 |
|  |  | LOC106610502 | collagen alpha-1(I) chain | -4.77 | -1.84 | -4.07 | -3.41 | -1.96 | -2.59 | -2.44 | -3.90 |
|  |  | *col2a1b* | collagen, type II, alpha 1b | -4.81 | -1.67 | -5.16 | -4.20 | -1.96 | -1.91 | -3.22 | -3.43 |
|  |  | *col1a1b* | collagen, type I, alpha 1b | -5.03 | -1.76 | -4.14 | -3.35 | -2.26 | -2.78 | -3.16 | -4.77 |
|  |  | *col1a1a* | collagen, type I, alpha 1a | -5.09 | -1.83 | -4.19 | -3.36 | -1.87 | -2.31 | -2.54 | -3.83 |
|  |  | LOC106570545 | collagen alpha-1(XVI) chain | -5.10 | -1.49 | -4.37 | -3.98 | -2.25 | -1.77 | -2.65 | -3.56 |
|  |  | LOC106570460 | collagen alpha-2(I) chain | -5.10 | -1.75 | -4.13 | -3.41 | -1.90 | -2.26 | -2.45 | -3.83 |
|  |  | LOC106589269 | chondroadherin-like | -5.22 | -1.44 | -3.27 | -4.19 | -2.87 | -2.15 | -4.20 | -5.22 |
| Pyrimidine metabolism | KEGG:00240; KEGG:00983; GO:0072527; GO:0009116; GO:0019205; GO:0072529; GO:0009123; GO:0009262; GO:0009124; GO:0009162; GO:0009263; GO:0009157; GO:0019206; GO:0019136 | *cdd* | Cytidine deaminase | 5.64 | 3.68 | 4.47 | 4.62 | 5.28 | 3.11 | 4.08 | 4.76 |
|  |  | LOC106607664 | ribonucleoside-diphosphate reductase subunit M2 | 3.64 | 1.73 | 3.57 | 3.02 | 3.56 | 2.77 | 3.25 | 3.64 |
|  |  | *upp1* | uridine phosphorylase 1 | 3.50 | 1.82 | 2.69 | 3.14 | 3.10 | 2.57 | 2.85 | 2.90 |
|  |  | LOC106608205 | cytosolic 5'-nucleotidase 1A-like | 3.02 | 2.03 | 1.95 | 2.34 | 2.59 | 2.00 | 2.76 | 2.99 |
|  |  | *rir2* | Ribonucleoside-diphosphate reductase subunit M2 | 2.77 | 1.56 | 3.06 | 2.11 | 2.77 | 2.10 | 2.36 | 2.63 |
|  |  | LOC106562484 | deoxyuridine 5'-triphosphate nucleotidohydrolase, mitochondrial | 2.58 | 1.43 | 2.62 | 2.26 | 2.77 | 2.53 | 2.39 | 2.82 |
|  |  | zgc:110540 | deoxynucleoside kinase | 2.29 | 1.38 | 2.68 | 1.76 | 2.20 | 1.85 | 1.73 | 2.27 |
|  |  | LOC106562547 | deoxyuridine 5'-triphosphate nucleotidohydrolase-like | 2.26 | 1.63 | 2.45 | 1.67 | 2.76 | 2.38 | 2.28 | 2.50 |
|  |  | *dck* | deoxycytidine kinase | 2.03 | 1.59 | 1.88 | 1.68 | 2.34 | 1.58 | 1.64 | 1.93 |
|  |  | *tk1* | thymidine kinase 1, soluble | 1.52 | 1.32 | 1.81 | 1.84 | 2.38 | 1.89 | 1.90 | 2.13 |
|  |  | LOC106567841 | UDP-glucuronosyltransferase-like | -3.17 | -1.83 | -2.93 | -3.08 | -2.24 | -1.75 | -3.15 | -3.77 |
|  |  | *entpd5a* | ectonucleoside triphosphate diphosphohydrolase 5a | -7.30 | -2.78 | -6.08 | -5.86 | -5.97 | -2.54 | -3.93 | -5.32 |
| Tetrahydrofolate biosynthetic process | KEGG:00790; GO:0016814; GO:0042558; GO:0006760; GO:0019751; GO:0042398; GO:0019238; GO:0042559; GO:0009396; GO:0034311; GO:0046173; GO:0046653; GO:0034312; GO:0046654 | *cdd* | Cytidine deaminase | 5.64 | 3.68 | 4.47 | 4.62 | 5.28 | 3.11 | 4.08 | 4.76 |
|  |  | *dhfr* | dihydrofolate reductase | 3.04 | 1.73 | 2.81 | 2.53 | 2.62 | 2.00 | 2.19 | 2.28 |
|  |  | LOC106588251 | sphingosine kinase 1-like | 2.87 | 2.32 | 1.68 | 2.53 | 3.48 | 3.70 | 3.59 | 4.52 |
|  |  | *mthfd2* | methylenetetrahydrofolate dehydrogenase (NADP+ dependent) 2, methenyltetrahydrofolate cyclohydrolase | 2.73 | 1.36 | 2.11 | 3.24 | 3.38 | 3.00 | 2.86 | 3.67 |
|  |  | LOC106602973 | GTP cyclohydrolase 1 | 2.65 | 2.25 | 2.51 | 1.96 | 2.85 | 2.85 | 2.82 | 3.43 |
|  |  | *cbr1* | carbonyl reductase 1 | 2.57 | 1.61 | 2.14 | 2.24 | 2.53 | 2.38 | 2.89 | 2.99 |
|  |  | LOC106590260 | phosphoenolpyruvate carboxykinase 2 (mitochondrial) | 2.44 | 1.34 | 2.96 | 2.95 | 2.86 | 2.96 | 2.81 | 3.38 |
|  |  | *pudp* | pseudouridine 5'-phosphatase | 2.43 | 1.33 | 2.76 | 2.36 | 2.29 | 1.76 | 2.09 | 2.26 |
|  |  | *acer1* | alkaline ceramidase 1 | 2.13 | 1.67 | 2.20 | 2.86 | 2.01 | 2.48 | 1.90 | 2.40 |
|  |  | LOC106605086 | GTP cyclohydrolase 1 | -2.34 | -1.97 | -2.59 | -1.34 | -1.53 | -2.21 | -1.68 | -4.50 |
| Small molecule biosynthetic process | KEGG:00250; KEGG:00260; KEGG:00270; KEGG:00410; GO:0016769; GO:0044283; GO:0070279; GO:0008483; GO:0008652; GO:0016053; GO:0030170; GO:0170039; GO:0046394; GO:0170038; GO:1901607; GO:0009064; GO:0009069; GO:0072330; GO:0170034; GO:0006563; GO:0009070; GO:0006564 | *ebp* | EBP cholestenol delta-isomerase | 5.02 | 3.30 | 4.74 | 4.97 | 4.77 | 3.77 | 4.55 | 4.66 |
|  |  | LOC106587139 | 7-dehydrocholesterol reductase | 4.35 | 3.29 | 4.74 | 3.75 | 4.13 | 3.41 | 3.88 | 3.81 |
|  |  | LOC106603135 | delta(14)-sterol reductase TM7SF2 | 4.34 | 2.25 | 3.88 | 3.65 | 3.98 | 3.13 | 3.78 | 3.88 |
|  |  | *lss* | lanosterol synthase (2,3-oxidosqualene-lanosterol cyclase) | 4.30 | 2.75 | 4.29 | 3.84 | 3.80 | 3.42 | 3.46 | 3.67 |
|  |  | LOC106570297 | asparagine synthetase [glutamine-hydrolyzing] | 4.20 | 1.66 | 4.27 | 4.55 | 3.81 | 3.68 | 3.78 | 4.17 |
|  |  | *hmgcs1* | 3-hydroxy-3-methylglutaryl-CoA synthase 1 (soluble) | 4.10 | 3.08 | 4.21 | 3.70 | 3.38 | 3.12 | 3.27 | 3.46 |
|  |  | LOC106570829 | 3-hydroxy-3-methylglutaryl-coenzyme A reductase | 3.85 | 3.04 | 3.45 | 4.55 | 3.94 | 3.67 | 3.72 | 4.43 |
|  |  | LOC106562054 | 7-dehydrocholesterol reductase | 3.76 | 2.88 | 3.90 | 3.43 | 3.23 | 3.37 | 2.99 | 2.98 |
|  |  | *gadl1* | glutamate decarboxylase like 1 | 3.70 | 2.88 | 1.79 | 3.69 | 3.72 | 1.74 | 4.70 | 5.42 |
|  |  | LOC106588568 | lanosterol 14-alpha demethylase | 3.61 | 2.58 | 3.83 | 3.34 | 3.44 | 3.09 | 3.18 | 3.19 |
|  |  | LOC106605546 | squalene monooxygenase | 3.57 | 3.09 | 3.79 | 3.50 | 3.44 | 3.02 | 3.40 | 3.45 |
|  |  | LOC106570209 | lanosterol 14-alpha demethylase | 3.52 | 2.55 | 3.67 | 3.52 | 4.00 | 2.96 | 3.45 | 3.49 |
|  |  | *ptgs2b* | prostaglandin-endoperoxide synthase 2b | 3.48 | 1.44 | 1.87 | 1.46 | 3.55 | 2.27 | 3.74 | 4.67 |
|  |  | LOC106583179 | prostacyclin synthase-like | 3.39 | 2.35 | 2.01 | 2.61 | 4.35 | 4.60 | 5.11 | 4.98 |
|  |  | LOC106590825 | methylsterol monooxygenase 1 | 3.27 | 2.64 | 3.55 | 3.23 | 3.36 | 2.86 | 3.40 | 3.39 |
|  |  | *erg19* | Diphosphomevalonate decarboxylase | 3.20 | 2.07 | 3.07 | 2.82 | 3.35 | 2.39 | 2.96 | 2.80 |
|  |  | LOC106586211 | D-3-phosphoglycerate dehydrogenase | 3.19 | 1.97 | 4.12 | 4.40 | 5.03 | 4.62 | 4.43 | 5.19 |
|  |  | LOC106579093 | squalene monooxygenase | 3.17 | 2.43 | 3.62 | 3.34 | 2.89 | 2.97 | 3.38 | 3.07 |
|  |  | LOC106612829 | phosphoserine phosphatase-like | 3.13 | 2.29 | 2.72 | 2.86 | 3.73 | 3.12 | 3.45 | 2.05 |
|  |  | LOC106571348 | elongation of very long chain fatty acids protein 7-like | 3.06 | 2.25 | 1.75 | 2.94 | 3.88 | 2.36 | 3.97 | 5.72 |
|  |  | LOC106564313 | phosphoglycerate mutase 1 | 3.01 | 1.61 | 2.34 | 2.50 | 2.48 | 1.94 | 2.52 | 2.64 |
|  |  | *il4i1* | interleukin 4 induced 1 | 2.90 | 1.56 | 2.27 | 2.10 | 2.10 | 2.04 | 1.93 | 1.34 |
|  |  | LOC106588251 | sphingosine kinase 1-like | 2.87 | 2.32 | 1.68 | 2.53 | 3.48 | 3.70 | 3.59 | 4.52 |
|  |  | LOC106564857 | malate dehydrogenase, cytoplasmic | 2.83 | 1.25 | 2.67 | 2.59 | 2.73 | 2.81 | 2.97 | 3.18 |
|  |  | *fdft1* | farnesyl-diphosphate farnesyltransferase 1 | 2.83 | 1.81 | 3.23 | 2.66 | 2.65 | 2.24 | 2.47 | 2.65 |
|  |  | *mvda* | mevalonate (diphospho) decarboxylase a | 2.81 | 1.54 | 2.66 | 2.59 | 2.14 | 1.71 | 2.27 | 2.00 |
|  |  | *sc5d* | sterol-C5-desaturase | 2.72 | 1.96 | 2.85 | 2.47 | 2.50 | 1.91 | 1.98 | 2.16 |
|  |  | LOC106585939 | endothelial lipase-like | 2.69 | 1.41 | 1.60 | 1.96 | 2.62 | 2.37 | 2.52 | 3.37 |
|  |  | LOC106594092 | acidic amino acid decarboxylase GADL1 | 2.66 | 2.54 | 1.71 | 4.37 | 2.61 | 1.83 | 4.94 | 6.34 |
|  |  | LOC106562243 | aspartate aminotransferase, mitochondrial | 2.65 | 1.27 | 2.94 | 2.71 | 2.93 | 2.54 | 2.84 | 2.84 |
|  |  | LOC106602973 | GTP cyclohydrolase 1 | 2.65 | 2.25 | 2.51 | 1.96 | 2.85 | 2.85 | 2.82 | 3.43 |
|  |  | *acat2* | acetyl-CoA acetyltransferase 2 | 2.64 | 1.75 | 3.08 | 2.66 | 2.31 | 2.16 | 2.15 | 2.06 |
|  |  | *dcam* | S-adenosylmethionine decarboxylase proenzyme | 2.61 | 1.43 | 2.49 | 2.40 | 1.99 | 1.82 | 2.36 | 2.43 |
|  |  | LOC106601716 | aldehyde dehydrogenase family 3 member B1-like | 2.54 | 1.86 | 2.82 | 2.96 | 2.62 | 2.62 | 3.04 | 2.91 |
|  |  | LOC100194597 | adenosylhomocysteinase | 2.53 | 1.34 | 2.37 | 1.85 | 1.82 | 1.42 | 1.48 | 1.67 |
|  |  | acot7 | acyl-CoA thioesterase 7 | 2.49 | 1.54 | 2.17 | 2.26 | 2.73 | 2.18 | 1.88 | 2.85 |
|  |  | LOC106596374 | aldehyde dehydrogenase family 3 member B1-like | 2.48 | 1.68 | 2.43 | 2.94 | 2.76 | 2.51 | 2.79 | 2.84 |
|  |  | LOC106580346 | S-adenosylmethionine synthase | 2.48 | 1.57 | 2.56 | 2.67 | 3.69 | 3.38 | 3.52 | 4.75 |
|  |  | LOC106590260 | phosphoenolpyruvate carboxykinase 2 (mitochondrial) | 2.44 | 1.34 | 2.96 | 2.95 | 2.86 | 2.96 | 2.81 | 3.38 |
|  |  | *pudp* | pseudouridine 5'-phosphatase | 2.43 | 1.33 | 2.76 | 2.36 | 2.29 | 1.76 | 2.09 | 2.26 |
|  |  | *aldh4a1* | aldehyde dehydrogenase 4 family, member A1 | 2.40 | 1.41 | 2.55 | 2.41 | 1.65 | 1.99 | 2.10 | 2.24 |
|  |  | *psat1* | phosphoserine aminotransferase 1 | 2.31 | 1.54 | 2.46 | 2.75 | 2.62 | 2.59 | 2.15 | 2.87 |
|  |  | LOC106585666 | mevalonate kinase | 2.23 | 1.72 | 2.57 | 1.91 | 1.79 | 1.71 | 1.66 | 1.29 |
|  |  | LOC106560925 | fatty acid 2-hydroxylase | 2.20 | 1.50 | 1.60 | 1.65 | 1.45 | 1.37 | 2.00 | 2.10 |
|  |  | LOC106573862 | cystathionine gamma-lyase | 2.19 | 1.38 | 2.12 | 1.98 | 1.67 | 1.54 | 2.49 | 2.71 |
|  |  | *hsd17b7* | hydroxysteroid (17-beta) dehydrogenase 7 | 2.14 | 1.75 | 2.47 | 2.65 | 1.83 | 2.03 | 1.84 | 1.45 |
|  |  | *acer1* | alkaline ceramidase 1 | 2.13 | 1.67 | 2.20 | 2.86 | 2.01 | 2.48 | 1.90 | 2.40 |
|  |  | *gfpt1* | glutamine-fructose-6-phosphate transaminase 1 | 2.09 | 1.55 | 2.07 | 1.88 | 1.66 | 1.38 | 1.83 | 1.88 |
|  |  | *uroc1* | urocanate hydratase 1 | -1.68 | -1.31 | -1.76 | -1.71 | -1.84 | -1.76 | -1.27 | -1.93 |
|  |  | LOC106605086 | GTP cyclohydrolase 1 | -2.34 | -1.97 | -2.59 | -1.34 | -1.53 | -2.21 | -1.68 | -4.50 |
| Secondary alcohol biosynthetic process | KEGG:00062; KEGG:00900; GO:0035383; GO:0004659; GO:0006720; GO:0016125; GO:0097384; GO:0008204; GO:0008299; GO:0006721; GO:0016126; GO:0046490; GO:1902653; GO:0006696; GO:0009240; GO:0016114; GO:0033865; GO:0045338; GO:0006637; GO:0006695; GO:0034032; GO:0033875; GO:0045337; GO:0006084; GO:0019287 | *ebp* | EBP cholestenol delta-isomerase | 5.02 | 3.30 | 4.74 | 4.97 | 4.77 | 3.77 | 4.55 | 4.66 |
|  |  | LOC106587139 | 7-dehydrocholesterol reductase | 4.35 | 3.29 | 4.74 | 3.75 | 4.13 | 3.41 | 3.88 | 3.81 |
|  |  | LOC106603135 | delta(14)-sterol reductase TM7SF2 | 4.34 | 2.25 | 3.88 | 3.65 | 3.98 | 3.13 | 3.78 | 3.88 |
|  |  | *lss* | lanosterol synthase (2,3-oxidosqualene-lanosterol cyclase) | 4.30 | 2.75 | 4.29 | 3.84 | 3.80 | 3.42 | 3.46 | 3.67 |
|  |  | *hmgcs1* | 3-hydroxy-3-methylglutaryl-CoA synthase 1 (soluble) | 4.10 | 3.08 | 4.21 | 3.70 | 3.38 | 3.12 | 3.27 | 3.46 |
|  |  | LOC106570829 | 3-hydroxy-3-methylglutaryl-coenzyme A reductase | 3.85 | 3.04 | 3.45 | 4.55 | 3.94 | 3.67 | 3.72 | 4.43 |
|  |  | LOC106562054 | 7-dehydrocholesterol reductase | 3.76 | 2.88 | 3.90 | 3.43 | 3.23 | 3.37 | 2.99 | 2.98 |
|  |  | LOC106588568 | lanosterol 14-alpha demethylase | 3.61 | 2.58 | 3.83 | 3.34 | 3.44 | 3.09 | 3.18 | 3.19 |
|  |  | LOC106605546 | squalene monooxygenase | 3.57 | 3.09 | 3.79 | 3.50 | 3.44 | 3.02 | 3.40 | 3.45 |
|  |  | LOC106570209 | lanosterol 14-alpha demethylase | 3.52 | 2.55 | 3.67 | 3.52 | 4.00 | 2.96 | 3.45 | 3.49 |
|  |  | LOC106605924 | farnesyl pyrophosphate synthase | 3.31 | 2.23 | 3.03 | 3.20 | 2.93 | 2.17 | 2.55 | 2.71 |
|  |  | *fpps* | Farnesyl pyrophosphate synthetase | 3.29 | 2.15 | 3.05 | 3.35 | 2.65 | 2.29 | 2.65 | 2.78 |
|  |  | LOC106590825 | methylsterol monooxygenase 1 | 3.27 | 2.64 | 3.55 | 3.23 | 3.36 | 2.86 | 3.40 | 3.39 |
|  |  | *erg19* | Diphosphomevalonate decarboxylase | 3.20 | 2.07 | 3.07 | 2.82 | 3.35 | 2.39 | 2.96 | 2.80 |
|  |  | LOC106579093 | squalene monooxygenase | 3.17 | 2.43 | 3.62 | 3.34 | 2.89 | 2.97 | 3.38 | 3.07 |
|  |  | LOC106571348 | elongation of very long chain fatty acids protein 7-like | 3.06 | 2.25 | 1.75 | 2.94 | 3.88 | 2.36 | 3.97 | 5.72 |
|  |  | *fdft1* | farnesyl-diphosphate farnesyltransferase 1 | 2.83 | 1.81 | 3.23 | 2.66 | 2.65 | 2.24 | 2.47 | 2.65 |
|  |  | *mvda* | mevalonate (diphospho) decarboxylase a | 2.81 | 1.54 | 2.66 | 2.59 | 2.14 | 1.71 | 2.27 | 2.00 |
|  |  | *sc5d* | sterol-C5-desaturase | 2.72 | 1.96 | 2.85 | 2.47 | 2.50 | 1.91 | 1.98 | 2.16 |
|  |  | LOC106592246 | acyl-coenzyme A thioesterase THEM4 | 2.65 | 1.60 | 1.41 | 2.39 | 2.40 | 2.05 | 2.65 | 2.95 |
|  |  | *acat2* | acetyl-CoA acetyltransferase 2 | 2.64 | 1.75 | 3.08 | 2.66 | 2.31 | 2.16 | 2.15 | 2.06 |
|  |  | LOC106586205 | retinol dehydrogenase 7 | 2.64 | 1.90 | 2.27 | 2.11 | 2.24 | 2.01 | 2.29 | 2.58 |
|  |  | *idi1* | isopentenyl-diphosphate delta isomerase 1 | 2.58 | 2.28 | 2.32 | 1.95 | 2.26 | 1.55 | 1.56 | 1.79 |
|  |  | *acot7* | acyl-CoA thioesterase 7 | 2.49 | 1.54 | 2.17 | 2.26 | 2.73 | 2.18 | 1.88 | 2.85 |
|  |  | LOC106585666 | mevalonate kinase | 2.23 | 1.72 | 2.57 | 1.91 | 1.79 | 1.71 | 1.66 | 1.29 |
|  |  | *hsd17b7* | hydroxysteroid (17-beta) dehydrogenase 7 | 2.14 | 1.75 | 2.47 | 2.65 | 1.83 | 2.03 | 1.84 | 1.45 |
|  |  | LOC106590115 | low-density lipoprotein receptor | 1.78 | 1.70 | 1.48 | 1.61 | 2.13 | 1.71 | 2.16 | 2.18 |
| Steroid biosynthesis | KEGG:00100; KEGG:00900; GO:0044283; GO:1901615; GO:0006066; GO:0008202; GO:0008610; GO:0016053; GO:1901617; GO:0006720; GO:0016125; GO:0016128; GO:0046165; GO:0097384; GO:1902652; GO:0006694; GO:0008204; GO:0008299; GO:0008203; GO:0016126; GO:0016129; GO:0046394; GO:1902653; GO:0006696; GO:0006695; GO:0006084 | *ebp* | EBP cholestenol delta-isomerase | 5.02 | 3.30 | 4.74 | 4.97 | 4.77 | 3.77 | 4.55 | 4.66 |
|  |  | LOC106587139 | 7-dehydrocholesterol reductase | 4.35 | 3.29 | 4.74 | 3.75 | 4.13 | 3.41 | 3.88 | 3.81 |
|  |  | LOC106603135 | delta(14)-sterol reductase TM7SF2 | 4.34 | 2.25 | 3.88 | 3.65 | 3.98 | 3.13 | 3.78 | 3.88 |
|  |  | *lss* | lanosterol synthase (2,3-oxidosqualene-lanosterol cyclase) | 4.30 | 2.75 | 4.29 | 3.84 | 3.80 | 3.42 | 3.46 | 3.67 |
|  |  | LOC106570297 | asparagine synthetase [glutamine-hydrolyzing] | 4.20 | 1.66 | 4.27 | 4.55 | 3.81 | 3.68 | 3.78 | 4.17 |
|  |  | *hmgcs1* | 3-hydroxy-3-methylglutaryl-CoA synthase 1 (soluble) | 4.10 | 3.08 | 4.21 | 3.70 | 3.38 | 3.12 | 3.27 | 3.46 |
|  |  | LOC106604248 | sterol-4-alpha-carboxylate 3-dehydrogenase, decarboxylating-like | 4.08 | 3.01 | 3.90 | 4.19 | 3.16 | 2.69 | 2.73 | 2.97 |
|  |  | *dhcr24* | 24-dehydrocholesterol reductase | 3.89 | 1.77 | 4.23 | 4.16 | 3.51 | 3.29 | 3.89 | 3.76 |
|  |  | LOC106570829 | 3-hydroxy-3-methylglutaryl-coenzyme A reductase | 3.85 | 3.04 | 3.45 | 4.55 | 3.94 | 3.67 | 3.72 | 4.43 |
|  |  | LOC106562054 | 7-dehydrocholesterol reductase | 3.76 | 2.88 | 3.90 | 3.43 | 3.23 | 3.37 | 2.99 | 2.98 |
|  |  | LOC106588568 | lanosterol 14-alpha demethylase | 3.61 | 2.58 | 3.83 | 3.34 | 3.44 | 3.09 | 3.18 | 3.19 |
|  |  | LOC106605546 | squalene monooxygenase | 3.57 | 3.09 | 3.79 | 3.50 | 3.44 | 3.02 | 3.40 | 3.45 |
|  |  | LOC106570209 | lanosterol 14-alpha demethylase | 3.52 | 2.55 | 3.67 | 3.52 | 4.00 | 2.96 | 3.45 | 3.49 |
|  |  | *ptgs2b* | prostaglandin-endoperoxide synthase 2b | 3.48 | 1.44 | 1.87 | 1.46 | 3.55 | 2.27 | 3.74 | 4.67 |
|  |  | LOC106583179 | prostacyclin synthase-like | 3.39 | 2.35 | 2.01 | 2.61 | 4.35 | 4.60 | 5.11 | 4.98 |
|  |  | LOC106605924 | farnesyl pyrophosphate synthase | 3.31 | 2.23 | 3.03 | 3.20 | 2.93 | 2.17 | 2.55 | 2.71 |
|  |  | *fpps* | Farnesyl pyrophosphate synthetase | 3.29 | 2.15 | 3.05 | 3.35 | 2.65 | 2.29 | 2.65 | 2.78 |
|  |  | LOC106590825 | methylsterol monooxygenase 1 | 3.27 | 2.64 | 3.55 | 3.23 | 3.36 | 2.86 | 3.40 | 3.39 |
|  |  | *erg19* | Diphosphomevalonate decarboxylase | 3.20 | 2.07 | 3.07 | 2.82 | 3.35 | 2.39 | 2.96 | 2.80 |
|  |  | LOC106586211 | D-3-phosphoglycerate dehydrogenase | 3.19 | 1.97 | 4.12 | 4.40 | 5.03 | 4.62 | 4.43 | 5.19 |
|  |  | LOC106579093 | squalene monooxygenase | 3.17 | 2.43 | 3.62 | 3.34 | 2.89 | 2.97 | 3.38 | 3.07 |
|  |  | LOC106612829 | phosphoserine phosphatase-like | 3.13 | 2.29 | 2.72 | 2.86 | 3.73 | 3.12 | 3.45 | 2.05 |
|  |  | LOC106571348 | elongation of very long chain fatty acids protein 7-like | 3.06 | 2.25 | 1.75 | 2.94 | 3.88 | 2.36 | 3.97 | 5.72 |
|  |  | LOC106612199 | sterol-4-alpha-carboxylate 3-dehydrogenase, decarboxylating | 2.89 | 1.59 | 2.85 | 2.70 | 2.07 | 1.53 | 1.70 | 2.07 |
|  |  | LOC106586335 | isocitrate dehydrogenase [NADP] cytoplasmic | 2.88 | 1.46 | 2.69 | 2.87 | 2.82 | 2.48 | 2.98 | 3.28 |
|  |  | LOC106588251 | sphingosine kinase 1-like | 2.87 | 2.32 | 1.68 | 2.53 | 3.48 | 3.70 | 3.59 | 4.52 |
|  |  | *fdft1* | farnesyl-diphosphate farnesyltransferase 1 | 2.83 | 1.81 | 3.23 | 2.66 | 2.65 | 2.24 | 2.47 | 2.65 |
|  |  | *mvda* | mevalonate (diphospho) decarboxylase a | 2.81 | 1.54 | 2.66 | 2.59 | 2.14 | 1.71 | 2.27 | 2.00 |
|  |  | LOC106603795 | sphingolipid delta(4)-desaturase/C4-monooxygenase DES2 | 2.78 | 1.40 | 1.68 | 2.37 | 2.49 | 2.69 | 3.19 | 3.39 |
|  |  | *sc5d* | sterol-C5-desaturase | 2.72 | 1.96 | 2.85 | 2.47 | 2.50 | 1.91 | 1.98 | 2.16 |
|  |  | LOC106585939 | endothelial lipase-like | 2.69 | 1.41 | 1.60 | 1.96 | 2.62 | 2.37 | 2.52 | 3.37 |
|  |  | LOC106562243 | aspartate aminotransferase, mitochondrial | 2.65 | 1.27 | 2.94 | 2.71 | 2.93 | 2.54 | 2.84 | 2.84 |
|  |  | LOC106602973 | GTP cyclohydrolase 1 | 2.65 | 2.25 | 2.51 | 1.96 | 2.85 | 2.85 | 2.82 | 3.43 |
|  |  | *acat2* | acetyl-CoA acetyltransferase 2 | 2.64 | 1.75 | 3.08 | 2.66 | 2.31 | 2.16 | 2.15 | 2.06 |
|  |  | LOC106586205 | retinol dehydrogenase 7 | 2.64 | 1.90 | 2.27 | 2.11 | 2.24 | 2.01 | 2.29 | 2.58 |
|  |  | LOC106565623 | ethanolamine kinase 1-like | 2.61 | 1.47 | 1.75 | 2.08 | 2.36 | 1.94 | 2.66 | 2.62 |
|  |  | *idi1* | isopentenyl-diphosphate delta isomerase 1 | 2.58 | 2.28 | 2.32 | 1.95 | 2.26 | 1.55 | 1.56 | 1.79 |
|  |  | *acot7* | acyl-CoA thioesterase 7 | 2.49 | 1.54 | 2.17 | 2.26 | 2.73 | 2.18 | 1.88 | 2.85 |
|  |  | LOC106590260 | phosphoenolpyruvate carboxykinase 2 (mitochondrial) | 2.44 | 1.34 | 2.96 | 2.95 | 2.86 | 2.96 | 2.81 | 3.38 |
|  |  | *pudp* | pseudouridine 5'-phosphatase | 2.43 | 1.33 | 2.76 | 2.36 | 2.29 | 1.76 | 2.09 | 2.26 |
|  |  | *psat1* | phosphoserine aminotransferase 1 | 2.31 | 1.54 | 2.46 | 2.75 | 2.62 | 2.59 | 2.15 | 2.87 |
|  |  | LOC106585666 | mevalonate kinase | 2.23 | 1.72 | 2.57 | 1.91 | 1.79 | 1.71 | 1.66 | 1.29 |
|  |  | LOC106560925 | fatty acid 2-hydroxylase | 2.20 | 1.50 | 1.60 | 1.65 | 1.45 | 1.37 | 2.00 | 2.10 |
|  |  | LOC106573862 | cystathionine gamma-lyase | 2.19 | 1.38 | 2.12 | 1.98 | 1.67 | 1.54 | 2.49 | 2.71 |
|  |  | *hsd17b7* | hydroxysteroid (17-beta) dehydrogenase 7 | 2.14 | 1.75 | 2.47 | 2.65 | 1.83 | 2.03 | 1.84 | 1.45 |
|  |  | *acer1* | alkaline ceramidase 1 | 2.13 | 1.67 | 2.20 | 2.86 | 2.01 | 2.48 | 1.90 | 2.40 |
|  |  | LOC106590115 | low-density lipoprotein receptor | 1.78 | 1.70 | 1.48 | 1.61 | 2.13 | 1.71 | 2.16 | 2.18 |
|  |  | LOC106593218 | suppressor of cytokine signaling 1-like | 1.47 | 1.49 | 2.12 | 3.51 | 2.72 | 3.81 | 2.50 | 3.60 |
|  |  | LOC106605086 | GTP cyclohydrolase 1 | -2.34 | -1.97 | -2.59 | -1.34 | -1.53 | -2.21 | -1.68 | -4.50 |
|  |  | LOC106590888 | 3-oxo-5-alpha-steroid 4-dehydrogenase 2-like | -4.83 | -1.56 | -4.25 | -3.31 | -1.83 | -1.99 | -3.14 | -2.37 |
