# Supplementary material for: Sea lice (Lepeophtheirus salmonis) life stage impacts atlantic salmon transcriptomic responses under different thermal profiles
Source: Front Genet. 2025 Jul 29;16:1633603. doi: 10.3389/fgene.2025.1633603 (PMC12339338; doi:10.3389/fgene.2025.1633603)
Supplement: Supplementary file 13 [file DataSheet1.docx]

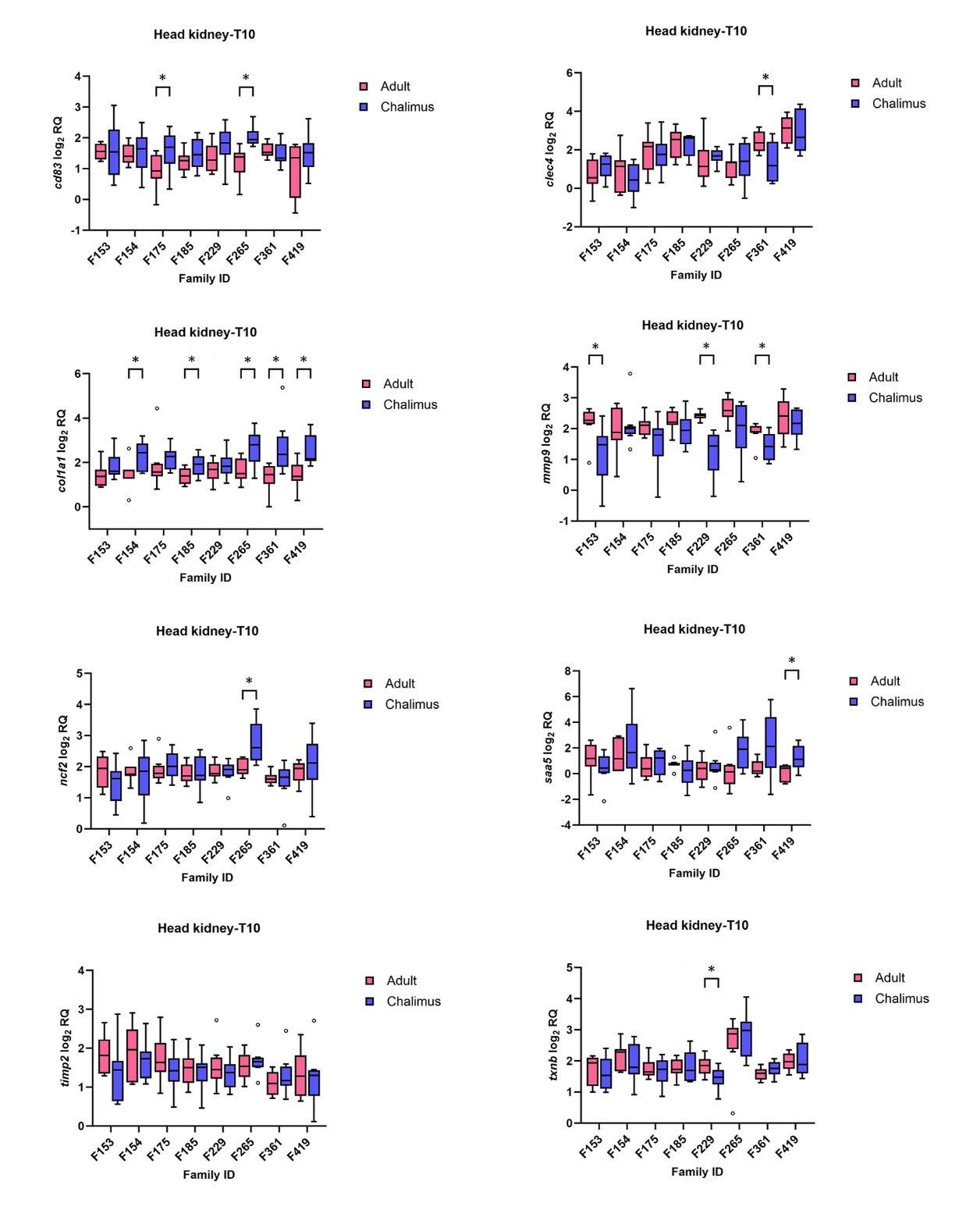


**Supplementary Figure S1**. qPCR results for genes of interest (GOIs) in the head kidney of different families infected with adult versus chalimus stages of sea lice at 10 °C. Data are presented as box plots with median and Tukey whiskers (fences). Significant differences in gene expression responses between infection stages within each family are indicated by asterisks.


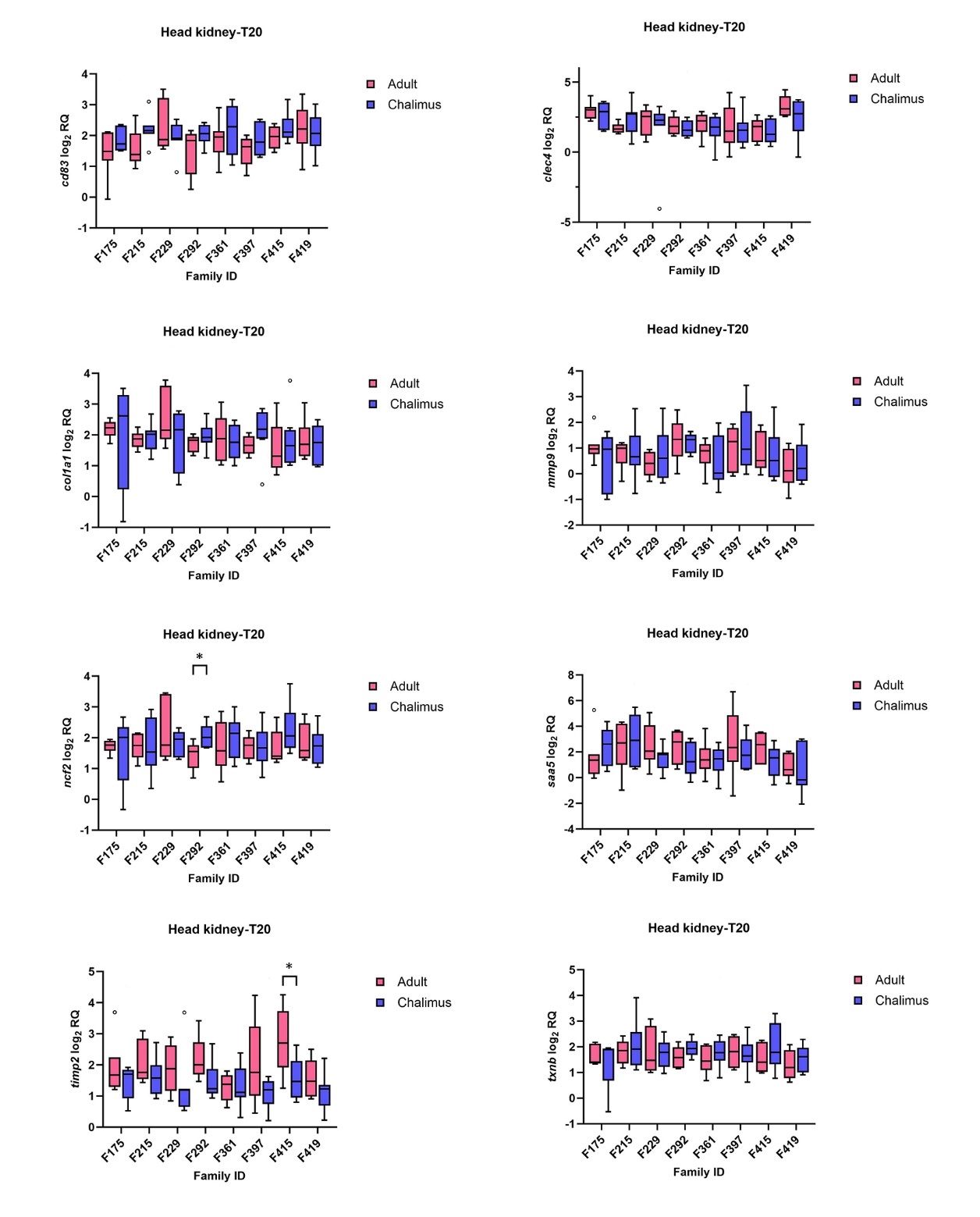


**Supplementary Figure S2**. qPCR results for genes of interest (GOIs) in the head kidney of different families infected with adult versus chalimus stages of sea lice at 20 °C. Data are presented as box plots with median and Tukey whiskers (fences). Significant differences in gene expression responses between infection stages within each family are indicated by asterisks.


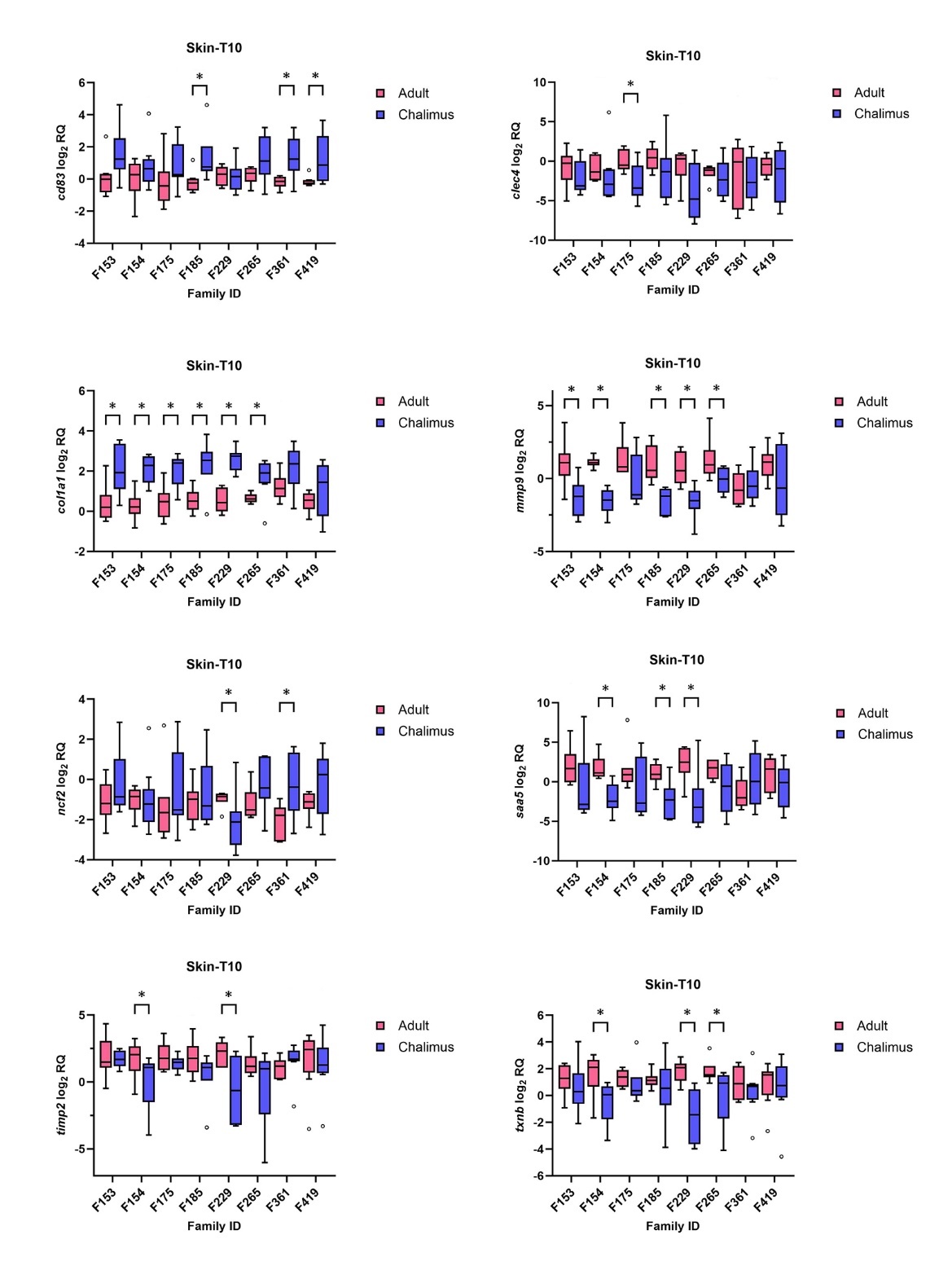


**Supplementary Figure S3**. qPCR results for genes of interest (GOIs) in the skin of different families infected with adult versus chalimus stages of sea lice at 10 °C. Data are presented as box plots with median and Tukey whiskers (fences). Significant differences in gene expression responses between infection stages within each family are indicated by asterisks.


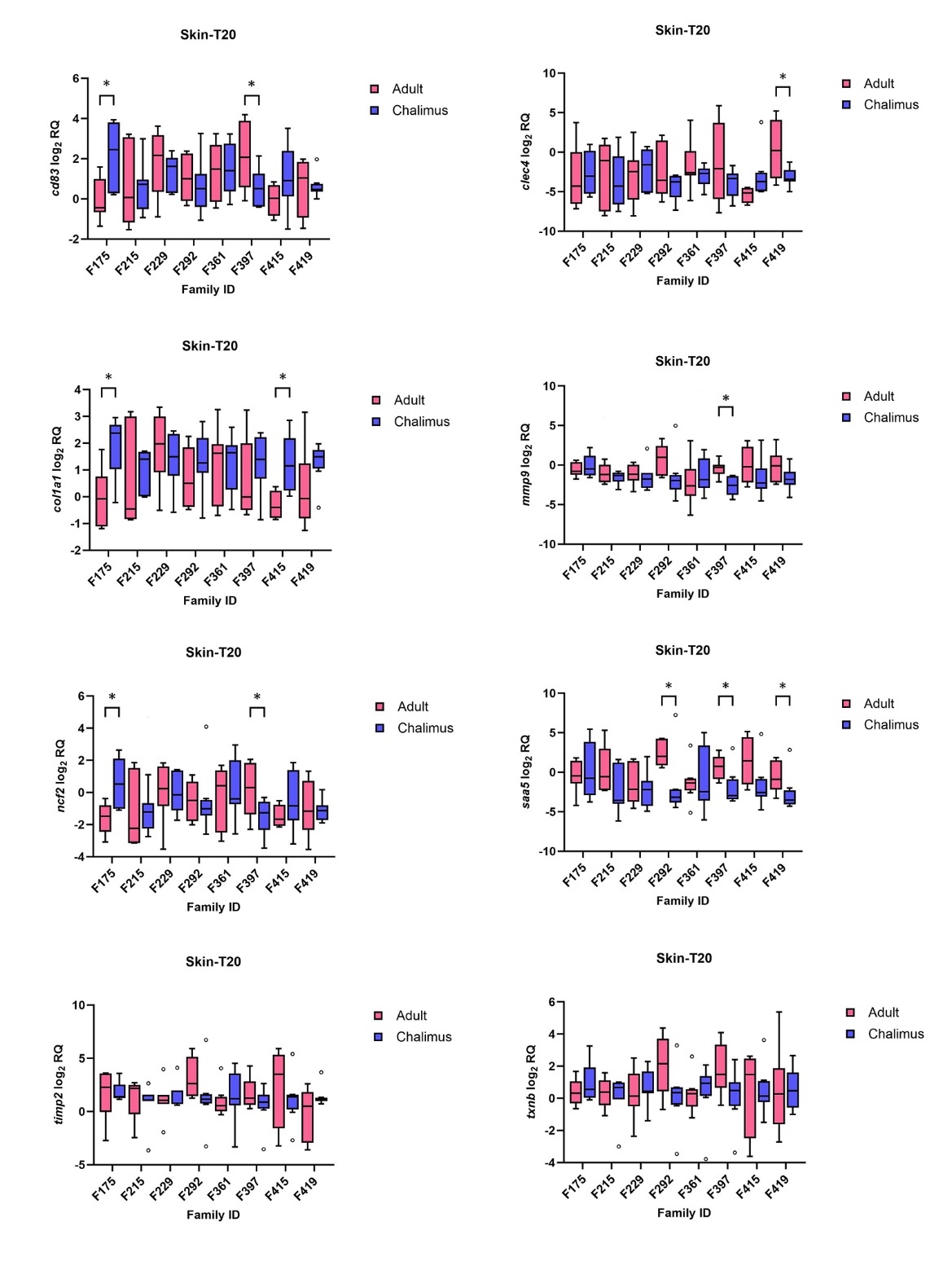


**Supplementary Figure S4**. qPCR results for genes of interest (GOIs) in the skin of different families infected with adult versus chalimus stages of sea lice at 20 °C. Data are presented as box plots with median and Tukey whiskers (fences). Significant differences in gene expression responses between infection stages within each family are indicated by asterisks.
